# In Situ Assembly of Transformable Monopeptide on Activated Neutrophils Attenuates NETs‐Induced Hepatocellular Carcinoma Metastasis by Disrupting NE Nuclear Translocation

**DOI:** 10.1002/advs.202517415

**Published:** 2025-10-24

**Authors:** Yichi Chen, Yijun Wang, Haitao Shang, Jiayue Qiu, Ruotian Zhang, Yuxiang Xiong, Tong Wang, Fengyi Wang, Anbang Wu, Xin Lin, Bolin Wu, Chen Huang, Wen Cheng, Lu Zhang

**Affiliations:** ^1^ Guangdong Provincial Key Laboratory of Advanced Biomaterials Department of Biomedical Engineering Southern University of Science and Technology Shenzhen 518055 China; ^2^ Department of Ultrasound Harbin Medical University Cancer Hospital Harbin 150081 China; ^3^ Heilongjiang Province Key Laboratory of Research on Molecular Targeted Anti‐Tumor Drugs Harbin 150081 China; ^4^ Faculty of Chinese Medicine & State Key Laboratory of Mechanism and Quality of Chinese Medicine, Dr. Neher’s Biophysics Laboratory for Innovative Drug Discovery Macau University of Science and Technology Taipa Macau SAR 999078 China

**Keywords:** hepatocellular carcinoma metastasis, in vivo assembly, neutrophil elastase, neutrophil extracellular traps, transformable peptide

## Abstract

Neutrophil extracellular traps (NETs) released by activated neutrophils in the tumor microenvironment has emerged as a pivotal mediator in promoting tumor metastasis. The alteration of the subcellular localization of neutrophil elastase (NE) is crucial for NETs formation. The majority of NE (≈80%) translocate from azurophilic granules to the nucleus, facilitating histone degradation and chromatin decondensation. A few NE are transported to the cell membrane, a unique feature of activated neutrophils that distinguishes them from other leukocyte subpopulations. To address NETs‐mediated HCC metastasis, a peptidic nanomaterial (FTP‐NPs) is developed that specifically binds NE on activated neutrophil membranes and undergoes in situ fibrillar transformation, forming NE‐fibril clusters. These NE‐fibril clusters deactivate NE by altering their conformation or binding mode. Subsequently, a series of feedback mechanisms is triggered, which regulates NE membrane concentration by promoting its transport to the membrane rather than the nucleus. The NE‐fibril clusters can remain on the activated neutrophil membrane for an extended period, enabling continuous binding and deactivation of newly transported NE, thereby reversing the formation of NETs. Besides, the extracellular NE‐fibril clusters also act as a physical barrier to prevent NETs from adhering to tumor cells, further disrupting the metastatic cascade. In vitro, in vivo, and single‐cell RNA sequencing (scRNA‐seq) data confirm that FTP‐NPs significantly reduce NETs formation, reduce metastatic burden, and enhance antitumor immune response. Compared with commercial NE inhibitors, this strategy precisely and locally regulates NE subcellular distribution within neutrophils in tumor tissue, minimizing off‐target effects and systemic toxicity. The NE‐fibril clusters may establish an innovative therapeutic approach for NETs‐mediated tumor metastasis.

## Graphical abstract

Activated neutrophils release extracellular traps (NETs) that promote tumor metastasis. FTP‐NPs are develpoed, a peptidic nanomaterial that binds NE on neutrophil membranes, forming NE‐fibril clusters that deactivate NE and prevent NETs formation. This strategy reduces metastasis and enhances the anti‐tumor immune response by targeting NE subcellular distribution within neutrophils in tumor tissue, offering a precise and low‐toxicity approach for NETs‐mediated metastasis.

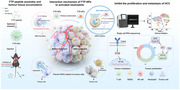

## 1 Introduction

Tumor metastasis remains the primary cause of cancer‐related mortality and encompasses intricate interactions between tumor cells and their surrounding microenvironment. Among these interactions, the neutrophil extracellular traps (NETs) released by activated neutrophils has emerged as a pivotal mediator in significantly promoting tumor cell proliferation, migration, invasion, and angiogenesis.^[^ ^1^ ^]^ Elevated levels of NETs have been consistently observed in patients with middle and advanced‐stage cancers and are significantly associated with an increased metastatic burden as well as a poor clinical prognosis. NETs are web‐like structures composed of decondensed chromatin and cytotoxic proteins, such as neutrophil elastase (NE) and myeloperoxidase (MPO).^[^ ^2^ ^]^ Subcellular localization of NE would be altered once neutrophils are activated within the tumor microenvironment. I) The majority of NE (≈80%) translocate from azurophilic granules to the nucleus, facilitating histone degradation and chromatin decondensation, which constitutes a pivotal step in the formation of NETs;^[^ ^3^ ^]^ II) A few NE are transported to the cell membrane, which is a unique feature of activated neutrophils that distinguishes them from other leukocyte subpopulations.

Current NE inhibition strategies, such as synthetic inhibitors and monoclonal antibodies, are frequently constrained by several limitations.^[^ ^4^ ^]^ These include insufficient specificity, substantial off‐target effects, and the inability to precisely regulate the spatial localization of NE within neutrophils, thereby limiting their therapeutic efficacy.^[^ ^5^ ^]^ Moreover, systemic administration of NE inhibitors may result in adverse side effects, including the suppression of essential immune functions.^[^ ^6^ ^]^ There is a pressing need for innovative strategies that can selectively target NE in the tumor microenvironment while minimizing systemic toxicity. Currently, programs to specifically and non‐invasively regulate the subcellular distribution and activity of NE in activated neutrophils are lacking.

Advances in nanotechnology have revolutionized cancer therapy by offering precise and adaptable treatment strategies.^[^ ^7^ ^]^ Among these, in situ transformable nanomaterials in vivo have shown great promise due to their ability to respond dynamically to pathological stimuli in the tumor microenvironment (TME).^[^ ^8^, ^9^ ^]^ These materials without any drugs can undergo structural transformations in response to specific triggers, enhancing the specific cell selectivity in vivo and the therapeutic efficacy.^[^ ^10^ ^]^ For neutrophil‐targeted therapies, ≈20% of NE are exclusively expressed on the surface of activated neutrophils and are undetectable in other leukocyte subsets, making them an excellent target for neutrophil‐specific drug development.^[^ ^11^ ^]^ In situ transformable materials could exploit the unique biochemical and physical characteristics of activated neutrophils to achieve selective targeting and dynamic modulation of pathological processes in vivo, such as NEs nuclear translocation.^[^ ^12^, ^13^, ^14^ ^]^

**Scheme 1:**
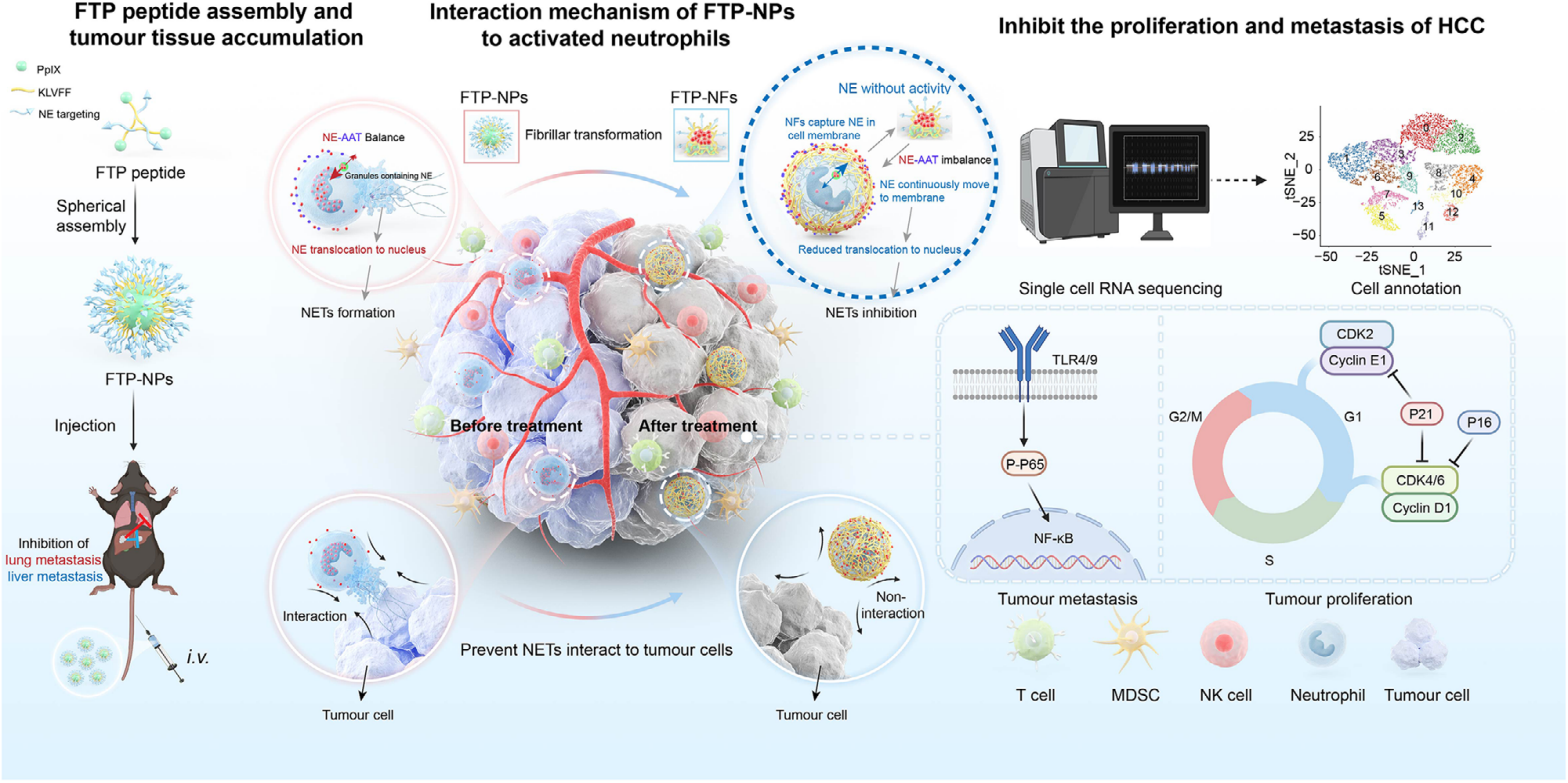
In situ fibrillar cluster in vivo specifically regulates the distribution and enzymatic activity of elastase within neutrophils, inhibiting NETs formation to counteract hepatocellular carcinoma metastasis. Schematic illustration of the spherical self‐assembly, tumor accumulation, and in situ fibrillation transformation of FTP‐NPs in tumor tissues to promote NEs externalization/deactivation and reduce NEs nuclear translocation, followed by ScRNA‐seq to reveal anti‐metastatic and anti‐proliferative events in tumor cells through NETs reduction.

Here, we developed a peptidic nanomaterial that specifically regulated the subcellular distribution and enzymatic activity of elastase within neutrophils, inhibiting NETs formation against hepatocellular carcinoma (HCC) metastasis (**Scheme** **1**). This fibril‐transformable monopeptide (FTP, PpIX‐GFFVLK‐EAIPMSIPPEVK) was composed of three modular functional domains: 1) the NEs targeting domain comprised of a linear and high‐affinity peptide sequence EAIPMSIPPEVK;^[^ ^11^ ^]^ 2) the KLVFF β‐sheet forming peptide domain originated from β‐amyloid (Aβ) peptide,^[^ ^15^, ^16^, ^17^ ^]^ and 3) the Protoporphyrin (PpIX) moiety, as a hydrophobic core to induce the formation of micellar and fluorescence reporting. Under the aqueous conditions, FTP would be self‐assembled into spherical nanoparticles (FTP‐NPs), in which PpIX and FFVLK domains constituted the hydrophobic core, and the EAIPMSIPPEVK peptide constituted the hydrophilic corona. FTP‐NPs specifically bound NEs on the membrane of activated neutrophils and underwent in situ fibrillar transformation, forming NE‐fibril clusters in the tumor microenvironment. These NE‐fibril clusters deactivated NE by altering their conformation or binding mode. Subsequently, a series of feedback mechanisms was triggered, which regulated NE membrane concentration by promoting its transport to the membrane rather than to the nucleus. The NE‐fibril clusters could remain on the activated neutrophil membrane for an extended period, enabling continuous binding and deactivation of newly transported NE, thereby reversing the formation of NETs. Besides, the extracellular NE‐fibril clusters also acted as a physical barrier to prevent NETs from adhering to tumor cells, further disrupting the metastatic cascade. Compared with commercial NE inhibitors, this strategy precisely and locally regulates NE subcellular distribution within neutrophils in tumor tissue, minimizing off‐target effects and systemic toxicity.

## 2 Results and Discussion

### 2.1 Construction and Validation of the NET Risk Score Model

**Figure 1:**
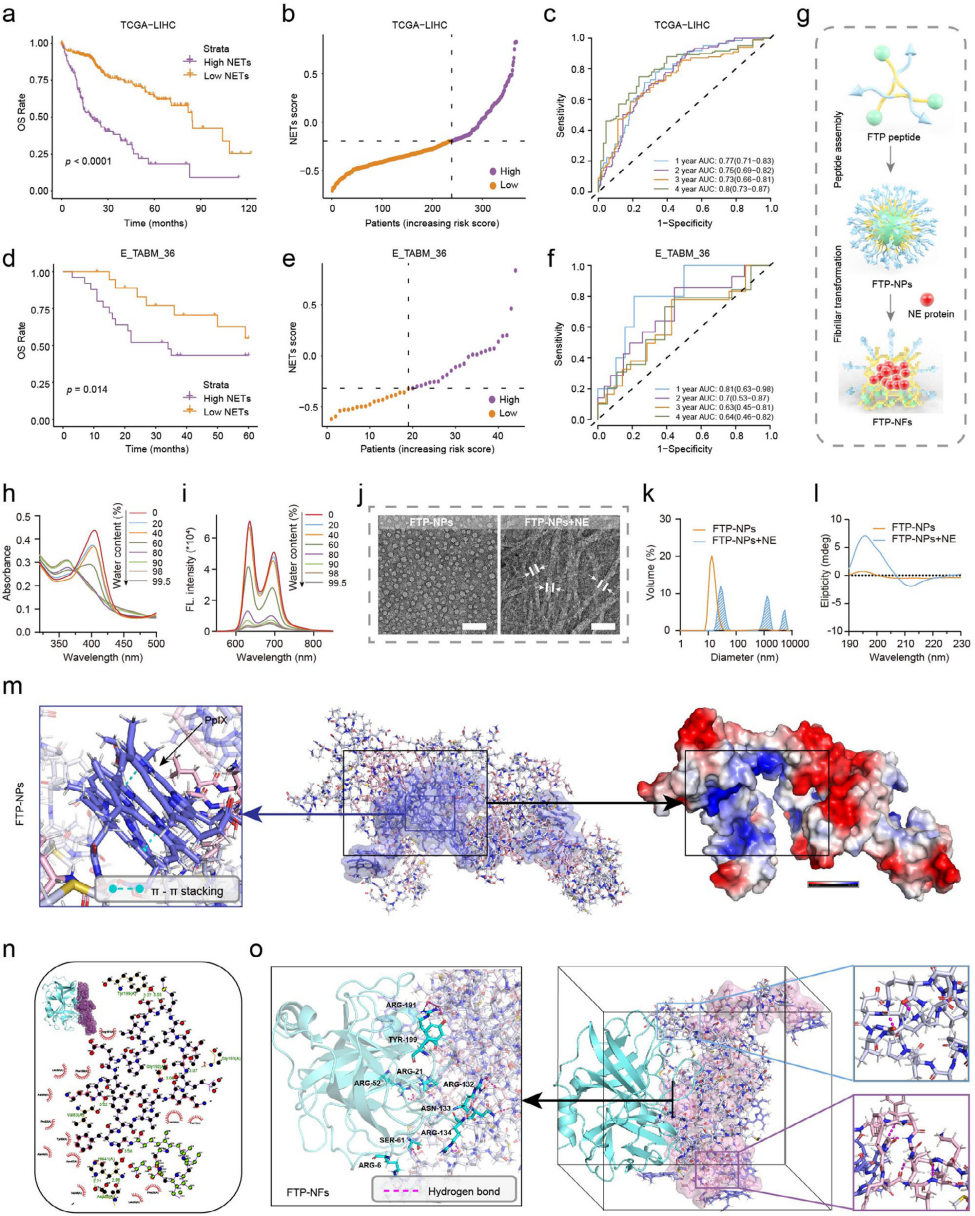
NETs risk score of HCC, spherical assembly, and fibrillar transformation of FTP in vitro. a) Comparison of OS between the low‐NETs and high‐NETs groups in the TCGA‐LIHC cohort, the *p*‐values were calculated with the log‐rank test. b) The distribution of NETs scores in the TCGA‐LIHC cohort. c) ROC curve of the NETs scoring model for predicting the OS of patients from the TCGA‐LIHC cohort. d) Comparison of OS between the low‐NETs and high‐NETs groups in the E‐TABM‐36 cohort, the *p*‐values were calculated with the log‐rank test. e) The distribution of NETs scores in the E‐TABM‐36 cohort. f) ROC curve of the NETs scoring model for predicting the OS of patients from the E‐TABM‐36 cohort. g) Schematic illustration of self‐assembly and in situ structural transformation of FTP‐NPs. h,i) Changes in UV–vis absorption and fluorescence of FTP‐NPs following the gradual addition of water (from 0 to 99.5%) to a solution of NPs in DMSO. Experiments were repeated three times. FL., fluorescence. a.u., arbitrary units. j) TEM images of initial FTP‐NPs transformation into FTP‐NFs after interaction with NEs (Mw≈30 KDa) at 24 h. Scale bars, 100 nm. Experiments were repeated three times. k,l) Variation in size distribution and CD spectra of initial NPs and NFs at 24 h. The molar ratio of NEs/peptide ligand was≈1/1000. mdeg, millidegrees. m) The self‐assembly of FTP monomers, with the central region representing the hydrophobic domain of FTP. The blue surface in the center indicates the hydrophobic region. The left side shows the original porphyrin π–π stacking structure; the right side shows an electrostatic potential map of the self‐assembled structure, with the hydrophobic region of the original porphyrin carrying a positive charge. n) The highest scoring conformation of FTP molecule docking and its 2D interaction schematic. The cyan‐colored cartoon represents NEs, and the dots represent the FTP ligand. o) Residues interacting between NE (P08246) and FTP (left), and interactions between the targeted peptide induced by the protein (top right) and the fiber‐transforming peptide (bottom right).

To explore the association between NETs and HCC, we developed a NETs‐related risk scoring model (NETs score) based on NETs biomarkers in the TCGA‐LIHC cohort. Patients were stratified into high‐NETs and low‐NETs groups according to an optimal cutoff value. Kaplan‐Meier survival analysis revealed that patients in the low‐NETs group exhibited significantly better overall survival (OS) compared to the high‐NETs group (log‐rank test, *p*‐value < 0.0001; **Figure** **1**a). The distribution plot of NET scores illustrated an increasing proportion of high‐risk patients as the NETs score increased (Figure 1b). Time‐dependent ROC analysis for OS demonstrated robust predictive performance, with area under the curve (AUC) values of 0.77, 0.75, 0.73, and 0.80 at 1, 2, 3, and 4 years, respectively, in the TCGA cohort (Figure 1c). Similar results were observed in the independent validation cohort (E‐TABM‐36), where patients in the low‐NETs group also showed significantly improved survival (log‐rank test, *p*‐value = 0.014; Figure 1d,e). ROC analysis further confirmed the stability and reliability of the NETs score, achieving AUC values of 0.81, 0.70, 0.63, and 0.64 at 1, 2, 3, and 4 years, respectively, in the validation set (Figure 1f).

To further explore the relationship between the NETs score and the tumor immune microenvironment (TIME) in HCC, we quantified immune cell infiltration levels using multiple computational methods. Interestingly, immune‐activating cell populations, such as CD4^+^ T cells and CD8^+^ T cells, demonstrated significantly higher infiltration abundance in the low‐NETs group compared to that in the high‐NETs group (Figure S1a, Supporting Information). The NETs score exhibited a significant negative correlation with the infiltration levels of these immune effector cells (Figure S1b,c, Supporting Information). Conversely, neutrophil recruitment scores and the expression levels of immune checkpoint molecules were notably elevated in the high‐NETs group compared with the low‐NETs group, indicating that a higher NETs score tends to represent a more immunosuppressive TIME, potentially facilitating tumor immune escape and contributing to HCC progression (Figure S1d,e, Supporting Information). Taken together, these findings suggest that the NETs score might not only serve as a prognostic biomarker but also reflect underlying immunological alterations that influence the tumor immune landscape and clinical outcomes.

### 2.2 Initial Assembly and Fibrillar Transformation of FTP In Vitro

The fibrillar transformable peptide FTP (PpIX‐GFFVLK‐EAIPMSIPPEVK) and non‐fibrillar transformable peptide (NFTP, PpIX‐GGGAAK‐EAIPMSIPPEVK) were synthesized and characterized (Figure 1g; Figure S2a,b, Supporting Information). As the proportion of water in the mixed solvent (water and DMSO) of the FTP solution was increased, there was a gradual decrease in absorption peaks (400‐410 nm) reflecting the gradual formation of FTP‐NPs via self‐assembly (Figure 1h). Concomitantly, the fluorescence peak at 635 nm was found to decrease dramatically due to the aggregation‐induced quenching (ACQ) fluorescence properties of PpIX dye (Figure 1i). NFTP showed similar self‐assembling properties (Figure S3a,b, Supporting Information). The dynamic light scattering (DLS) and transmission electron microscopy (TEM) showed that the diameters of FTP‐NPs and NFTP‐NPs were 24.27±4.50 nm and 26.13±3.23 nm, respectively (Figure 1j,k; Figure S3c,d, Supporting Information). The size of FTP‐NPs and NFTP‐NPs did not change significantly in PBS and FBS solutions within 168 h, indicating excellent stability (Figure S3e,f, Supporting Information). The critical aggregation concentration (CAC) of NFTP‐NPs and FTP‐NPs was calculated as 3.75 and 5.45 µm, respectively (Figure S3g,h, Supporting Information).

Several studies have shown that the interaction between ligands and receptors could cause morphological changes in nanostructures.^[^ ^18^, ^19^ ^]^ Prior to interacting with water‐soluble NEs in an aqueous solution, FTP‐NPs maintained a spherical shape at a diameter of ≈25 nm (Figure 1j). Following a 24‐h incubation period at room temperature with NEs, a few nanofibrillar structures were visible. However, there was no change in the formulation of NFTP‐NPs when NE was added (Figure S3c,d, Supporting Information). DLS further verified the morphological transformation of FTP‐NPs to FTP‐NFs in solution (Figure 1k). Besides, the zeta potential of FTP‐NPs changed from 21.7±0.6 to 9.2±0.2 mV during the fibrillar transformation (Figure S3i, Supporting Information). These findings verify that NE initiates fibrillar transformation from initial spherical FTP‐NPs in the presence of the FFVLK motif.

To further confirm this structural transition, circular dichroism (CD) spectroscopy was employed to investigate the secondary structural assembly. As shown in Figure 1l, a positive signal at 195 nm and a negative signal at 216 nm progressively developed during the transformation process, indicative of the formation of β‐sheet structures. In contrast, no significant alterations in the CD signals were observed for NFTP‐NPs (Figure S3j, Supporting Information). The transformable kinetics of FTP‐NPs and NFTP‐NPs were monitored via the fluorescence intensity changes of PpIX (Figure S3k, Supporting Information). Upon the addition of NE protein, the fluorescence intensity of PpIX in the FTP‐NPs group further decreased to 78.3% after 24 h incubation.

### 2.3 Molecular Dynamics (MD) Simulation of FTP‐NPs Assembly and Fibrillar Transformation

The 3D structures of FTP and NFTP were predicted using AlphaFold II, and then the self‐assembly behavior in an aqueous environment was further investigated through MD simulations (Figure S4a–d, Supporting Information). The analysis of electrostatic potential maps revealed the correspondence between the hydrophilic and hydrophobic regions in the assembly and the high and low electrostatic potential areas in the electrostatic maps. Water molecules typically tend to associate with negatively charged regions, which results in the hydrophobic core formed by PpIX being encapsulated by the targeting peptides and shielded from water molecules. This observation was supported by a significant decrease in the solvent‐accessible surface area (Figure S4e, Supporting Information). The reduction in the surface‐area‐to‐volume ratio indicates that the peptides gradually transition from an extended conformation to a more compact aggregate, ultimately forming more stable spherical NPs. Besides, the interactions between PpIX molecules exhibited an ordered parallel arrangement, with a parallel distance ranging from 3.6 to 4.2 Å, forming distinct π‐π stacking interactions (Figure 1m; Figure S4f, Supporting Information). PpIX molecules not only drive the self‐assembly of peptides through hydrophobic interactions but also stabilize the assembled structure via π–π stacking interactions, highlighting the crucial role of hydrophobic forces in both peptide aggregation and structural stability.

The targeting and fibrillar transformable capability of NPs induced by NE was further investigated. The molecular docking of FTP and NFTP with NE was performed, and the highest affinity conformations were selected from the docking results for subsequent molecular dynamics simulations (Figure 1n; Figure S5a–c and Table S1, Supporting Information). Notably, when combined with FTP‐NPs, the root‐mean‐square deviation (RMSD) of NE was even smaller, suggesting that FTP‐NPs might bind to NE more quickly and form a more stable self‐assembly (Figure S6a,b, Supporting Information). Root Mean Square Fluctuation (RMSF) analysis was conducted to further evaluate the flexibility of NE protein residues. The NE residues interacting with FTP and NFTP exhibited significant fluctuations, indicating that these residues are key sites for peptide‐protein interactions. Regions with higher RMSF values corresponded well with the docking results, suggesting that the binding of the peptide to NE protein not only enhances the flexibility of these regions but also directly involves these critical sites (Figure S6c, Supporting Information). Hydrogen bond analysis revealed that both FTP and NFTP formed stable hydrogen bonds with the NE protein, particularly the hydrogen bonds between ARG‐191 and TYR‐199 (Figure S6d, Supporting Information). Notably, the number of hydrogen bonds formed between FTP under the induction of NE was more than that between NFTP, contributing more effectively to the formation of fibrillar networks (Figure S6e, Supporting Information). Further analysis showed that the hydrogen bonds between NFTP molecules were primarily facilitated by the NE targeting peptides. In contrast, the hydrogen bonding in FTP molecules involved contributions from the NE targeting peptides as well, but was mainly driven by the FFVLK fibril conversion sequence (Figure 1o; Figure S6f, Supporting Information).

### 2.4 Specific Recognition of Activated Neutrophils by FTP‐NPs In Vitro

To explore the specific recognition ability of FTP‐NPs toward the activated neutrophils in vitro, phorbol‐12‐myristate‐13‐acetate (PMA, 100 nm) was employed to activate neutrophils. FTP‐NPs were incubated with activated neutrophils or resting neutrophils for 1 h. The flow cytometry results showed that the PpIX fluorescence displayed by activated neutrophils treated with FTP‐NPs was about 2.8 times that of resting neutrophils (Figure S7a,b, Supporting Information). This phenomenon may be attributed to the high expression of NEs on the surface of activated neutrophils, which facilitates their binding to FTP‐NPs and subsequent formation of FTP‐NFs. In contrast, resting neutrophils exhibit relatively lower expression of NE on their surfaces, presenting a small number of passive cellular uptake of FTP‐NPs. Subsequently, the cytotoxicity on activated neutrophils, resting neutrophils, and Hepa1‐6/luc murine HCC cells was examined using a CCK‐8 assay to evaluate the biocompatibility of FTP‐NPs. All groups showed a survival rate of over 95%, demonstrating the good safety and biocompatibility of FTP‐NPs (Figure S8a–c, Supporting Information).

### 2.5 The Fibrillar Transformation and Retention of FTP‐NPs on the Activated Neutrophil Membrane

**Figure 2:**
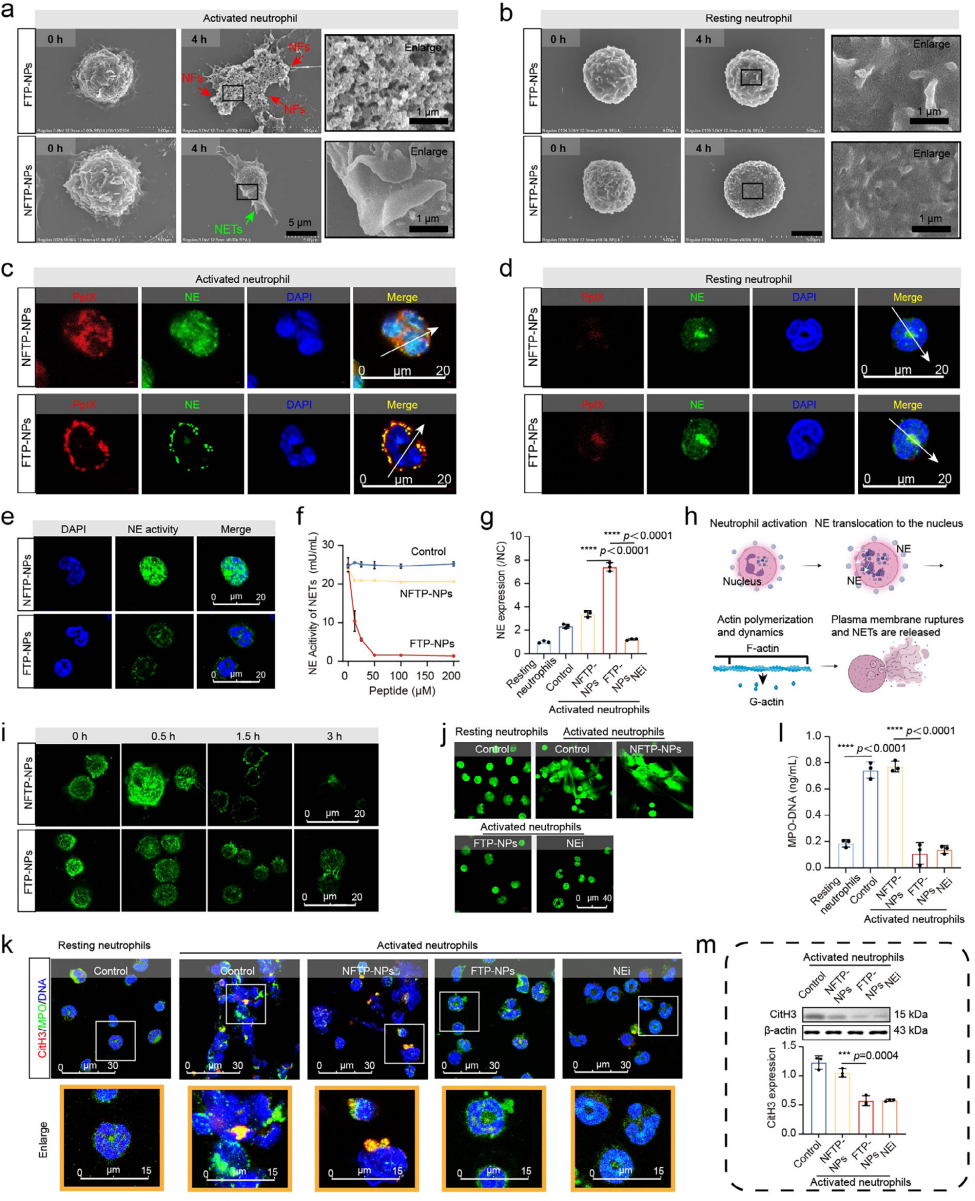
FTP‐NPs inhibit the formation of NETs and their related components. a,b) SEM images of resting neutrophils and activated neutrophils treated with NFTP‐NPs and FTP‐NPs for 4 h. Scale bars, 5 µm. The concentration of NFTP‐NPs and FTP‐NPs was 50 µm. Experiments were repeated three times. c,d) Fluorescence binding distribution images of the nano fibrillar network and NE antibody on the cell membrane of resting neutrophils and activated neutrophils. NE antibody was used to label NE, and the quantitative analysis was performed. Scale bar, 20 µm. The concentration of FTP‐NPs was 50 µm. Experiments were repeated three times. e) Enzyme activity of NE on the neutrophil membrane after FTP‐NPs. NE was labeled with NEs activity probe (green). Scale bar, 20 µm. FTP‐NPs concentration was 50 µm. The experiment was repeated three times. f) Quantitative detection of NE enzyme activity on the cell surface after different treatments. g) NE levels on the surface of neutrophils after different treatments. Data are presented as mean ± SD, *n* = 3 independent experiments. Statistical significance was calculated by one‐way ANOVA, ****p < 0.0001. h) Schematic model of the phases of NETs. Upon neutrophil activation, actin polymerization is induced. These dynamic actin rearrangements are necessary for NE to translocate to the nucleus, where it can cleave nucleosomal histones. This promotes chromatin decondensation, whereupon the cytoplasmic milieu mixes with the nuclear material before the plasma membrane finally breaks down, resulting in NETs release. The scheme was created with BioRender.com. i) F‐actin levels upon PMA stimulation were examined with phalloidin (green) at the indicated time points. Scale bar = 20 µm. j) Representative fluorescent microphotograph images of NETs (SYTOX green, green). Scale bars, 40 µm. k) NETs were identified by Cit‐H3 (red), MPO (green), and DAPI (blue) in neutrophils after different treatments. Scale bar = 30 µm. l) MPO‐DNA levels in neutrophils supernatants after different treatments. Data are presented as mean ± s.d., *n* = 3 independent experiments. Statistical significance was calculated by one‐way ANOVA, ****p < 0.0001. m) Western blot analysis for CitH3 expression in neutrophils after different treatments. The fold change of densitometric ratios was normalized to the β‐actin and then compared to the control and was shown below. Data are presented as mean ± SD, *n* = 3 independent experiments. Statistical significance was calculated by one‐way ANOVA, ***p < 0.001.

Resting neutrophils and activated neutrophils were treated with NFTP‐NPs and FTP‐NPs (50 µM) for 0, 0.5, 1.5, and 4 h, respectively, and observed by scanning electron microscope (SEM) imaging. As shown in **Figure** **2**a, fibrillar structures were observed on the surface of activated neutrophils treated with FTP‐NPs, but not on those treated with NFTP‐NPs, owing to the absence of the KLVFF sequence. No fibrils were observed on the resting neutrophil surface treated by NFTP‐NPs and FTP‐NPs due to the lack of NEs expression on the resting neutrophil surface (Figure 2b; Figure S9, Supporting Information).

To confirm the binding of FTP‐NPs to NEs located on the activated neutrophils, an anti‐NE monoclonal antibody was employed, followed by a fluorescent green secondary antibody to detect NE distribution. As shown in Figure 2c, Figure S10a, Supporting Information, a strong red fluorescence signal (PpIX) was observed on the surface of activated neutrophils treated by FTP‐NPs for 4 h, rather than within the cells. The yellow fluorescence signals, as an overlap of red and green (NEs) fluorescence signals, were found around the periphery of the two adjoined cells except at the adhesion interface, which indicated the co‐localization of FTP‐NFs and NEs. In contrast, for resting neutrophils, most of the fluorescent signals were found within the cells after NFTP‐NPs and FTP‐NPs treatment for 4 h (Figure 2d; Figure S10b, Supporting Information).^[^ ^20^ ^]^ Therefore, the high expression level and membrane location of NEs are the prerequisites for the fibrillar transformation of FTP‐NPs, which determines the cellular location and distribution of NE‐fibril clusters. Importantly, in contrast to traditional NE inhibitors, this approach ensures that the therapeutic effect of NE‐fibril clusters is localized to the tumor site, thereby reducing systemic toxicity and minimizing off‐target effects.

The retention time of NE‐fibril clusters on the membrane of activated neutrophils was further investigated. The resting and activated neutrophils were treated with NFTP‐NPs and FTP‐NPs (50 µm). NFTP‐NPs or FTP‐NPs were washed off after 4 h of incubation, and then fresh medium without NFTP‐NPs or FTP‐NPs was added to incubate cells for another 8 h. As expected, the FTP‐NPs‐treated activated neutrophil group exhibited persistently strong PpIX fluorescence signals within 12 h, demonstrating the long‐term retention ability of NE‐fibril clusters (Figure S11a,b, Supporting Information). In stark contrast, the fluorescence intensities of PpIX in the NFTP‐NPs‐treated activated neutrophil group, FTP‐NPs‐treated resting neutrophil group, and NFTP‐NPs‐treated resting neutrophil group dropped sharply from 4 h to 12 h of incubation, because they underwent lysosomal degradation of neutrophils (Figure S11c,d, Supporting Information).

### 2.6 Specifically Regulates the Subcellular Location and Enzymatic Activity of Elastase in Activated Neutrophils

The EAIPMSIPPEVK targeting peptide motif in FTP‐NPs could not only bind NE but also exhibit the ability to deactivate NEs, functioning as an NE inhibitor.^[^ ^11^ ^]^ After interaction with NE, FTP‐NPs transform into the NE‐fibril clusters that bind a greater amount of NEs, leading to a more substantial reduction in NE activity. Neutrophils would initiate a series of feedback mechanisms that regulate NEs membrane concentration by transporting more NEs to the membrane, not to the nucleus, which may be related to the need for neutrophils in the immune response.^[^ ^21^ ^]^ The NE‐fibril clusters could remain in the activated neutrophil membrane for a long time, which allowed them to bind and deactivate NEs that were transported to the membrane continuously, thus reversing the NETs formation caused by NE translocation to the nucleus. As shown in Figure S12a–c, Supporting Information, the NEs (green fluorescence) overlapped with the nucleus (blue fluorescence) in activated neutrophils, indicating that NEs were mainly distributed in the nucleus in the activation state of neutrophils. However, after incubating with FTP‐NPs (red fluorescence), the majority of the NEs (green fluorescence) were observed on the activated neutrophil surface, and the amount of overlap with the nuclear (blue fluorescence) was significantly reduced (Figure 2c,d). NEs‐fibril clusters changed the subcellular localization of NEs within activated neutrophils, making them more externalized to the cell surface and less translocated to the nucleus.

More interestingly, the activity detection demonstrated that the enzymatic activity of NE in the activated neutrophils treated with FTP‐NPs was significantly reduced (as indicated by fluorescence intensity representing enzyme activity, Figure 2e). Subsequently, activated neutrophils were incubated with NFTP‐NPs and FTP‐NPs in different concentrations for 4 h to examine the activity of NEs. Quantitative analysis was carried out with a NETosis Assay Kit. As shown in Figure 2f, as the concentration of FTP‐NPs increased, the activity of NE gradually decreased and almost disappeared at 50 µm of FTP‐NPs. In comparison, NFTP‐NPs had no significant effect on NE enzymatic activity. Besides, the supernatant NE concentrations from neutrophils were also quantified by ELISA following different treatments (Resting neutrophils; Control group: activated neutrophils without treatment; NFTP‐NPs group: activated neutrophils + NFTP‐NPs; FTP‐NPs group: activated neutrophils + FTP‐NPs; NEi group: activated neutrophils + NE inhibitor Sivelestat). As shown in Figure 2g, compared to other groups, the expression of NEs on the neutrophil surface increased significantly in the FTP‐NPs group, which was contrary to the results of NE activity. The inhibition of NE enzymatic activity is related to the binding effect of NE‐fibril clusters, indicating that NE‐fibril clusters may stabilize the existence of NE by binding to NE, and inhibit its functional activity. This modification could potentially influence the nuclear transport mechanism of NE by altering its subcellular localization.

### 2.7 The Inhibition of NETs Formation and Their Related Components by NE‐Fibril Clusters

It is known that cytoskeletal rearrangement plays a key role in the formation of NETs.^[^ ^22^ ^]^ Cytoskeletal rearrangement not only provides structural support for the formation of NETs, but also participates in the localization and release of intracellular substances.^[^ ^22^ ^]^ The potential of NEs translocation to the neutrophil surface to inhibit actin cytoskeleton rearrangement was further investigated, and phalloidin (green fluorescence) was utilized to label intracellular F‐actin (Figure 2h). After NE translocated to the nucleus within activated neutrophils, F‐actin was decomposed into G‐actin, which was expressed as a decrease in green fluorescence intensity (Figure S12d, Supporting Information). This breakdown of F‐actin represents the depolymerization of the actin cytoskeleton, a hallmark typically associated with cytoskeletal reorganization during NETs formation. The depolymerization of F‐actin facilitates cell membrane rupture and DNA release, processes that are intricately linked to NETs formation. However, when activated neutrophils were treated with FTP‐NPs, there was no significant decrease in the green fluorescence intensity of F‐actin, indicating that the stability of the actin cytoskeleton was maintained (Figure 2i).

To further verify whether FTP‐NPs would reduce the generation of NETs, we will detect the changes in different components of NETs after FTP‐NPs treatment. SYTOX Green dye could penetrate dead cells and bind to DNA. The cell nuclei in the FTP‐NPs group and NEi group had normal morphology and no DNA release, while the release of cellular DNA significantly increased in the control group (Figure 2j). Besides, MPO and CitH3, as classic markers of NETs, represent the formation and the dependent modification of histones in NETs. As shown in Figure 2k, the fluorescence signal of MPO in the activated neutrophils without treatment and treated by NFTP‐NPs was significantly enhanced, indicating that a large amount of MPO/DNA complexes were released. However, the fluorescence signal of MPO was significantly weakened, and the cell morphology remained intact, with no obvious release of NETs after FTP‐NPs and NEi treatments. The ELISA results of the MPO‐DNA test and CitH3 immunofluorescence staining/Western Blot confirmed that FTP‐NPs could effectively inhibit the formation of NETs (Figure 2l,m). The fluorescence signal of CitH3 was significantly enhanced, showing strong citrate modification of histone H3 in the activated neutrophils. The fluorescence signal of CitH3 weakened, indicating that FTP‐NPs and NEi inhibited the citrullination of histone H3 and further prevented the formation of NETs. NE‐fibril clusters inhibit the role of MPO and CitH3 in NETs formation by affecting skeleton rearrangement, thereby effectively alleviating the release of NETs.

### 2.8 NE‐Fibril Clusters Interfere with the Interaction between Tumor Cells and Activated Neutrophils In Vitro

**Figure 3:**
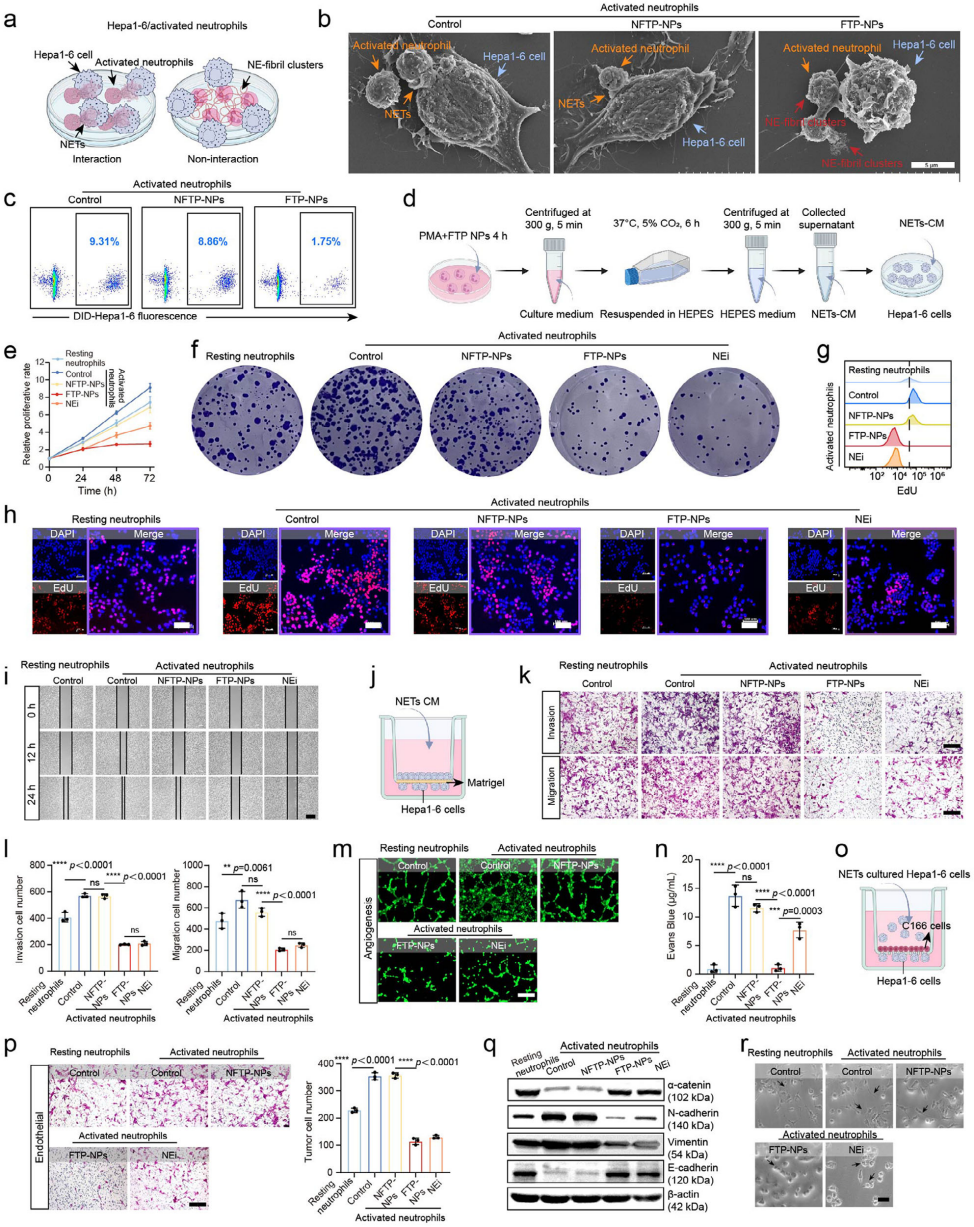
NE‐fibril clusters inhibit NETs formation of activated neutrophils to prevent Hepa1‐6 cells proliferation and metastasis. a) Schematic diagram of Hepa1‐6 cells adhesion to activated neutrophils. The scheme was created with BioRender.com. b) Representative SEM images of NETs and Hepa1‐6 cells. Scale bars, 5 µm. c) Representative Flow cytometry (FCM) profiles of DID‐Hepa1‐6 cells in neutrophils with various treatments. The concentration of NFTP‐NPs and FTP‐NPs was 50 µm. d) Flow chart of extracting culture medium from neutrophils treated with different treatments to act on tumor cells. The scheme was created with BioRender.com. e) Cell proliferation was detected by CCK assay. Experiments were repeated three times. f) Clone formation assay of Hepa1‐6 cells after being treated with culture medium extracted by neutrophils. g) Representative Flow cytometry (FCM) profiles images of EdU fluorescence in cells with various treatments. The concentration of NFTP‐NPs and FTP‐NPs was 50 µm. h) Representative fluorescence images of EdU fluorescence with various treatments. Scale bars, 100 µm. The concentration of NFTP‐NPs and FTP‐NPs was 50 µm. Experiments were repeated three times. i) Representative cell scratch images of Hepa1‐6 cells after different treatments. Scale bars, 400 µm. Experiments were repeated three times. j) Schematic diagram of transwell assays. The scheme was created with BioRender.com. k,l) Representative migration images and quantification of Hepa1‐6 cells stained by crystal purple via transwell assays. Scale bars, 200 µm. Experiments were repeated three times. Data are presented as mean ± SD, *n* = 3 independent experiments. m) The formation of tube structures by C166 cells in different media with or without NETs. n) Evans blue endothelial permeability assay. Data are presented as mean ± SD, *n* = 3 independent experiments. o) Schematic diagram of tumor cells penetrating endothelial cells. The scheme was created with BioRender.com. p) Representative images and quantification of Hepa1‐6 cells penetrating the endothelium by transwell assay, stained with crystal violet. Scale bars, 200 µm. Data are presented as mean ± SD, *n* = 3 independent experiments. q) Representative images of WB assay of four EMT‐related genes. r) Representative images of Hepa1‐6 cells by EMT‐related symptoms. Scale bars, 50 µm. Statistical significance was calculated by one‐way ANOVA, ****p < 0.0001, ***p < 0.001, n.s. means no significance.

The DNA, histones, and antimicrobial proteins in NETs provide an adhesion platform for tumor cells.^[^ ^23^ ^]^ Tumor cells bind to NETs through receptors to enhance their adhesion ability, thereby mediating the malignant biological behavior of tumor cell proliferation and metastasis. Therefore, it is necessary to study whether NE‐fibril clusters interfere with the interaction between activated neutrophils and tumor cells. A co‐culture model of activated neutrophils and Hepa1‐6 cells was constructed to study the interaction: activated neutrophils to the wall were pre‐adhered with lysine to produce NETs, and suspended Hepa1‐6 cells were added (**Figure** **3**a). We hypothesize that Hepa1‐6 cells adhere to the activated neutrophil surface because of binding to NETs. In contrast, Hepa1‐6 cells do not bind directly to neutrophils and remain in suspension, allowing them to be washed away in the absence of NETs formation. As shown in Figure 3b, NETs were observed to successfully mediate the adhesion of Hepa1‐6 cells in the activated neutrophil/Hepa1‐6 co‐culture system treatment with the NFTP‐NPs group and control group (without NPs treatment). In sharp contrast, a large number of NE‐fibril clusters were observed surrounding the surface of neutrophils, blocking their adhesion to Hepa1‐6 cells in the FTP‐NPs treatment group. Similarly, in the co‐incubation system of activated neutrophils and Hepa1‐6 cells, many DID‐labeled Hepa1‐6 cells (red) adhered to the activated neutrophils and were thus retained (Figure S13a, Supporting Information). In the flow cytometry analysis, the DID fluorescence in FTP‐NPs treatment group was significantly less than that in the NFTP‐NPs treatment group and the control group without treatments (Figure 3c; Figure S13b, Supporting Information).

### 2.9 Effect of Activated Neutrophils on Tumor Cell Proliferation and Metastasis Following FTP‐NPs Treatment

Freshly isolated neutrophils were incubated with PBS, PMA, PMA+NFTP‐NPs, PMA+FTP‐NPs, and PMA+NEi for 4 h to stimulate NETs formation^[^ ^24^ ^]^ (named as Resting neutrophils; Control group: activated neutrophils without treatment; NFTP‐NPs group: activated neutrophils + NFTP‐NPs; FTP‐NPs group: activated neutrophils + FTP‐NPs; NEi group: activated neutrophils + NE inhibitor Sivelestat). Neutrophils with culture medium were then centrifuged at 480 g for 10 minutes. The supernatant (NETs‐rich media) was then centrifuged at 18 000 × *g* for 15 min to form a pellet. The obtained pellet contained the mixture of chromatins and proteins, which was then resuspended in cell culture media to treat Hepa1‐6 cells (Figure 3d). As shown in Figure 3e, the cell proliferation experiment (CCK‐8 assay) showed that the proliferation of Hepa1‐6 cells was promoted in the control group (activated neutrophils) compared with the Resting neutrophils group. For the FTP‐NPs group, the proliferation rate of Hepa1‐6 cells was much lower than that of the Control group and NFTP‐NPs group, even that of the NEi group, indicating that FTP‐NPs significantly inhibited NETs formation of activated neutrophils. The results of the clone formation experiment, EdU staining experiments, and flow cytometry further supported this result (Figure 3f–h; Figure S14a,b, Supporting Information). These results indicate that NE‐fibril clusters inhibit the formation of NETs of neutrophils, thereby reducing the proliferation and colony formation of Hepa1‐6 cells, which exerts a significant antiproliferative effect.

On the other hand, we further evaluated the effect of NE‐fibril clusters on reducing NET formation in activated neutrophils to inhibit tumor metastasis. Tumor metastasis is a complex multi‐step process involving tumor cell migration ability, angiogenesis, increased vascular permeability, and vascular invasion of tumor cells. The scratch experiment was employed to investigate the migration ability of Hepa1‐6 cells in different culture media with or without NETs (Resting neutrophils; Control group: activated neutrophils without treatment; NFTP‐NPs group: activated neutrophils + NFTP‐NPs; FTP‐NPs group: activated neutrophils + FTP‐NPs; NEi group: activated neutrophils + NE inhibitor Sivelestat). After 24 h, the FTP‐NPs group showed the strongest inhibitory effect, such as the wound healing rate dropped significantly to 5.2% ± 1.8%, and the cell migration ability was significantly lower than the Control group and NFTP‐NPs group (Figure 3i; Figure S15a, Supporting Information). To more comprehensively evaluate the migration and invasion abilities of Hepa1‐6 cells in different culture media with or without NETs, we further conducted the transwell chamber experiment in a 3D context (Figure 3j). As shown in Figure 3k,l, the number of migrating cells in the FTP‐NPs group was significantly reduced and less than that in the other control groups. Then, the upper chamber was coated with Matrigel for the invasion experiments. The number of invasive cells in the FTP‐NPs group was significantly lower than that in the Control group and NFTP‐NPs group, showing the inhibitory effect on HCC cell invasion.

Besides, it is known that antimicrobial proteins in NETs, such as elastase and lysozyme, can degrade the extracellular matrix (ECM), a process that helps tumor cells break through the vascular wall and infiltrate deep into the tissue.^[^ ^25^ ^]^ To comprehensively evaluate the potential inhibition of FTP‐NPs on the distant organ metastasis of tumors, we conducted the following experiments. Matrigel tube formation assay showed that C166 endothelial cells in the culture medium derived from FTP‐NPs treated activated neutrophils (FTP‐NPs group) failed to form complete capillary‐like structures, and the number of tubular structures formed was significantly lower than that in the Control group and NFTP‐NPs group (Figure 3m). The permeability of blood vessels is also crucial for tumor cells to invade the blood circulation. When blood vessel permeability increases, tumor cells can more easily pass through the blood vessel wall, enter the bloodstream, and metastasize to distant organs. In vitro permeability experiments, we assessed the permeability of endothelial monolayers by detecting the absorbance of Evans blue dye. The results showed that the permeability of the endothelial monolayer in the FTP‐NPs group was significantly lower than that in the control group (Figure 3n). FTP‐NPs inhibit NETs formation in activated neutrophils, which effectively weakens the ability of tumor cells to enter the bloodstream by reducing the permeability of endothelial cells, thereby potentially slowing down the metastasis process of tumor cells.

Finally, we conducted transendothelial invasion experiments to further verify tumor cell invasion of blood vessels in different culture media with or without NETs (Figure 3o). As shown in Figure 3p, the number of Hepa1‐6 cells passing through the endothelial monolayer in the FTP‐NPs group was significantly lower than that in the Control group. Besides, the epithelial‐mesenchymal transition (EMT) promotes the metastasis of tumor cells from the primary tumor site to distant organs by regulating cell polarity, cell‐cell adhesion, and cell migration. The EMT process of Hepa1‐6 cells in different culture media with or without NETs was evaluated. Western blot and PCR analysis showed that the expressions of E‐cadherin and α‐catenin were significantly increased, while the expressions of N‐cadherin and Vimentin were significantly decreased in the FTP‐NPs treatment group (Figure 3q; Figure S15b,c, Supporting Information). As shown in Figure 3r, tumor cells usually reverted to an epithelial‐like morphology following the inhibition of EMT. This was characterized by increased cell density, more defined borders, and a predominantly polygonal or circular arrangement. NE‐fibril clusters on the surface of activated neutrophils significantly inhibit the formation of NETs to resist angiogenesis, decrease vascular permeability, and prevent vascular invasion of tumor cells, thereby minimizing the risk of tumor metastasis.

### 2.10 Biodistribution and Immune Regulatory Effects of FTP‐NPs in the Tumor Microenvironment In Vivo

**Figure 4:**
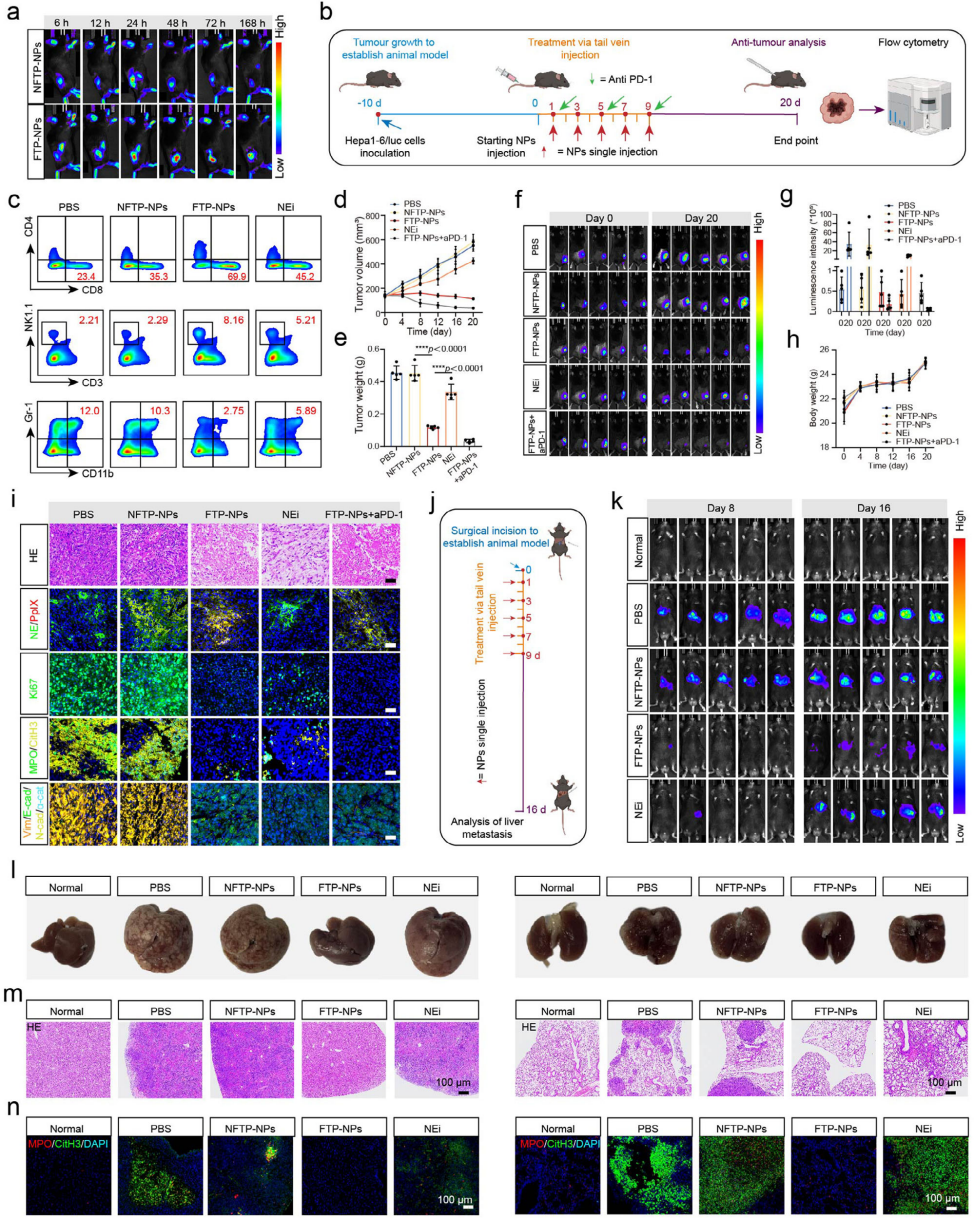
Antitumor activity and anti‐metastasis efficacy evaluation of FTP‐NPs in mice bearing Hepa1‐6/luc tumor. a) Time‐dependent in vivo fluorescence images of tumor tissues at 6, 12, 24, 48, 72, and 168 h post‐injection of NFTP‐NPs and FTP‐NPs. b) Schematic illustration of tumor inoculation and treatment protocol (*n* = 5 per group; the dose of NFTP‐NPs and FTP‐NPs was 300 µm per injection). The scheme was created with BioRender.com. c) Representative flow cytometric analysis image of CD8^+^ T cells (CD45^+^CD3^+^CD8^+^), NK cells (CD45^+^CD3^−^NK1.1^+^), and MDSC (CD45^+^CD11b^+^Gr‐1^+^). d) Average tumor growth curves of tumors (*n* = 5). e) Tumor weight at day 20 in five groups. Data are presented as mean ± SD, *n* = 5 independent experiments. Statistical significance was calculated by one‐way ANOVA, ****p < 0.0001. f) Luminescence images of tumor‐bearing mice at day 0 and day 20, respectively, through an in vivo Imaging System. g) Luminescence intensity of tumor sites at day 20. h) Body weight of mice treated in five groups. i) H&E staining and IF assay of tumors in the five groups. Scale bar, 40 µm. Experiments were repeated three times. j) Schematic illustration of the experimental approach for FTP‐NPs inhibiting liver and lung metastasis (*n* = 5 per group; the dose of NFTP and FTP‐NPs was 300 µM per injection). The scheme was created with BioRender.com. k) Luminescence images of liver and lung metastasis mice at day 8 and day 16, respectively, through an in vivo Imaging System. l) Representative photographs of liver and lung metastasis in Hepa1‐6/luc tumor‐bearing mice treated with PBS, NFTP, FTP‐NPs, and NEi for 16 days. m) H&E staining assay of liver and lung metastasis in the five groups. Scale bar, 100 µm. Experiments were repeated three times. n) IF staining assays of MPO and CitH3 in liver and lung tumor tissue in five groups. Scale bar, 100 µm. Experiments were repeated three times.

To further evaluate the tumor tissue accumulation of FTP‐NPs in vivo, we conducted in vivo biodistribution studies using a murine model. For biodistribution studies, mice bearing Hepa1‐6/luc tumors were treated by NFTP‐NPs and FTP‐NPs via tail vein injection and monitored over time using an In‐Vivo system (IVIS, **Figure** **4**a). After 12, 24, 48, and 168 h, tumors and major organs were collected for *ex vivo* fluorescence imaging (Figure S16a, Supporting Information). The fluorescence signal of the FTP‐NPs group was still detectable in tumor tissues after more than 3 days and lasted up to 7 days. In situ receptor‐mediated transformation of FTP‐NPs to NE‐fibril clusters in the tumor microenvironment may be responsible for the prolonged preservation of the fluorescence signal in FTP‐NPs‐treated mice, even after 7 days. In contrast, after 24 h, the fluorescence signal of the NFTP‐NPs began to decrease in normal organs, and by 72 h, it was essentially undetectable in major organs. The fluorescence signal of tumor tissue in the FTP‐NPs group at 48 h was 7.1‐fold higher than that in the NFTP‐NPs group (Figure S16b, Supporting Information).

Subsequently, we analyzed the regulatory effects of FTP‐NPs in the tumor environment. The inhibition of the NETs generation could significantly enhance the anti‐tumor immune response, mainly by regulating the functions and proportions of CD8⁺ T cells, NK cells, and MDSCs (Figure 4b). First, factors such as histones and NE released by activated neutrophils induce CD8⁺ T cell apoptosis or functional exhaustion, causing CD8⁺ T cells to lose their antitumor activity. In the analysis of effector T cells (CD45^+^CD3^+^CD8^+^), the infiltration proportion of CD8^+^ effector T cells in the FTP‐NPs group was significantly higher than that of other groups (Figure 4c; Figures S17 and S18, Supporting Information). Especially, the infiltration density of CD8^+^ T cells in the combined anti‐PDL1 antibody treatment group further increased.

The formation of NETs releases histones and DNA‐protein complexes, inhibits the function of NK cells, and interferes with their chemotaxis and activity through cytokines such as IL‐8 and IL‐6. Reducing the formation of NETs helps to restore the recruitment and killing effects of NK cells and enhance their ability to clear tumor cells. As shown in Figure 4c; Figures S19 and S20, Supporting Information in the analysis of NK cells (CD45^+^CD3^−^NK1.1^+^), the proportion of NK cells in the FTP‐NPs group was significantly increased. Plasma cytokine measurements revealed that IL‐6 and TNF‐α levels were significantly higher in the FTP‐NPs treatment group, indicating a pro‐inflammatory immune response that may contribute to enhanced NK cell activity. IL‐10 levels were also decreased, indicating a shift towards a more activated immune state (Figure S21, Supporting Information). On the basis of the immunoregulatory effect of FTP‐NPs, aPD‐1 can further enhance the role of NK cells in the tumor microenvironment, thereby improving the overall immune response. After combined use with aPD‐1, aPD‐1 may also indirectly promote the activation and tumor‐killing function of NK cells by relieving the immunosuppressive effect of T cells.

In addition, NETs can promote the secretion of factors such as G‐CSF, GM‐CSF, and IL‐6, induce the proliferation and enrichment of MDSCs, further inhibit the antitumor effects of CD8⁺ T cells and NK cells, and upregulate PD‐L1 expression, weakening the responsiveness of tumors to immunotherapy. Therefore, inhibiting NETs can not only reduce the accumulation of MDSCs and relieve the inhibition of antitumor immune cells, but also reduce the immunosuppressive signals in TIME and improve the effect of immunotherapy. As shown in Figure 4c and Figures S22 and S23 (Supporting Information), the proportion of MDSCs in the FTP‐NPs group was significantly reduced, showing significant differences compared with the PBS alone group and the NFTP‐NPs group. By releasing immunosuppressive signals, aPD‐1 may reduce the accumulation of MDSCs in the tumor microenvironment and further enhance the immune response. Treatment combined with FTP‐NPs not only inhibits the tumor immune escape mechanism but also significantly reduces the infiltration of immunosuppressive cells and breaks the immune tolerance state in the tumor microenvironment, thereby further improving the therapeutic effect. The above flow analysis results showed that FTP‐NPs significantly enhanced the tumor infiltration of T cells and NK cells, and significantly reduced the accumulation of MDSCs, forming a more favorable antitumor immune environment. The combined treatment of FTP‐NPs and aPD‐1 can effectively regulate the TIME and enhance the anti‐tumor immune response by lifting immune suppression and activating immune cells. It provides the basis for our subsequent tumor treatment.

### 2.11 The Antitumor Treatment Efficiency of FTP‐NPs In Vivo

We established a subcutaneous Hepa1‐6/luc HCC xenograft tumor model in C57BL/6J mice to investigate the effects of various treatment strategies on tumor growth and histopathological characteristics. Five groups of mice were administered PBS, NFTP‐NPs, FTP‐NPs, NEi, and a combination of FTP‐NPs and aPD‐1, respectively. During the treatment period, tumor volume and weight were monitored every four days. The FTP‐NPs‐treated group demonstrated significant efficacy in suppressing tumor growth compared to the NEi control group. Notably, in the FTP‐NPs + aPD‐1 group, the increase in tumor volume was markedly attenuated (Figure 4d,e; Figure S24, Supporting Information). Monitored by IVIS imaging technology, the bioluminescence signal of tumor tissue in the FTP‐NPs and FTP‐NPs + aPD‐1 treatment groups confirmed the significant anti‐tumor effect on the 20th day after the start of treatment (Figure 4f,g). These results indicate that FTP‐NPs have a strong effect in inhibiting tumor growth. And aPD‐1 enhances the immune system's ability to recognize and eliminate tumor cells by relieving tumor immune evasion.

There was no significant change in body weight during the treatment process (Figure 4h). To further explore its safety, after five consecutive injections of NFTP‐NPs and FTP‐NPs, the subcutaneous tumor model mice were euthanized, and the blood and plasma of the mice were obtained. The liver function, platelets, creatinine, and blood cell count of the mice were tested (Figure S25, Supporting Information). FTP‐NPs were determined to be nontoxic, indicating that FTP‐NPs had good safety for mice at this dose. Furthermore, a hemolysis assay was performed to evaluate the potential hemolytic activity of FTP‐NPs on mouse red blood cells (RBCs). The results demonstrated minimal hemolysis, with the maximum observed hemolysis rate being approximately 0.9% at the highest tested concentration of 1000 µm (Figure S26, Supporting Information). This suggests that FTP‐NPs are well tolerated and exhibit negligible hemolytic activity, further supporting their safety profile for in vivo applications.

In addition, tumors and major organs were collected for biochemical and morphological evaluations to better understand the anti‐tumor mechanism and safety in vivo. No obvious abnormalities were found in the tissue sections of the major organs (Figure S27, Supporting Information). There was a strong correlation between the degree of tumor cell death and fluorescence intensity; the strong fluorescence signal of PpIX in these tumor areas colocalized well with the NE protein signal in the FTP‐NPs‐treated group (Figure 4i; Figure S28, Supporting Information). No obvious cell death was found in the tumor sections of mice treated with PBS and NFTP‐NPs.

Besides, the immunofluorescence results of tumor tissue showed that the proportion of Ki67‐positive cells was significantly reduced in the FTP‐NPs group, indicating that cell proliferation was inhibited. Immunofluorescence staining analysis confirmed that the expression of NETs markers MPO and CitH3 was significantly down‐regulated in the FTP‐NPs and combination treatment groups. The expression of E‐cadherin and α‐catenin was significantly enhanced, while the expression of N‐cadherin and Vimentin decreased, indicating that the EMT process was inhibited during FTP‐NPs treatment. FTP‐NPs treatment has the potential to reshape the tumor immune microenvironment by inhibiting NETs generation, thereby promoting immune cell infiltration and enhancing the anti‐tumor activity of T cells and NK cells. Additionally, it may further improve therapeutic efficacy by inhibiting the EMT process and reducing tumor cell invasiveness.

### 2.12 The Tumor Metastasis Suppression of FTP‐NPs In Vivo

We evaluated the effects of NFTP‐NPs, FTP‐NPs, and NEi on liver and lung metastasis using the Hepa1‐6/luc mouse model (Figure 4j; Figure S29a, Supporting Information). After 16 days, the tumor metastasis of mice in each group was observed by bioluminescence imaging (Figure 4k; Figure S29b, Supporting Information). The normal group (non‐tumor‐bearing mice) had normal mouse organs. The mice bearing Hepa1‐6/luc tumor in the PBS, NFTP‐NPs, and NEi groups showed obvious metastases in the liver and lungs. In sharp contrast, the bioluminescent signals and the number of metastatic foci in the liver and lungs were weakened considerably and significantly lower in the FTP‐NPs group (Figure 4l,m; Figure S29c,d, Supporting Information). In addition, immunohistochemical staining further demonstrated the expression levels of NETs markers MPO and CitH3. The expression of MPO and CitH3 was significantly attenuated in the FTP‐NPs group, suggesting that the treatment might slow down the metastatic process by inhibiting the formation of NETs (Figure 4n). Although NEi also showed potential in some aspects, its effect was relatively weak compared with that of FTP‐NPs. This finding supports that NE‐fibril clusters derived from FTP‐NPs have significant effects in inhibiting tumor metastasis via specifically regulating the subcellular location and activation of NEs to inhibit NETs generation of neutrophils, which provides a new therapeutic direction for inhibiting tumor metastasis.

### 2.13 Single‐Cell Transcriptomic Analysis of the Cellular Composition and Immune Microenvironment Remodeling in HCC Following FTP‐NPs Treatment

**Figure 5:**
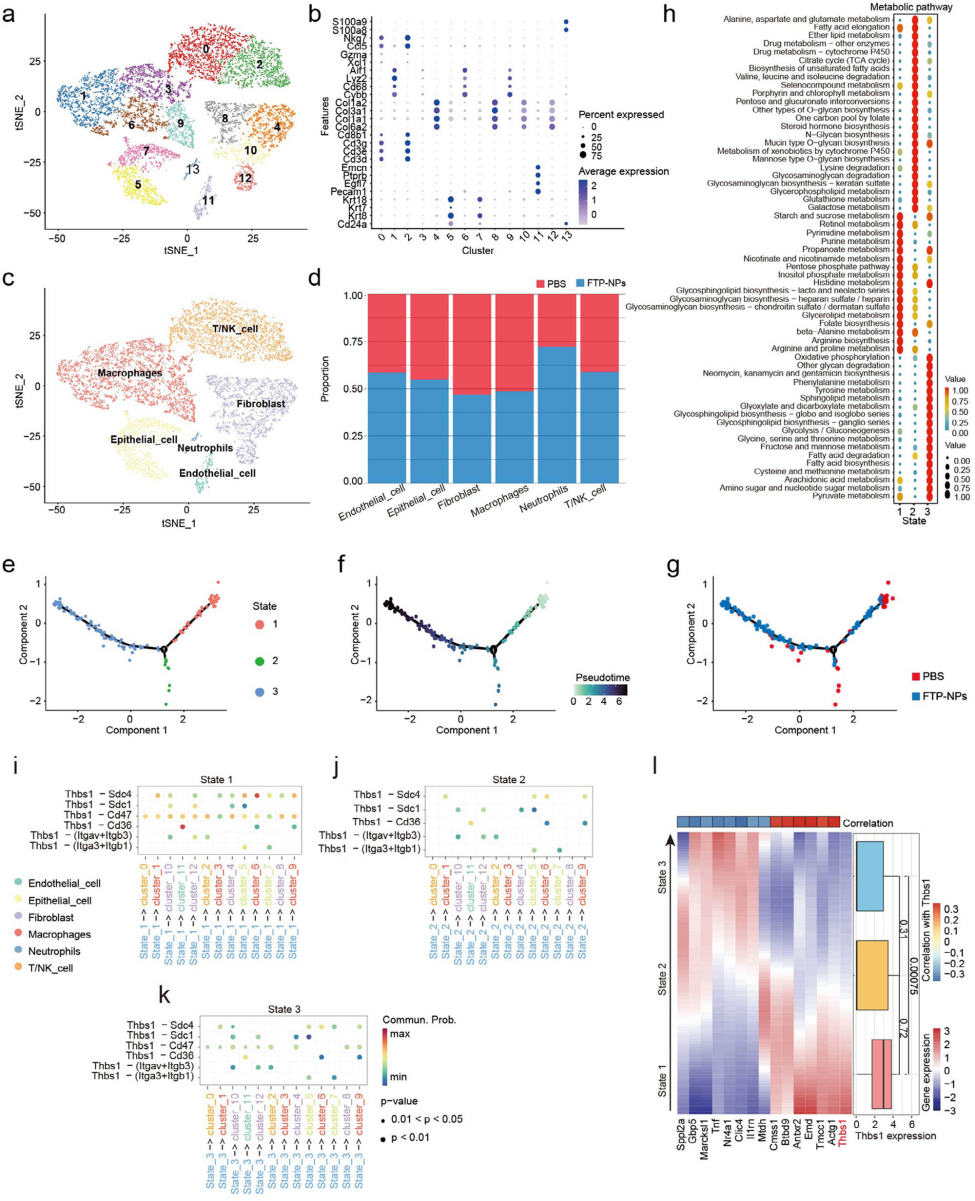
Single‐cell analysis reveals that Thbs1 mediates neutrophil interactions to reshape the tumor immune microenvironment. a) The cells from PBS and FTP‐NPs samples were clustered into 14 distinct cell clusters. b) The gene markers of the 14 cell clusters are shown, with the *x*‐axis representing the cell clusters and the *y*‐axis representing the gene markers. The deep blue color indicates high gene expression levels, while the size of the dots represents the number of cells expressing the gene. c) The cells from the PBS and FTP‐NPs samples were primarily identified as six different cell types. d) The proportion of cell types in PBS and FTP‐NPs samples for the six identified cell types, where blue represents the FTP‐NPs sample and red represents the PBS sample. e) Neutrophils can be classified into three distinct states. f) The pseudotime trajectory of neutrophils. g) The distribution of neutrophils in the pseudotime trajectory for PBS and FTP‐NPs samples, where red represents PBS samples and blue represents FTP‐NPs samples. h) The metabolic pathway activity scores of neutrophils in three states, with the *x*‐axis representing neutrophils in the three states and the *y*‐axis representing KEGG metabolic pathways. Red represents high activity scores, while blue represents low activity scores. i–k) Intercellular communication regulation of neutrophils in State 1, State 2, and State 3 by other cell clusters in the TIME. The *x*‐axis represents the intercellular communication pairs, and the *y*‐axis represents the receptor–ligand pairs. l) The expression level changes of Thbs1 in neutrophils in three states, and genes highly correlated with Thbs1 expression. In the heatmap, the *x*‐axis represents genes highly correlated with Thbs1 expression, and the *y*‐axis represents the pseudotemporal distribution of neutrophils in the three states. In the boxplot, the *x*‐axis represents the gene expression level of Thbs1, and the *y*‐axis represents the neutrophils in the three states, *p*‐values were calculated with the Wilcoxon rank sum test.

We performed single‐cell RNA sequencing (scRNA‐seq) analysis on tumor samples from PBS and FTP‐NPs‐treated groups. This allowed us to precisely characterize the cellular composition, gene expression profiles, and functional interactions among distinct cell populations within the tumor immune microenvironment. After stringent quality control, we retained 12833 high‐quality single cells for subsequent analysis, followed by the identification of 1020 highly variable genes (Figure S30a, Supporting Information). Principal component analysis (PCA) and unsupervised clustering separated the cells into six major clusters, which were further divided into 14 subclusters at a higher resolution (**Figure** **5**a; Figure S30b–d, Supporting Information). Notably, clusters 0 and 2 showed high expression of Cd3d, Cd3e, Cd3g, and Nkg7, while clusters 4, 8, 10, and 12 expressed high levels of Col6a2, Col1a1, Col3a1, and Col1a2. Cluster 13 was characterized by high expression of S100a8, S100a9, and Cd24a (Figure 5b).^[^ ^26^, ^27^, ^28^ ^]^ A comparative analysis of cluster proportions between PBS and FTP‐NPs groups revealed significant differences, suggesting distinct immune cell recruitment patterns following FTP‐NPs treatment (Figure S30e, Supporting Information). Specifically, clusters 2, 9, and 13 exhibited notably higher proportions in the FTP‐NPs group, whereas clusters 1 and 4 predominated in the PBS group. These results suggest that FTP‐NPs treatment may significantly modulate the recruitment and functional status of distinct cell clusters within the tumormicroenvironment.

### 2.14 Dynamic Functional Transition and Metabolic Reprogramming of Neutrophil Subsets Following FTP‐NPs Treatment

To better understand the biological roles and mechanisms of the identified cell clusters, we annotated the 13 clusters using SingleR and Cell Taxonomy to define their specific identities within the HCC immune microenvironment (Figure S30f,g, Supporting Information). These clusters represented six main cell types: macrophages (clusters 1, 3, 6, 9), T/NK cells (clusters 0, 2), epithelial cells (clusters 5, 7), endothelial cells (cluster 11), fibroblasts (clusters 4, 8, 10, 12), and neutrophils (cluster 13, Figure 5c).^[^ ^28^ ^]^ Malignant epithelial cells were further confirmed using the inferCNV algorithm, showing significantly higher copy number variation (CNV) scores compared to endothelial cells, thus indicating their malignant status (Figure S30h, Supporting Information). We quantified the proportions of six distinct cell types in PBS and FTP‐NPs‐treated samples. Only neutrophils showed a significantly higher proportion in the FTP‐NPs‐treated group, while the proportions of other cell types did not differ notably between the two groups (Figure 5d). These findings suggest that neutrophils may be the cell type most prominently affected by FTP‐NPs treatment.

To characterize dynamic functional transitions of neutrophils between PBS and FTP‐NPs conditions, we performed pseudotime analysis using Monocle2 on 141 neutrophils, identifying three distinct pseudotemporal states. State 1 represented the initial phase, while state 3 corresponded to the terminal differentiation state (Figure 5e,f). Moreover, neutrophils from PBS samples predominantly occupied state 1, whereas FTP‐NPs‐treated neutrophils were enriched in state 3 (Figure 5g). To explore the functional heterogeneity of neutrophils across the three pseudotemporal states, we identified state‐specific highly expressed genes. S100a8 and S100a9 were enriched in state 1, Slc7a2 and Ece1 in state 2, and Tnf and Ccl3 in state 3, suggesting distinct transcriptional programs (Figure S31a–c, Supporting Information). Metabolic activity analysis revealed that state 3 neutrophils exhibited elevated activity in multiple KEGG pathways, including oxidative phosphorylation, steroid hormone biosynthesis, and tyrosine metabolism (Figure 5h), indicative of metabolic reprogramming and enhanced immune activation. This dynamic transition indicates that neutrophils progressively lose their NETs‐forming capacity and adopt a metabolically active, proinflammatory phenotype. FTP‐NPs treatment may reshape the tumor immune microenvironment by promoting immune activation, thereby potentially enhancing anti‐tumor immunity and modulating disease progression in HCC.

### 2.15 Neutrophil Functional Reprogramming and Inflammatory Pathway Activation During FTP‐NPs Treatment

To better characterize neutrophil functional reprogramming, we performed differential gene expression analysis between neutrophils in pseudotime states 1 and 3 during the transition from PBS to FTP‐NPs treatment. We compared neutrophils in states 1 and 3, as PBS‐derived neutrophils were predominantly in state 1, while FTP‐derived neutrophils were enriched in state 3. We identified 48 differentially expressed genes, among which 23 were significantly upregulated in state 3 neutrophils. GO and KEGG enrichment analyses revealed that state 3 neutrophils exhibited increased expression of genes related to leukocyte migration, myeloid leukocyte migration, and cell chemotaxis (Figure S32a, Supporting Information). These cells also showed enhanced cytokine receptor binding and cytokine activity, indicating that they play a key role in immune signaling and inflammatory responses. In contrast, molecular functions related to G protein‐coupled receptor binding and S100 protein binding were significantly downregulated. In addition to enhanced Tnf and chemokine signaling, state 3 neutrophils showed significant downregulation of IL‐17 signaling (Figure S32b, Supporting Information). IL‐17 is known to promote NETs formation and contribute to chronic inflammation, which are key features of the pro‐inflammatory phenotype.^[^ ^29^ ^]^ The suppression of IL‐17 signaling suggests a functional reprogramming of neutrophils, shifting them away from IL‐17‐mediated immune responses. Consequently, state 3 neutrophils adopt a non‐canonical immune activation phenotype, characterized by reduced NETosis and enhanced cytokine‐driven inflammation. This shift indicates that neutrophils in state 3 may be less involved in NETs formation but more engaged in promoting inflammatory responses through cytokine signaling.

Further comparison between neutrophils in states 2 and 3 identified 15 differentially expressed genes enriched in granulocyte and neutrophil migration (Figure S33a, Supporting Information). Immune‐related pathways, including NF‐κB and TNF signaling, were activated in state 3 neutrophils compared with state 2 (Figure S33b, Supporting Information). Comparing neutrophils from states 1 and 2, 37 differentially expressed genes were identified, demonstrating suppression of receptor‐related molecular functions (G protein‐coupled receptor and S100 protein binding) but concurrent activation of NF‐κB and TNF signaling pathways in state 2 (Figure S33c,d, Supporting Information). These findings suggest that state 2 represents a transitional neutrophil state characterized by both immune pathway activation and receptor function suppression. In summary, these analyses indicate that FTP‐NPs treatment induces significant functional reprogramming of neutrophils. Particularly, sustained activation of inflammatory pathways (NF‐κB and TNF signaling) and suppression of IL‐17 signaling may promote chronic inflammatory responses and immune evasion, ultimately reshaping the TIME. These observations provide valuable insights into the diverse immune‐regulatory roles of neutrophils following FTP‐NPs treatment in HCC.

### 2.16 Thbs1‐Mediated Neutrophil Interactions Reshape the Tumor Immune Microenvironment

To characterize the dynamic interactions between neutrophils and other cell types across pseudotime states, we performed cell–cell communication analysis using the CellChat R package. In state 1, neutrophils showed extensive interactions with endothelial cells and malignant epithelial cells through Thbs1‐related interactions, including Thbs1‐(Itga3+Itgb1), Thbs1‐(Itgav+Itgb3), and Thbs1‐Cd36 axes, all of which contribute to cell adhesion, immune suppression, and stromal remodeling (Figure 5i). In states 2 and 3, neutrophils exhibited significantly reduced Thbs1‐mediated interactions with other cell types. While Thbs1‐related communication remained detectable in state 2, its reduced intensity suggests a transitional phase where neutrophils began to lose immunosuppressive interactions (Figure 5j). By state 3, neutrophils showed markedly diminished Thbs1‐mediated signaling, indicating a shift away from Thbs1‐driven immunoregulatory programs (Figure 5k). We next investigated ligand‐receptor interactions mediating neutrophil communication with macrophages and other cell populations (Figure S34a–c, Supporting Information). In state 1, neutrophils engaged macrophages predominantly via Thbs1‐Cd36, Thbs1‐Cd47, and Thbs1‐Sdc4 axes, which are closely associated with immune suppression and macrophage polarization (Figure S34a, Supporting Information). In state 2, neutrophils retained certain immunomodulatory interactions (e.g., Il1b‐(Il1r1+Il1rap) axis and Osm‐(Osmr+Il6st) axis) while increasingly activating fibroblasts through chemokine signaling (e.g., Ccl4‐Ccr5), suggesting enhanced stromal remodeling (Figure S34b, Supporting Information). By state 3, Thbs1‐mediated signaling was markedly reduced, whereas interactions involving proinflammatory cytokines and adhesion molecules, such as Ccl3‐Ccr5 and Ccl4‐Ccr5 axes, became predominant (Figure S34c, Supporting Information). This shift highlights a functional reprogramming of neutrophils toward a phenotype characterized by immune activation and proinflammatory activity.

As a matrix protein involved in inflammation and immunosuppressive signaling pathways, Thbs1 is strongly associated with poor prognosis in HCC.^[^ ^30^, ^31^, ^32^ ^]^ Further analysis revealed that Thbs1 expression progressively decreased from state 1 to state 3 (Figure 5l and *p*‐value < 0.050). This reduction may mark a functional transition from immunosuppressive to proinflammatory neutrophil states, contributing to enhanced immune activation in the tumor microenvironment. Additionally, we identified 14 genes significantly correlated with Thbs1 expression (|R| > 0.3, *p‐*value < 0.050). Most of these genes were involved in inflammation, cell migration, and cell adhesion (Figure S34d, Supporting Information). Notably, key genes such as Sppl2a, Mtdh, and Cmss1 are known regulators of neutrophil‐mediated immune responses, with Sppl2a being involved in cytokine processing,^[^ ^33^ ^]^ Mtdh in immune signaling,^[^ ^34^ ^]^ and Cmss1 in cell migration.^[^ ^35^ ^]^ Therefore, the downregulation of Thbs1 and its associated gene network may reflect a shift toward neutrophil functional activation, potentially enhancing inflammatory signaling and intercellular communication within the tumor microenvironment. These findings suggest that Thbs1 downregulation is linked to neutrophil reprogramming and enhanced immune activation, potentially reshaping the tumor immune microenvironment in HCC.

### 2.17 Association between Neutrophil Functional Transitions and Patient Prognosis in Hepatocellular Carcinoma

We analyzed neutrophil states (state 1 and state 3) in PBS‐ and FTP‐NPs‐treated samples, examining their association with HCC patient prognosis. PBS samples predominantly contained neutrophils in state 1, whereas FTP‐NPs‐treated samples were enriched with neutrophils in state 3. Further survival analysis revealed that a higher proportion of state 1 neutrophils was significantly correlated with poorer clinical outcomes, including shorter overall survival (OS), progression‐free interval (PFI), disease‐free interval (DFI), and disease‐specific survival (DSS), indicating that state 1 neutrophils may exacerbate tumor progression and immune evasion in HCC (log‐rank test, *p‐*value < 0.05 for all comparisons; Figure S34e–h, Supporting Information). These findings indicate that state 1 neutrophils may contribute to immune suppression and tumor progression in HCC.

Conversely, FTP‐NPs samples exhibited improved prognosis associated with an increased proportion of state 3 neutrophils, demonstrated by significantly prolonged OS, DSS, PFI, and DFI (log‐rank test, *p‐*value < 0.05; Figure S34i–l, Supporting Information). These results suggest that state 3 neutrophils possess enhanced anti‐tumor immune functions, potentially limiting immune evasion and improving clinical outcomes. Thus, the clinical efficacy of FTP‐NPs treatment may be partly attributed to promoting the transition of neutrophils from state 1 to state 3. Our findings support the hypothesis that FTP‐NPs treatment reshapes the TIME by reducing state 1 (immunosuppressive) neutrophils and promoting their transition to state 3 (immune‐activated). Targeting neutrophil functional states, particularly promoting state 3 polarization, presents a promising strategy to enhance therapeutic effectiveness and survival outcomes for patients with HCC. Future studies should further explore therapeutic interventions to modulate neutrophil functional states for personalized HCC immunotherapy.

### 2.18 FTP‐NPs Induce Cell Cycle Arrest and Inhibit TLR/NF‐κB Signaling in Tumor Cells

**Figure 6:**
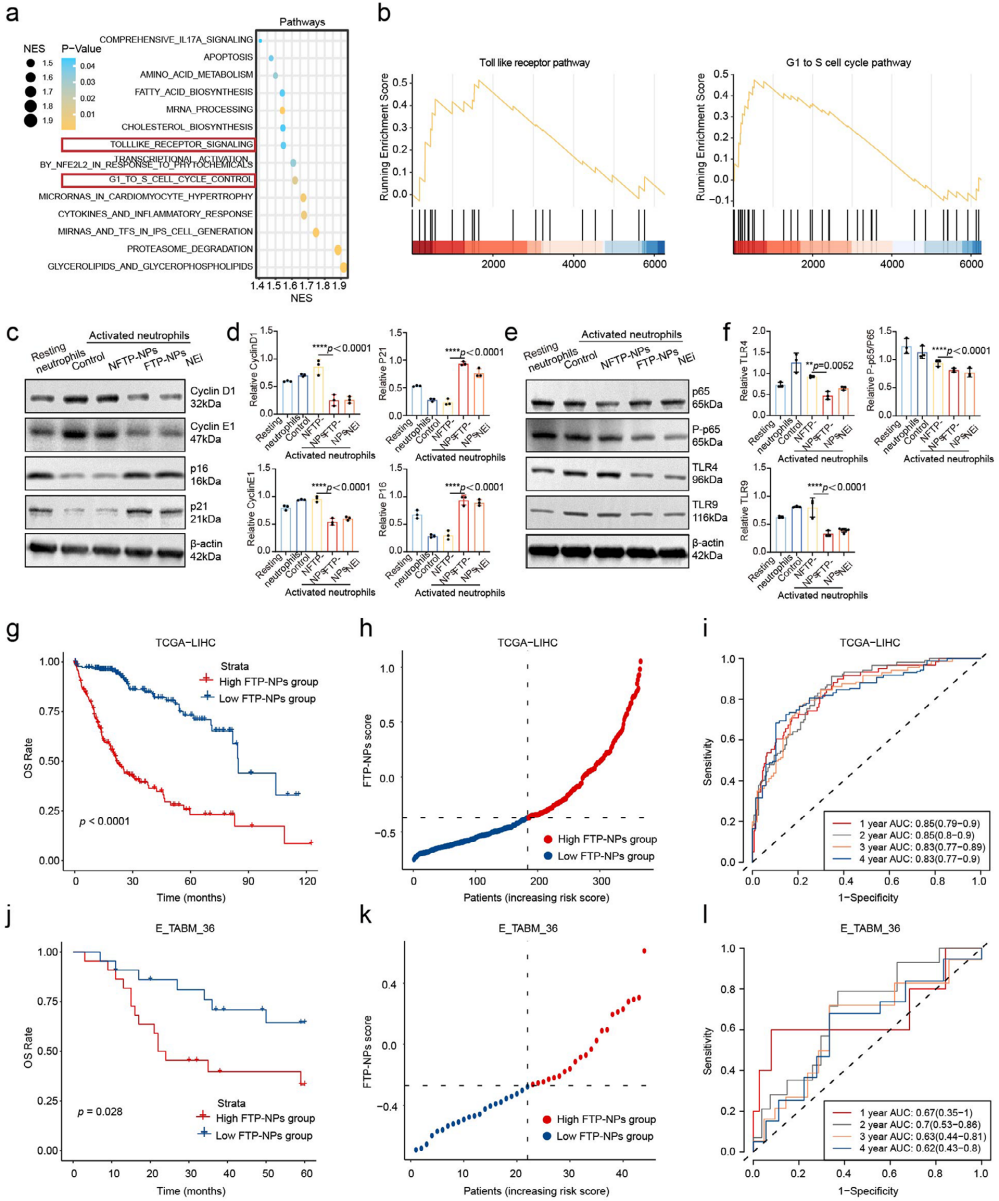
The biological process changes between tumor cells in the FTP‐NPs and PBS groups. a) KEGG pathway enrichment analysis of differentially expressed genes. The node color represents the significance of the enrichment results, and the node size represents the number of enriched genes. The *x*‐axis is −log10 (*p*‐value), and the *y*‐axis represents the KEGG pathway information. b) Gene set enrichment analysis (GSEA) of WikiPathways gene sets between tumor cells in PBS and FTP‐NPs groups. c,d) Representative images and quantification of WB assay for cell cycle‐related genes. e,f) Representative images and quantification of WB assay for Toll‐like receptor‐related genes. g) Comparison of OS between the low FTP‐NPs and high FTP‐NPs groups in the TCGA‐LIHC cohort, the *p*‐values were calculated with the log‐rank test. h) The distribution of the FTP‐NPs score in the TCGA LIHC cohort. i) ROC curve of the FTP‐NPs scoring model for predicting the OS of patients from the TCGA‐LIHC cohort. j) Comparison of OS between the low FTP‐NPs and high FTP‐NPs groups in the E‐TABM‐36 cohort, the *p*‐values were calculated with the log‐rank test. k) The distribution of the FTP‐NPs score in the E‐TABM‐36 cohort. l) ROC curve of the FTP scoring model for predicting the OS of patients from the E‐TABM‐36 cohort.

To explore biological changes in tumor cells following FTP‐NPs treatment, we analyzed single‐cell transcriptomic profiles of 726 tumor cells (PBS: 324 cells; FTP‐NPs: 400 cells). Differential gene expression analysis identified 119 significantly altered genes in FTP‐NPs‐treated cells, including 64 upregulated and 55 downregulated genes compared to PBS (Figure S35a, Supporting Information). Functional enrichment analysis revealed these genes were significantly associated with biological processes such as rRNA metabolism, reactive oxygen species (ROS) metabolism, and rRNA processing (Figure S35b, Supporting Information). Additionally, altered genes were enriched in cellular components, including small nuclear ribonucleoprotein complexes and preribosomes (Figure S35c, Supporting Information), as well as molecular functions linked to the cell cycle and metastasis, notably tRNA methyltransferase activity and snoRNA binding (**Figure** **6**a,b; Figure S35d, Supporting Information). These findings imply that FTP‐NPs affect tumor cell proliferation, metastatic capability, and immune response regulation, potentially contributing to their antitumor effects.

To validate these results, Western blot analysis confirmed significant downregulation of Cyclin D1 and Cyclin E1, along with upregulation of cell cycle inhibitors P21 and P16 after FTP‐NPs treatment (Figure 6c,d). Cyclin D1 and Cyclin E1 facilitate the G1/S phase transition essential for cell proliferation; their suppression, together with increased expression of P21 and P16, suggests FTP‐NPs induce G1/S cell cycle arrest, thereby inhibiting tumor cell growth. We further investigated changes in Toll‐like receptor (TLR) signaling and downstream NF‐κB pathway activation, both critical in immune response regulation and metastasis. FTP‐NPs treatment markedly decreased the expression of TLR4, TLR9, and NF‐κB (Figure 6e,f), which are involved in tumor metastasis and immune evasion through NF‐κB‐mediated cytokine release. Their downregulation suggests reduced inflammatory signaling, thereby inhibiting tumor invasion and metastasis. Altogether, our results indicate that FTP‐NPs exert anti‐tumor activity by inducing cell cycle arrest and inhibiting TLR/NF‐κB‐mediated inflammation. By modulating these key signaling pathways, FTP‐NPs may effectively suppress tumor growth, metastasis, and immune evasion, providing valuable insights for future clinical applications in HCC therapy.

### 2.19 Construction and Validation of the FTP‐NPs Risk Score

To evaluate the prognostic significance of tumor‐derived differentially expressed genes following FTP‐NPs treatment in HCC, we first mapped the 119 differentially expressed genes from single‐cell RNA sequencing onto the human genome, ultimately identifying 95 HCC‐associated genes used to construct the FTP‐NPs score. Survival analysis in the TCGA‐LIHC cohort demonstrated that patients in the high‐risk group had significantly worse outcomes compared to those in the low‐risk group (log‐rank test, *p‐*value < 0.001, Figure 6g). Moreover, an increasing proportion of high‐risk patients corresponded with elevated FTP‐NPs scores (Figure 6h). Time‐dependent ROC analysis revealed robust predictive performance, with AUC values of 0.85, 0.85, 0.83, and 0.83 at 1, 2, 3, and 4 years, respectively (Figure 6i). Similar results were validated in the independent E‐TABM‐36 cohort, where low‐risk patients exhibited better survival outcomes (log‐rank test, *p‐value* = 0.028, Figure 6j), and an increased FTP‐NPs score correlated with elevated mortality risk (Figure 6k). ROC analysis further confirmed these results, showing AUC values of 0.67, 0.70, 0.63, and 0.62 at 1, 2, 3, and 4 years, respectively (Figure 6l).

To further investigate the association between the FTP‐NPs score and the immune microenvironment in HCC, single‐sample gene set enrichment analysis (ssGSEA) was performed to evaluate the activity scores of 17 immune‐related pathways obtained from the ImmPort database. Pearson's correlation analysis indicated higher immune cell interactions in patients with lower FTP‐NPs scores (Figure S36a, Supporting Information). In addition, immune infiltration analysis using multiple computational methods revealed significant differences in immune cell abundance between high FTP‐NPs and low FTP‐NPs groups. Consistently, Pearson's correlation demonstrated a negative correlation between FTP‐NPs score and the infiltration levels of naive B cells, NK cells, and CD8^+^ and CD4^+^ T cells (Figure S36b,e, Supporting Information). These findings collectively suggest that low FTP‐NP patients may be more likely to benefit from immunotherapy.

To identify key gene modules associated with FTP‐NPs treatment, we performed weighted gene co‐expression network analysis (WGCNA) using the TCGA‐LIHC dataset to construct a gene co‐expression network for HCC patients. WGCNA results revealed that genes associated with the low FTP‐NPs group were predominantly enriched in immune‐related pathways, whereas genes associated with the high FTP‐NPs group were enriched in cancer progression‐related pathways (Figure S37a–d, Table S2, Supporting Information). Subsequently, to validate these findings, enrichment analysis was conducted using differentially expressed genes (DEGs) between high and low FTP‐NPs groups. The results were consistent with the WGCNA findings. Specifically, DEGs from the low FTP‐NPs group were significantly enriched in pathways related to cell cycle regulation and DNA damage repair, including hallmark mitotic spindle, hallmark g2m checkpoint, hallmark e2f targets, Reactome Fanconi anemia pathway, Reactome homology directed repair, Reactome base excision repair, kegg base excision repair, kegg cell cycle, kegg maturity onset diabetes of the young, and kegg DNA replication. Conversely, DEGs from the high FTP‐NPs group were enriched in pathways closely associated with inflammation, tumor progression, and immune suppression, including the hallmark p53 pathway, the hallmark TNFA signaling via NFκB, the hallmark KRAS signaling up, the KEGG JAK STAT signaling pathway, and the Reactome neutrophil degranulation (Figure S37e–g, Supporting Information). These findings provide comprehensive biological insights into the prognostic and therapeutic implications of FTP‐NPs treatment and highlight potential biomarkers and targets for personalized immunotherapy in HCC.

### 2.20 Construction and Calibration of an Integrated Nomogram

We observed a significant association among the NETs group, the FTP‐NPs group, and the overall survival (OS) status (Figure S38a, Supporting Information). To further enhance prognostic accuracy and facilitate personalized prediction in clinical practice, we constructed a prognostic nomogram based on NETs score, FTP‐NPs score, and key clinical parameters using the TCGA‐LIHC cohort (Figure S38b, Supporting Information). This integrated nomogram allows individualized estimation of survival probabilities at 1‐, 3‐, and 5‐year intervals. Calibration plots demonstrated strong agreement between predicted survival and actual observed outcomes, indicating high predictive reliability (Figure S38c, Supporting Information). Additionally, the predictive performance of the nomogram was robust, achieving a concordance index (C‐index) of 0.77. This comprehensive prognostic model not only underscores the clinical relevance of NETs and FTP‐NPs scores but also provides a valuable tool for precision risk stratification and therapeutic decision‐making in patients with HCC.

## 3 Conclusion

In this study, we developed a novel and targeted therapeutic approach using a peptidic nanomaterial, FTP, which specifically targets NE proteins on the surface of activated neutrophils within the tumor immune microenvironment (TIME). By leveraging the unique ability of FTP‐NPs to undergo in situ fibrillar transformation, we successfully induced the formation of NE‐fibril clusters that exerted a multifaceted inhibitory effect on NETs formation and tumor metastasis.^[^ ^36^ ^]^ This strategy operates through two complementary pathways: first, by disrupting the balance between NE and AAT on the neutrophil surface, FTP‐NPs promote the externalization and inactivation of NE, preventing its role in chromatin decondensation; second, by blocking NE's translocation to the nucleus, which further inhibits NETs formation.^[^ ^37^ ^]^ The formation of NE‐fibril clusters not only prevents the generation of NETs but also acts as a physical barrier, impeding the adhesion of these NETs to tumor cells, thereby disrupting the metastatic cascade.^[^ ^38^ ^]^ This dual mechanism of action is a significant advancement over conventional strategies, offering a more targeted and localized approach to modulating NE activity. Importantly, the NE‐fibril clusters formed by FTP‐NPs demonstrate high specificity for activated neutrophils in the TIME, which minimizes off‐target effects and reduces systemic toxicity, a major limitation of current NE inhibitors. Our comprehensive analysis, including in vitro and in vivo studies as well as scRNA‐seq, confirmed that FTP‐NPs significantly reduced NETs formation, diminished tumor metastasis, and enhanced anti‐tumor immune responses. These findings suggest that FTP‐NPs could serve as a potent therapeutic agent in cancer treatment by targeting the pivotal role of NETs in tumor progression and metastasis. Furthermore, the ability of FTP‐NPs to localize their action to the TIME presents a substantial improvement over existing treatment options, which are often associated with systemic side effects. Compared to traditional NE inhibitors, which are typically systemic and may not provide precise control over NE localization and activity, the FTP‐NPs approach offers a more effective and targeted strategy. The selective binding and fibrillar transformation of NE on neutrophil surfaces ensure that the therapeutic action is concentrated in the tumor site, minimizing potential toxicities in healthy tissues and offering a more controlled means of regulating NETs in the context of cancer metastasis. This innovative strategy not only provides new insight into the role of NE and NETs in tumor progression but also opens the door to the development of a novel class of targeted therapies for NETs‐mediated tumor metastasis. Beyond their role as a single‐agent therapy, FTP‐NPs also hold promise for future clinical applications. Their capacity to modulate neutrophil function and suppress NETs formation positions them as an attractive candidate for combination with other therapeutic modalities. For example, pairing FTP‐NPs with immune checkpoint inhibitors could synergistically augment anti‐tumor immunity, while their use alongside chemotherapy or radiotherapy may reduce metastatic spread and overcome treatment resistance. Such combinatorial strategies could expand the therapeutic spectrum of FTP‐NPs and maximize their clinical impact. In summary, our findings present a promising and innovative strategy for regulating NETs‐mediated tumor metastasis through targeted NE‐fibril cluster formation. This work lays the foundation for developing new therapeutic strategies that harness the power of nanotechnology to precisely modulate immune cell behavior within the TIME, offering a novel avenue for combating cancer metastasis.

In future studies, we aim to explore the combination of FTP‐NPs with other cancer treatments, such as immunotherapy or chemotherapy, to further enhance therapeutic outcomes. Additionally, further optimization of the nanoparticle design and in‐depth investigation of the mechanism by which NE‐fibril clusters modulate the time will be key to maximizing the clinical potential of this approach. Despite the encouraging therapeutic outcomes demonstrated in our study, several key challenges must be addressed before FTP‐NPs can be translated into clinical applications. First, the heterogeneity of the tumor immune microenvironment across different cancer types and patient populations may result in variable therapeutic responses, necessitating careful patient stratification and personalized treatment strategies. Second, the large‐scale and reproducible synthesis of FTP‐NPs with uniform physicochemical properties poses technical challenges that must be overcome to ensure consistency and quality for clinical use. Third, although our in vitro and in vivo studies indicate favorable biosafety, the long‐term safety, stability, and potential immunogenicity of peptidic nanomaterials require comprehensive evaluation through extended preclinical and clinical studies. Fourth, the regulatory and translational pathways for nanomedicine remain complex and evolving, which could prolong the timeline for clinical approval. Addressing these challenges will be critical to realizing the full translational potential of FTP‐NPs and ensuring their safe and effective integration into cancer therapy.

## Conflict of Interest

The authors declare no conflict of interest.

## Author Contributions

Y.C., Y.W., and H.S. contributed equally to this work. Y.C. was associated with conceptualization, methodology, investigation, visualization, and wrote the original draft. Y.W. and H.S. performed methodology and investigation. R.Z. contributed to conceptualization, methodology, software, and funding acquisition. J.Q. and X.L. performed formal analysis and data Curation. T.W. and Y.X. were associated with software. F.W. performed validation. A.W. performed formal analysis. B.W. performed the investigation. C.H. performed conceptualization and methodology. W.C. performed conceptualization, methodology, finding acquisition, and reviewed and edited the final manuscript. L.Z. performed visualization, supervision, project administration, and funding acquisition. All authors reviewed the manuscript.

## Supporting information



Supporting Information

Supporting Table 2

Supporting Table 3

## Data Availability

The data that support the findings of this study are available from the corresponding author upon reasonable request.
