# Supplementary material for: In Situ Assembly of Transformable Monopeptide on Activated Neutrophils Attenuates NETs‐Induced Hepatocellular Carcinoma Metastasis by Disrupting NE Nuclear Translocation
Source: Adv Sci (Weinh). 2025 Oct 24;13(1):e17415. doi: 10.1002/advs.202517415 (PMC12767106; doi:10.1002/advs.202517415)
Supplement: Supplementary file 1 — Supporting Information [file ADVS-13-e17415-s002.pdf]

# Supplementary Information

## *In Situ* Assembly of Transformable Monopeptide on Activated Neutrophils Attenuates NET-Induced Hepatocellular Carcinoma Metastasis by Disrupting NE Nuclear Translocation

Yichi Chen[1, 2, #], Yijun Wang[1, #], Haitao Shang[2, 3, #], Jiayue Qiu[4], Ruotian Zhang[1], Yuxiang Xiong[1], Tong Wang[1], Fengyi Wang[1], Anbang Wu[1], Xin Lin[2], Bolin Wu[2], Chen Huang[4], Wen Cheng[2, *], Lu Zhang[1, *]

[1]Guangdong Provincial Key Laboratory of Advanced Biomaterials, Department of Biomedical Engineering, Southern University of Science and Technology, Shenzhen 518055, China.

[2]Department of Ultrasound, Harbin Medical University Cancer Hospital, Harbin, China.

[3]Heilongjiang Province Key Laboratory of Research on Molecular Targeted Anti-Tumor Drugs, Harbin, China.

[4]State Key Laboratory of Quality Research in Chinese Medicines & Faculty of Chinese Medicine, Macau University of Science and Technology, Taipa, Macau SAR, 999078, China

[#]These authors contributed equally to this work.

[*]Correspondence should be addressed to: chengwen@hrbmu.edu.cn; zhanglu@sustech.edu.cn

## Materials and methods

### Data collection

We obtained hepatocellular carcinoma (TCGA-LIHC) transcriptomic data from the Cancer Genome Atlas (TCGA) database (https://portal.gdc.cancer.gov/), quantified as transcripts per million (TPM). Tumor samples with complete clinical annotations (overall survival (OS), age, stage, etc.) were retained for further analysis. Another HCC gene expression profile under the accession number E-TABM-36, containing 44 samples with available survival prognosis information, was obtained from the EBI Array database (https://www.ebi.ac.uk/arrayexpress/) as a validation set. Additionally, we curated 69 neutrophil and NETosis-related genes from previous studies to identify potential NET-associated biomarkers [33]

### The Preparation of NFTP and FTP

The fibrillar transformable peptide (FTP, PpIX-GFFVLK-EAIPMSIPPEVK) and non-fibrillar transformable peptide (NFTP, PpIX-GGGAAK-EAIPMSIPPEVK) were synthesized according to a previous study.[34] Using Fmoc-coupling chemistry, solid-phase peptide synthesis techniques were used to prepare the peptides. At a concentration of 20 mM in the first solution, the peptide was dissolved in DMSO. NFTP was obtained by the same method. A Q-Exactive liquid chromatography-quadrupole orbital trap mass spectrometer (high-resolution) was used to confirm the peptides.

### The formation of FTP-NPs and NE-induced transformation

A solution was made by dissolving the transformable peptide monomers, FTP and NFTP, in DMSO (20 mM). The peptide solution (5 µL) was combined with deionized water (0, 200, 400, 600, 800, 900, 980, and 995 µL) after being further diluted with DMSO (995, 795, 595, 395, 195, 95, 15, and 0 µL). To confirm the generation of NPs, measurements were made of the ultraviolet-visible (UV-vis) absorbance (UV-2600i, Japan) at approximately 410 nm and the fluorescence spectra (Cary 5000, Malaysia) at approximately 635 nm of solutions with different water contents.

NFTP-NPs and FTP-NPs were diluted with DMSO from the initial solution (20 mM) to 2 mM, respectively. Using the fast injection approach, 10 µL of DMSO solution was injected into 990 µL of deionized water to create 20 µM of new nanoparticles ($H_2O$: DMSO = 99:1, v/v) as an initial condition for the subsequent experiment. FTP-NPs (20 µM, 1000µl) were mixed with 2.4 µl of NE protein (8.3 µM, MA-0156, AkrivisBio) to promote the morphological transition of FTP-NPs into nanofibers, which were then cultivated at room

temperature. Following the incubation, 10 μL of the solution was added to a copper mesh at 0 and 24 h. Filter paper was used to remove any extra droplets after an overnight stay. The samples were then stained for five minutes with uranyl acetate and then rinsed for one minute with water (10 μL). Finally, dried samples were observed by TEM (Talos 120C, Thermo Fisher Scientific, USA). DLS (Zetasizer Nano ZS, UK) was used to assess the solution for hydrodynamic size and zeta potential at 0 and 24 h following the incubation. Then, the nanofiber structure at 0 h and 24 h after incubation of FTP-NPs was examined with Circular Dichroism (CD) Spectra (Chirascan, UM, Britain). In addition, the variation of fluorescence emission spectra of PpIX by Fluorescence spectrophotometer (Cary5000, Malaysia) at about 635 nm was also used to demonstrate the transformation of FTP-NPs.

Pyrene molecules were employed as an indicator to determine the critical aggregation concentration (CAC) of nanoparticles by comparing the fluorescence of their third and first emissive peaks. First, NPs were diluted to different concentrations (0.01, 0.05, 0.1, 0.5, 1, 5, 10, 20, 30, and 50 μM), then 999 μl of NPs of each dilution was incubated with 1 μl of pyrene acetone solution (0.1 mM) at 37 °C for 2 h. The fluorescence spectra of pyrene (excitation, 335 nm) in different NPs dilutions were recorded. The fluorescence intensity ratio (I3/I1) of the third and first emissive peaks was measured for CAC calculation. The stability of FTP-NPs and NFTP-NPs was studied in dd$H_2O$ and medium solution. The mixture was incubated at room temperature, followed by size measurements at predetermined time intervals up to 168 h.

**Simulation of all-atom molecular dynamics**

The molecular structures of peptide NFTP and FTP monomers were constructed using AlphaFold II and Gaussian 16W software packages. Density functional B3LYP was used in the Gaussian structure optimization process, and DFT-D3 (BJ) dispersion correction was applied. The basis set of all atoms was 6-31G (d). Dynamic simulations were run using the Gromacs 2022.4 package based on the AMBER14SB force field. The system configuration was visualized using VMD and pymol software, and images were mainly generated using QtGrace and PyMOL v1.8.2.2 software. The simulations were performed in a water box containing 20 NFTP and FTP molecules. NFTP and FTP systems containing NaCl were simulated to neutralize the charges on the amino acid residues. The steepest descent method was used for energy minimization. Bond lengths were constrained by the LINCS algorithm. Non-binding LJ interactions were cut off at 1.2 nm. The electrostatic field was treated using the particle mesh Ewald scheme. All production runs were performed in NPT ensemble using the V-scale

coupling scheme, temperature maintained at 300K, the Parinello-Rachman coupling scheme, pressure maintained at 1.0Pa, and isotropic coupling type simulations. The time steps for the pressure and temperature coupler were 1 and 1 fs. MD simulations were performed with periodic boundary conditions with a time step of 1 fs; simulations lasted 40 ns, and structural coordinate information was recorded every 10 ps.

**Molecular docking**

Molecular docking studies were performed using MOE (Molecular Operating Environment) software. First, the protein was preprocessed using the Structure Preparation tool in MOE, which included removing ligands and water molecules, adding hydrogen atoms, repairing missing residues, and performing energy minimization to optimize the structure. Subsequently, the Site Finder module in MOE was used to predict and confirm the active site of the protein, providing the binding target coordinates for subsequent molecular docking. The ligand molecule was drawn using AlphaFold II and Gaussian 16W software packages and imported into MOE, and the structure was optimized using the Energy Minimize module to obtain the highest affinity conformation. During the docking process, the Dock module of MOE was used to simulate the binding of the ligand molecule to the protein active site. The docking parameters were set to Flexible Docking mode, which allows the ligand to be flexibly adjusted within the binding site while the protein remains rigid. The docking process used the London dG scoring function for preliminary screening, and then the GBVI/WSA dG scoring function was used to score the binding conformation accurately.

**Cell line culture, isolation of neutrophils, and animals**

The Hepa1-6/luc cell line obtained from Harbin Medical University Cancer Hospital and murine vascular endothelial cell (C166 cells) purchased from SHANGHAI WHELAB BIOSCIENCE LIMITED were maintained in DMEM supplemented with 10% FBS at 37°C in a humidified atmosphere containing 5% $CO_2$. C57BL/6J mice were acquired from TOPBIO (Shenzhen) and raised to a gestational age of 4-6 weeks. All animals used in this study were by the guidelines of the Institutional Animal Care and Use Committee (IACUC) authorized by Southern University of Science and Technology; all the procedures on animals were implemented (Resolution number is SUSTech-JY202310107). Neutrophil suspension was extracted from mouse bone marrow according to the manufacturer's instructions (Solarbio), and the cell suspension was carefully layered on top of the reagent to form a gradient interface. The tubes were centrifuged at 1000g at room temperature for 30 min. After centrifugation, the neutrophil layer was collected and washed with PBS, then centrifuged at 250g for 10

min. The supernatant was discarded, and the red blood cells were lysed with RBC lysate. The neutrophils were then washed with PBS again, and the supernatant was discarded. The cells were resuspended in culture medium and used for further experiments.

**Hemolysis Assay of FTP-NPs**

To assess the hemolytic activity of FTP-NPs, red blood cells (RBCs) were harvested from C57BL/6 mice (aged 6–8 weeks, female). The mice were euthanized by cervical dislocation, and blood was collected via cardiac puncture into heparinized tubes. Blood was then immediately diluted with an equal volume of phosphate-buffered saline (PBS) and centrifuged at 1500 g for 10 minutes to isolate the RBCs. The supernatant was discarded, and the RBC pellet was washed three times with PBS to remove any residual plasma and other cellular components. The final RBC suspension was adjusted to a 2% hematocrit using PBS. FTP-NPs were synthesized as described previously. Briefly, FTP-NPs were prepared by self-assembly of peptidic nanomaterials and adjusted to various concentrations (0, 50, 100, 200, 400, 800, 1000 μg/mL). Each concentration was tested in triplicate. For the hemolysis assay, 200 μL of the 2% RBC suspension was incubated with 200 μL of FTP-NPs at the specified concentrations in a 96-well plate for 1 hour at 37°C under gentle shaking. The negative control (NC) consisted of RBCs suspended in PBS, while the positive control (PC) was treated with H2O, which completely lyses RBCs. After incubation, the samples were centrifuged at 1500 g for 10 minutes to remove unbound particles, and the supernatant was collected. The hemoglobin released into the supernatant was quantified by measuring the absorbance at 541 nm (A541) using a microplate reader. The percentage of hemolysis was calculated using the following formula: Hemolysis %= $(A_{sample}-A_{NC})/(A_{PC}-A_{NC})$ *100%. Where $A_{sample}$ is the absorbance of the sample, $A_{NC}$ is the absorbance of the negative control, and $A_{PC}$ is the absorbance of the positive control.

***In vitro* cytotoxicity assay**

The CCK-8 assay was used to assess the cell viability of Hepa1-6/luc, resting neutrophils, and activated neutrophils. Following the seeding of $1*10^4$ cells per well on a 96-well plate, the cells were subjected to different dosages of FTP-NPs and NFTP-NPs (medium: DMSO = 99:1 v/v). After 24 hours, the cells were incubated for two hours with 10 μL of CCK-8 solution. Finally, a microplate reader (Tecan Infinite M200, Switzerland) was used to measure cell viability based on the absorbance values.

**Targeting the capability of NEBP**

To evaluate the targeting capability of NE-binding peptide (NEBP), the harvested resting neutrophils and

activated neutrophils were incubated with FTP-NPs for 1 h ($n$ = 3). For the cells from the NEBP-blocked group, excess NEBP was added and cultured for 30 min, and then replaced with FTP-NPs for another 1 h incubation. All of these cells were washed twice with PBS. Finally, we used flow cytometry to detect the fluorescence intensity of PpIX in cells.

**Scanning electron microscope (SEM) observation**

To enable the transformation into NFs, resting neutrophils were treated with NFTP-NPs and FTP-NPs (50 μM, medium: DMSO = 99:1, v/v) for 0, 0.5, 1.5, and 4 hours. For activated neutrophils, 100nM Phorbol-12-myristate-13-acetate (PMA; Sigma-Aldrich, St. Louis, MO, USA) was added to the culture medium with NFTP-NPs and FTP-NPs (50 μM, medium: DMSO = 99:1, v/v) for 0, 0.5, 1.5, and 4 hours. After a PBS wash, the cells were fixed overnight at 4°C in a 2.5% glutaraldehyde solution. Following a succession of ethanol dehydration steps (from 30, 50, 70, 80, 90, and 99.5%), the cells underwent critical point drying using hexamethyldisilazane (HMDS). To confirm that the fibrous coating was formed, we examined the cells by SEM (Hitachi-SU8230).

***In vitro* binding and retention of FTP-NPs with activated neutrophils**

In order to compare the differences of FTP-NPs in binding with resting neutrophils and activated neutrophils, resting neutrophils were treated with NFTP-NPs and FTP-NPs (50 μM, medium: DMSO = 99:1, v/v) for 4 and 12 hours, for activated neutrophils, 100 nM Phorbol-12-myristate-13-acetate (PMA; Sigma-Aldrich,St. Louis, MO, USA) was added to the culture medium with NFTP-NPs and FTP-NPs (50 μM, medium: DMSO = 99:1, v/v) for 4 and 12 hours, washed 3 times with PBS, and cell precipitation was suspended with 100 μL PBS. Flow cytometry was used for a quantitative study (BD FACS Canto SORP, USA).

**Confocal laser scanning microscopy (CLSM) for elastase translocation and cellular imaging**

We evaluated NFTP and FTP's capacity for targeting and translocation inhibition. In a confocal microscopy dish, neutrophils were planted, and they were then treated at 37 °C with NFTP-NPs (50 μM, medium: DMSO = 99:1, v/v), FTP-NPs (50 μM, medium: DMSO = 99:1, v/v), PMA+NFTP-NPs (50 μM, medium: DMSO = 99:1, v/v), and PMA+FTP-NPs (50 μM, medium: DMSO = 99:1, v/v). At 0 and four hours, respectively, CLSM (LSM980, Zeiss, Germany) was used to examine the samples to confirm that NFTP-NPs and FTP-NPs could target activated neutrophils and inhibit translocation. After being cultured with PMA+FTP-NPs for 4 hours, the cells were additionally incubated with neutrophil elastase (NE) antibody (ab314916, abmart) and stained with DAPI for CLSM imaging (LSM980, Zeiss, Germany).

**Neutrophil Elastase Activity Assay**

To evaluate the distribution of active NE on the surface and inside of neutrophils, we used a kit (AkrivisBio's Neutrophil Elastase Activity Assay, MA-0156), seeded neutrophils in confocal microscopy dishes, and then treated them with NFTP-NPs (50 μM, medium: DMSO = 99:1, v/v), FTP-NPs (50 μM, medium: DMSO = 99:1, v/v), PMA+NFTP-NPs (50 μM, medium: DMSO = 99:1, v/v), and PMA+FTP-NPs (50 μM, medium: DMSO = 99:1, v/v) at 37°C. Because the Neutrophil elastase in the kit cleaves a concrete fluorogenic substrate which releases AFC, providing intense fluorescence, 50 μl of MeOSucc-AAPV-AFC mix (Assay Buffer: MeOSucc-AAPV-AFC = 48:2) was added at 4 h, and after incubation for 1 h, the samples were examined by CLSM (LSM980, Zeiss, Germany).

**Quantitative analysis of NE activity**

To determine the concentration of FTP-NPs for effective activity inhibition of NE, neutrophils ($2.5\times10^5$cells) were cultured in RPMI 1640 medium containing 10% FBS in a 96-well plate. The cells were exposed to PMA, PMA +NFTP-NPs, and PMA +FTP-NPs in different concentrations of NPs (0, 25, 50, 100, and 200 μM, medium: DMSO = 99:1, v/v) for 4 h to obtain the solution.[35,36] Quantitative analysis was carried out by a NETosis Assay Kit (Cayman Chemical, USA).

**Quantitative induction analysis of NETs *in vitro***

Meanwhile, neutrophils ($2.5\times10^5$cells) were also bedded on coverslips (Biosharp, BS-14-RC) pretreated with poly-L-lysine (P4707, Sigma) for 30 min before addition of PBS, PMA (100 nM), PMA (100 nM)+NFTP-NPs (50 μM, medium: DMSO = 99:1, v/v), PMA (100 nM)+FTP-NPs (50 μM, medium: DMSO = 99:1, v/v), and PMA+NEi (50 μM, Sivelestat, MCE, Shanghai). After 4 h incubation at 37°C, all culture mediums were discarded, and Cit H3 (Cat.no.ab5103, Abcam, Cambridge, England, UK) and MPO (Cat.no.66177-1-Ig, Proteintech, Wuhan, Hubei, China) were observed by immunofluorescence staining under a CLSM (LSM980, Zeiss, Germany).[37]

**Enzyme-linked immunosorbent assay (ELISA)**

According to the manufacturer's instructions, MPO-DNA levels in cultured supernatant were analyzed by an ELISA kit (MEIMIAN, China).[38] Briefly, samples were added to each well and incubated with anti-MPO and anti-DNA antibodies for 2 h at room temperature. Samples were washed with incubation buffer and visualized with ABST substrate solution for 40 min at room temperature. The OD value was then measured by a microplate

reader (Tecan Infinite M200, Switzerland) at 405 nm. The supernatant NE (BY-EM220848, Nanjing BYabscience Technology Co.,Ltd) concentrations were also quantified by ELISA following the manufacturer's instructions. All the values were determined spectrophotometrically by the absorbance at 450 nm using a microplate reader (Tecan Infinite M200, Switzerland).

**DNA release measurement**

Neutrophils ($2.5\times10^5$cells) were bedded on coverslips (Biosharp, BS-14-RC) pretreated with poly-L-lysine (P4707, Sigma) for 30 min before addition of PBS, PMA, PMA +NFTP-NPs (50 μM, medium: DMSO = 99:1, v/v), PMA +FTP-NPs (50 μM, medium: DMSO = 99:1, v/v), and PMA+NEi (50 μM, Sivelestat, MCE, Shanghai). After 4 h incubation at 37°C, all culture mediums were discarded, and the cells were incubated with SYTOX Green dye (CTrueBlue, China). NET formation was observed by CLSM (LSM980, Zeiss, Germany).

**Actin polymerization measurement**

To assess F-actin kinetics, neutrophils ($2\text{x}10^6$/mL) were incubated for 0, 0.5, 1.5 and 3 h with PMA, PMA+NFTP-NPs (50 μM, medium: DMSO = 99:1, v/v) and PMA+FTP-NPs (50 μM, medium: DMSO = 99:1, v/v) NPs at 37°C, then the cells subsequently stained with Alexa Fluor 488 phalloidin (Beyotime, Shanghai, China) and were observed by CLSM (LSM980, Zeiss, Germany).

**Cell adhesion assay**

Neutrophils ($5\times10^5$cells/well) treated with PMA, PMA+NFTP-NPs, and PMA+FTP-NPs were seeded onto coverslips in 24-well plates for 4 h to generate NETs.[38] Hepa1-6/luc cells ($1\times10^5$cells/well) were labeled by DID (10μmol/L, Beyotime, Shanghai, China) for 20 min at 37˚C in an atmosphere of 5% $CO_2$ and added to each well. After incubation for 30 min, the coverslips were washed with PBS, fixed with 4% paraformaldehyde, stained with DAPI, and observed under CLSM, and also recorded by a flow cytometer (BD FACS Canto SORP, USA). All the experiments were performed thrice. For SEM observation, the same cells were treated and fixed for an entire night at 4°C in a 2.5% glutaraldehyde solution. Following a succession of ethanol dehydration steps (30, 50, 70, 80, 90, and 99.5%), the cells underwent critical point drying using hexamethyldisilazane (HMDS). To confirm that the fibrous coating was formed, we examined the cells using a scanning electron microscope (Hitachi-SU8230).

**NETs isolation and cell treatment**

Freshly isolated neutrophils were stimulated with PBS, PMA, PMA+NFTP-NPs, PMA+FTP-NPs, and PMA+NEi for 4 h to stimulate NETs formation. Cells were then centrifuged at 480g for 10 minutes. The supernatant (NETs rich media) was then centrifuged at 18000g for 15 mins to form a pellet. The obtained pellet contained the mixture of chromatins and proteins, which was then resuspended in cell culture media to treat cancer cells, which was collected and respectively named as resting neutrophils, Control, NFTP-NPs, FTP-NPs, and NEi, then stored at -80°C for further use.

**Cell proliferation assay**

Resting neutrophils (Neu)-treated, Control (Neu+PMA)-treated, NFTP-NPs (Neu+PMA+NFTP-NPs)-treated, FTP-NPs (Neu+PMA+FTP-NPs)-treated, and NEi (Neu+PMA+NEi)-treated Hepa1-6/luc cells and Hepa1-6/luc cells ($2\times10^3$cells/well) were seeded on 96-well plates. Cell proliferation was assessed using the Cell Counting Kit-8 (Biosharp, China) following the manufacturer's instructions. The final optical density (OD) value was measured daily for 3 consecutive days using a microplate reader at 450 nm (Tecan Infinite M200, Switzerland). Each condition was performed in triplicate.

**EdU staining**

EdU cell proliferation staining was performed using an EdU kit (BeyoClick™ EdU Cell Proliferation Kit with Alexa Fluor 594, Beyotime, China).[39] Briefly, Hepa1-6/luc cells ($1 \times 10^4$ cells/well) were cultured on round coverslips in 12-well plates for 12 h. Subsequently, cells were incubated with EdU for 2 h, fixed with 4% paraformaldehyde for 15 min, and permeated with 0.3% Triton X-100 for another 15 min. The cells were incubated with the Click Reaction Mixture for 30 min at room temperature in the dark. The fluorescence signal was then quickly inspected via a fluorescent microscope (Axiovert 200, Zeiss, Germany) and recorded by a flow cytometer (BD FACS Canto SORP, USA).

**Colony formation assay**

Hepa1-6/luc cells were collected and seeded into 6-well plates with 2000 cells per well, and cultured at 37 ◦C for 24 h. The cells were treated with Resting neutrophils, Control, NFTP-NPs, FTP-NPs, and NEi groups, and continued to be cultured for about 10 days until clonal clusters were formed. When the experiment was terminated, the cells were washed with 1xPBS 3 times, fixed with 4% paraformaldehyde for about 15 min, stained with 0.5% crystal violet staining solution for 15 min, followed by rinsing with deionized water and air-drying before capturing images (Axiovert 200, Zeiss, Germany).

### Cell migration and invasion assay

To produce cell gaps for cell scratch tests, Hepa1-6/luc cells were injected into culture inserts (IBIDI, Germany). After 24 hours, the cells were treated with Resting neutrophils, Control, NFTP-NPs, FTP-NPs, and NEi groups. Cell migration was monitored during the overnight culture of the cells. Hepa1-6/luc cells ($5\times10^5$cells/well) were placed in the upper chamber of an 8μm transwell system and incubated in serum-free DMEM, while various treated media with 20% FBS were added to the lower chamber of 24-well plates. After incubation for 48 h, the transmembrane cells were washed, fixed with 4% paraformaldehyde, stained with crystal violet, and observed under a microscope (Axiovert 200, Zeiss, Germany). All the experiments were performed thrice. The Transwell invasion assay was performed using a similar procedure. Hepa1-6/luc cells ($1\times10^6$cells/well) were seeded in the upper chamber of an 8μm transwell system coated with Matrigel (356231, Corning, New York, USA) and incubated in serum-free DMEM. The remaining operations were performed according to the steps of the migration assay. All the experiments were performed thrice.

### Matrigel tube formation assay

Murine C166 endothelial cells ($3\times10^4$cells/well) were placed on 96-well plates coated with Matrigel (356231, Corning, New York, USA) and incubated with various treatments. After incubation for six h, capillary-like structures were observed and counted under a microscope (Axiovert 200, Zeiss, Germany). All the experiments were performed thrice.

### *In vitro* permeability assays and trans-endothelial invasion assays

For *in vitro* permeability assays, C166 cells were treated with Resting neutrophils, Control, NFTP-NPs, FTP-NPs, and NEi groups for 48 h to form an endothelial monolayer, and then Evans Blue (PHYGENE, China) was added to the upper layer of the transwell filters (0.4 μm, Corning). After 30 min, the medium in the lower layer of the chamber was collected to measure the absorbance at 620 nm (Tecan Infinite M200, Switzerland).

For trans-endothelial invasion assays, $2\times10^6$ Hepa1-6/luc cells were seeded into transwell filters (8 μm, Corning) with endothelial cell monolayers pretreated with resting neutrophils, Control, NFTP-NPs, FTP-NPs, and NEi groups. After incubation for 48 h, the transmembrane cells were washed, fixed with 4% paraformaldehyde, stained with crystal violet, and observed under a microscope (Axiovert 200, Zeiss, Germany). All the experiments were performed thrice.

### RNA extraction and quantitative real-time polymerase chain reaction

Total RNA was extracted and purified using a miRNeasy Mini Kit (FOREGENE, China). Quantitative real-time polymerase chain reaction (qRT-PCR) was performed in triplicate in the ABI 7500 fast real-time PCR System (Applied Biosystems, USA). The mRNAs were normalized with 18s. The following primers were used for PCR detection: E-cadherin, N-cadherin, Vimentin, and α-catenin.

**Western blot**

Western blot analysis was performed to evaluate the levels of relative proteins in Hepa1-6/luc cells and Neutrophils.[38] To evaluate NETs release, the neutrophils were exposed to PMA, PMA+NFTP-NPs, and PMA+FTP-NPs for 12 h. Following treatment, the cells underwent three PBS washes for WB to measure the expression of CitH3 proteins (Cat. no. ab5103, Abcam, Cambridge, England, UK). Furthermore, to evaluate EMT of Hepa1-6/luc cells, the cells underwent three PBS washes for WB to measure the expression of E-cadherin (Abmart, China), N-cadherin (Abmart, China), Vimentin (Abmart, China), and β-catenin (Abmart, China).

***In vivo* subcutaneously implanted tumor models**

The C57BL/6J mice with subcutaneous Hepa1-6/luc xenografts (grew up to roughly 100 $mm^3$) were separated into four groups according to the different treatments to perform the *in vivo* tumor therapy. PBS, NFTP-NPs, FTP-NPs, and NEi (300 μM in PBS: DMSO = 95:5, 200 μL) were administered intravenously (i.v.) five times every two days to mice. aPD-1 (BE0146, Bio X Cell, USA) was injected intraperitoneally into each mouse at 200μg once every 4 days for 3 doses. Throughout the experiment, the tumor volumes and body weight were measured once every four days. The formula tumor volume=length*1/2 $width^2$ was used to get the tumor volume; at the same time, IVIS (AniView SE, Guangzhou Biolight Biotechnology Co., Ltd) was used to calculate bioluminescence signals *in vivo*. After therapy (day 20), major organs and blood samples were taken for histological and biochemical investigation as part of a safety assessment. Servicebio carried out the histological evaluation and blood biochemistry analyses. Major organ slices were utilized to look for possible histological alterations and immunofluorescence staining to evaluate the expression of E-cadherin, N-cadherin, Vimentin, β-catenin, Ki67, and NETs marker of MPO and CitH3.

**Immune Profiling and Cytokine Analysis in Tumor Microenvironment via Flow Cytometry and ELISA**

To study the immune cells in tumors, subcutaneous tumors from different groups were collected by surgery, and then homogenized into single-cell suspensions according to a well-established procedure. To analyze the

effector T cells ($CD45^+CD3^+CD8^+$)/($CD45^+CD3^+CD4^+$), tumor cells were stained with anti-CD45-FITC (Biolegend), anti-CD3ε-PerCP-Cy5.5 (Biolegend), anti-CD4-APC (Biolegend), and anti-CD8a-PE (Biolegend) antibodies according to the standard protocol. For evaluation of NK cells ($CD45^+CD3^-NK1.1^+$), tumor cells were stained with anti-CD45-FITC (Biolegend), anti-CD3-PE (Biolegend) and anti-CD49b-APC (Biolegend) antibodies and examined using flow cytometry. For MDSCs ($CD45^+CD11b^+Gr\text{-}1^+$) analysis, tumor cells were stained with anti-CD45-FITC, anti-CD11b-BrilliantViolet-510 (Biolegend), anti-F4/80-PE (Biolegend), and anti-Gr-1-APC (Biolegend) antibodies for flow cytometry examination (BD FACS Canto SORP, USA). The software FlowJo V10.6.2 was used for data analysis. To further explore the immunomodulatory effects of FTP-NPs. Tumor tissues were harvested for immune profiling. Plasma cytokine levels, including IL-6, IL-10, and TNF-α, were measured using commercially available ELISA kits to assess the immune activation induced by FTP-NPs.

**Experimental liver and lung metastasis model**

Six-week-old male C57BL/6J mice (20–25 g) were used to establish liver and lung metastasis models. Hepa1-6/luc cells (1 × 10^6 cells) were injected into the spleen or tail vein of the mice to induce liver or lung metastasis, respectively. For the liver metastasis model, Hepa1-6/luc cells were injected into the spleen, allowing tumor cells to circulate and establish metastases in the liver. For the lung metastasis model, Hepa1-6/luc cells were injected via the tail vein, leading to the formation of metastases in the lungs. Following cell implantation, the treatment groups were injected intravenously every two days with the following: PBS (control), NFTP-NPs, FTP-NPs, or NE inhibitors (NEi), at a concentration of 300 μM in PBS: DMSO = 95:5 (200 μL). There were five mice in each group, and the control group received the same volume of PBS. After 16 days, mice were euthanized, and metastases were assessed macroscopically. Bioluminescence imaging was performed by injecting D-Luciferin (150 mg/kg) intraperitoneally, and tumor burden was quantified using the IVIS Spectrum system (PerkinElmer). For histological analysis, liver and lung tissues were fixed in 4% paraformaldehyde (PFA), paraffin-embedded, and sectioned (5 μm). Hematoxylin and eosin (H&E) staining was used for morphological evaluation, and immunohistochemical staining for myeloperoxidase (MPO) and citrullinated histone H3 (CitH3) was performed to identify NETs. Immunofluorescence staining for NET markers was also conducted, and metastatic lesions were assessed under an inverted microscope (Axiovert 200, Zeiss, Germany).

**Cell preparation**

After being harvested, tissues were washed in ice-cold PBS (Hyclone, SH30256.01) and dissociated using SeekMate Tissue Dissociation Reagent Kit A Pro (SeekGene, K01801301) or SeekMate Tissue Dissociation Kit C (SeekGene, K01501) according to the manufacturer's instructions. DNase I (Sigma, 9003-98-9) treatment was performed optionally, depending on the viscosity of the homogenate. After erythrocyte removal (Solarbio, R1010), cell count and viability were assessed using a Fluorescence Cell Analyzer (Countstar® Rigel, S2) or a SeekMate Tinitan Fluorescence Cell Counter (SeekGene, M002C) with AO/PI reagent. Removal of debris and dead cells (Miltenyi, 130-109-398/130-090-101) was performed when necessary. Finally, cells were washed twice with RPMI1640 (Gibco, 11875119) and resuspended at 1×10^6 cells/mL in RPMI1640 containing 2% FBS (Gibco, 10100147C).

**Single-cell RNA-seq library preparation and sequencing**

Tumor-bearing mice from the PBS and FTP-NPs groups were euthanized on day 20, and tissue samples were collected for single-cell RNA sequencing (scRNA-seq) analysis. Single-cell RNA-Seq libraries were prepared using SeekOne® Digital Droplet Single Cell 3' library preparation kit (SeekGene, Catalog No. K00202). Briefly, an appropriate number of cells were mixed with reverse transcription reagent and then added to the sample well in the SeekOne® chip S3. Subsequently, Barcoded Hydrogel Beads (BHBs) and partitioning oil were dispensed into corresponding wells separately in chip S3. After emulsion droplet generation, reverse transcription was performed at 42℃ for 90 minutes and inactivated at 85℃ for 5 minutes. Next, cDNA was purified from the emulsion droplets and amplified in a PCR reaction. The amplified cDNA product was then cleaned, fragmented, end-repaired, A-tailed, and ligated to a sequencing adaptor. Finally, the indexed PCR was performed to amplify the DNA representing 3' polyA region of expressed genes, which also contained the Cell Barcode and Unique Molecular Index. The indexed sequencing libraries were cleaned up with VAHTS DNA Clean Beads (Vazyme, N411-01), analyzed by Qubit (Thermo Fisher Scientific, Q33226), and Bio-Fragment Analyzer (Bioptic, Qsep400). The libraries were then sequenced on illumina NovaSeq 6000 with PE150 read length or DNBSEQ-T7 platform with PE150 read length.

**Single-cell RNA-seq data processing**

Single-cell RNA sequencing data were processed using the standard Seurat pipeline with UMI counts [40]. First, quality control was performed to remove low-quality cells with mitochondrial gene percentages greater than 25%, as well as genes not expressed in any cells. Highly variable genes were then selected using the

FindVariableFeatures function with the mean.var.plot (mvp) method to enhance the efficiency and accuracy of downstream analyses. Principal component analysis (PCA) [41] was conducted, and the optimal number of principal components was determined using both JackStraw and Elbow methods. Unsupervised clustering of cells was performed based on the selected principal components using the Louvain algorithm implemented in FindClusters. Clustering results were visualized using t-distributed stochastic neighbor embedding (t-SNE) [42].

**Cell type annotation**

For cell type annotation, we employed two complementary methods and intersected the results for final labels. First, we used the R package SingleR for automatic annotation, which assigns cell types based on the similarity between query and reference cells. [43,44] Second, we annotated clusters by evaluating marker gene expression using marker sets curated from the Cell Taxonomy database [45]. Additionally, the inferCNV algorithm [46] was applied to epithelial cells to infer copy number variations, thereby distinguishing malignant cells from endothelial cells using endothelial profiles as a reference.

**Single-cell profiling and prognostic deconvolution of neutrophil dynamics**

To elucidate the dynamic processes and functional characteristics of neutrophils during the PBS-to-FTP transition, we performed several complementary analyses at the single-cell level. First, pseudo-time series analysis was conducted using the R package Monocle2 [47] on log-normalized neutrophil gene expression data to model the differentiation trajectory. Next, we evaluated metabolic differences across neutrophil clusters at various pseudo-time points using the R package scMetabolism[48], which calculated KEGG pathway activity scores for each cluster. To investigate intercellular communication within the TIME, we applied CellChat[49] to decipher signaling interactions between neutrophil clusters and other cell types. Finally, differential expression analysis was performed using the FindAllMarkers method (thresholds: $|\log2FC| > 1$, *p-value* $< 0.050$) to identify differentially expressed genes among neutrophil clusters.

To link the neutrophil states with HCC prognosis, we used CIBERSORTx[50] to deconvolute TCGA-LIHC gene expression profiles based on a feature matrix derived from differentially expressed neutrophil genes. The optimal cutoff values for neutrophil content in the three states were determined using the surv_cutpoint function in survminer, and Kaplan-Meier survival analyses were conducted with the survival package to compare prognostic differences among patient groups.

**The construction and validation of the prognosis score model**

In this study, we developed two prognostic scores, namely, the NETs score and the FTP-NPs score, using integrative machine learning approaches based on Cox regression analysis and generalized boosted regression modeling (GBM). Both scores were established and optimized using the TCGA-LIHC dataset as the training cohort, with subsequent validation performed independently using the E-TABM-36 cohort.

To construct the NETs score, we initially selected candidate genes from a set of 69 NETs-related biomarkers through univariate Cox regression analysis, retaining genes significantly associated with overall survival ($p < 0.05$). A stepwise Cox regression model (StepCox, direction set as "both") s then applied using the Akaike Information Criterion (AIC) to iteratively identify an optimal subset of prognostic genes. Subsequently, a generalized boosted regression model (GBM) was trained on this subset of genes. GBM parameters, including the optimal number of trees (n.trees), were optimized via 10-fold cross-validation within the training dataset. Additional GBM hyperparameters were fixed as follows: interaction depth of 3, shrinkage rate of 0.001, and a minimum of 10 observations per node.

For the FTP-NPs score, we leveraged results from single-cell RNA sequencing analysis of tumor cells, identifying differentially expressed genes (DEGs) by comparing the FTP-NPs group with the PBS group. These DEGs were mapped onto bulk RNA sequencing data from the TCGA-LIHC and E-TABM-36 cohorts. Following a similar modeling pipeline, we employed stepwise Cox regression (StepCox, forward direction) to identify a prognostically relevant subset of DEGs. Subsequently, GBM modeling was conducted on this subset, using the same cross-validation-based parameter optimization procedure described above for the NETs score. For both scores, the final risk score (RS) for each patient was calculated using the optimized GBM model according to the following formula:

$$RS_i = \sum_{m=1}^{M^*} \gamma^{(m)} h^{(m)}(x_i)$$

where $x_i$ represents the expression features (selected gene expression levels) of sample i, $h^{(m)}(x_i)$ is the prediction of the m-th regression tree for sample i, $\gamma^{(m)}$ denotes the learning rate (shrinkage), and $M^*$ indicates the optimal number of trees selected via cross-validation.

To evaluate the prognostic performance of the established models (NETs score and FTP-NPs score), we conducted time-dependent receiver operating characteristic (ROC) curve analysis using the timeROC R package. Kaplan-Meier survival curves combined with log-rank tests were employed to assess differences in overall survival (OS) between high- and low-risk groups identified by each score. Subsequently, a comprehensive

nomogram was constructed by integrating both prognostic scores and clinical parameters (including age and tumor stage) using the rms and survival R packages to facilitate personalized survival predictions.

**Weighted Gene Co-expression Network Analysis (WGCNA)**

Weighted correlation network analysis (WGCNA) is an algorithm for identifying gene modules based on similar expression patterns and exploring their associations with biological traits. [51] In this study, we used the R package WGCNA to construct gene co-expression networks for HCC. First, A scale-free network was generated using an optimal soft-threshold power β of 5. Next, we calculated a dissimilarity Topological Overlap Matrix (TOM), which was then used for hierarchical clustering to identify co-expression modules (minClusterSize = 30, maxClusterSize = 6000). The Pearson correlation coefficients (PCC) between module eigengenes and FTP-NPs groups were computed and visualized using heatmaps. Modules significantly associated with FTP risk grouping were identified for further analysis.

**Immune infiltration**

We compared the differences in immune-related signature features between the two patient groups. The neutrophil recruiting score and immune-related pathways were estimated by performing ssGSEA using the corresponding gene list, which is listed in **Supplementary Table 3.** To further explore the difference in immune infiltration, the abundance of tumor-infiltrating immune cells was calculated by five algorithms, including MCP-counter, CIBERSORT, EPIC, Quantiseq, and Timer in the "IOBR" R package [52].

**Enrichment analysis**

GSEA was also performed using the log2(fold change) rank of all genes. ClusterProfiler is a universal enrichment analysis tool that supports GO, KEGG, and gene set enrichment analysis (GSEA) and can easily visualize the enrichment analysis results. The R package "clusterProfiler" [53] was used to perform Gene Ontology (GO) and Kyoto Encyclopedia of Genes and Genomes (KEGG) analyses. The hallmark gene set ("h.all.v7.4.symbols.gmt"), KEGG gene set ("c2.cp.kegg.v7.4.entrez.gmt"), and Reactome gene set ("c2.cp.reactome.v2023.2.Hs.entrez.gmt") were downloaded from the MSigDB database, [54] and enrichment scores for each pathway were calculated for all samples.

**Statistical analysis**

All statistical analyses were performed using R software (version 4.1.1). Data distribution normality was assessed with the Shapiro–Wilk test, and variance homogeneity was evaluated with Levene's test. Outliers were examined and excluded if necessary. Data are presented as mean ± standard deviation (SD) or percentages, and the sample size (n) for each experiment is provided in the corresponding figure legends. For comparisons between two groups, normally distributed data were analyzed using independent two-tailed Student's t-tests,

while non-normally distributed data were evaluated using the Wilcoxon rank-sum test. For comparisons across multiple groups, one-way ANOVA followed by Tukey's post-hoc test, or the Kruskal－Wallis test with Dunn's post-hoc test, was applied as appropriate. Pearson correlation analysis was used to calculate correlation coefficients. All statistical tests were two-sided, with statistical significance set at $p < 0.05$. Significance levels are indicated as *$p < 0.05$, **$p < 0.01$, ***$p < 0.001$, and ****$p < 0.0001$.

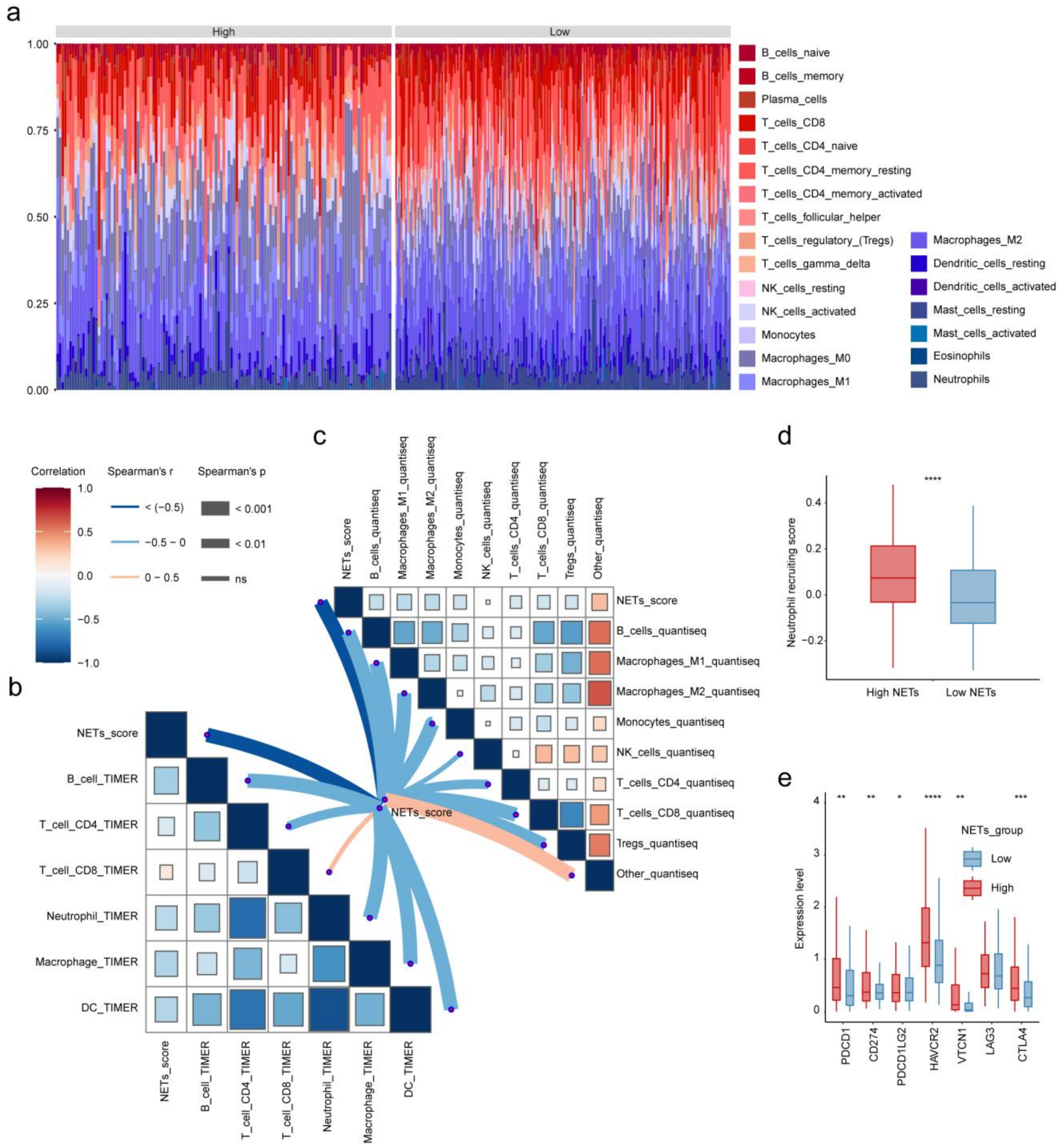


**Figure S1. Immune profiles associated with NETs score in HCC.** (**a**) The distribution of immune cell subset infiltration was calculated using the CIBERSORT algorithm. (**b, c**) Pearson correlations between NETs score

and fractions of immune cells using TIMER (b) and quanTIseq (c) methods. (**d, e**) Comparison of the neutrophil recruiting score (d) and the expression level (e) of the immune checkpoint genes between the low NETs and high NETs groups, the P-values were calculated with the Wilcoxon rank-sum test,*$p < 0.050$; **$p < 0.010$; ***$p < 0.001$; ****$p < 0.0001$; ns, no significance.

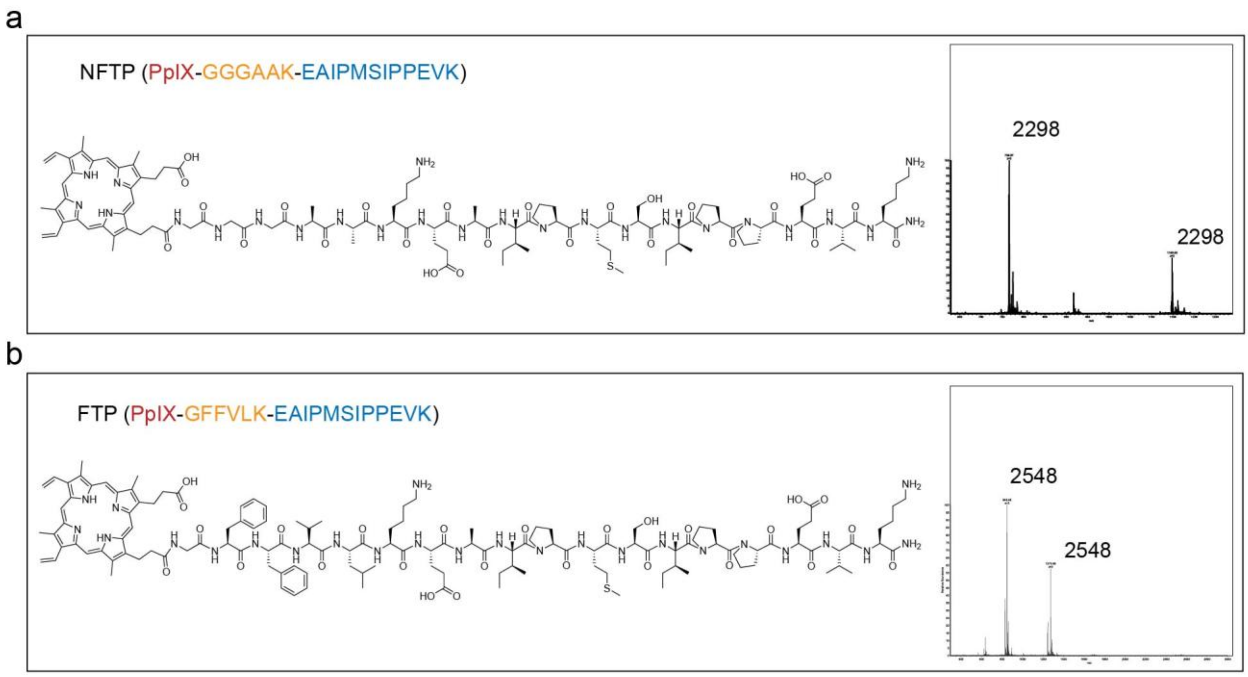


**Figure S2. Chemical structure and mass spectra of NFTP and FTP.** (**a**) PpIX-GGGAAK-EAIPMSIPPEVK. (**b**) PpIX-GFFVLK-EAIPMSIPPEVK. Experiments were repeated three times.

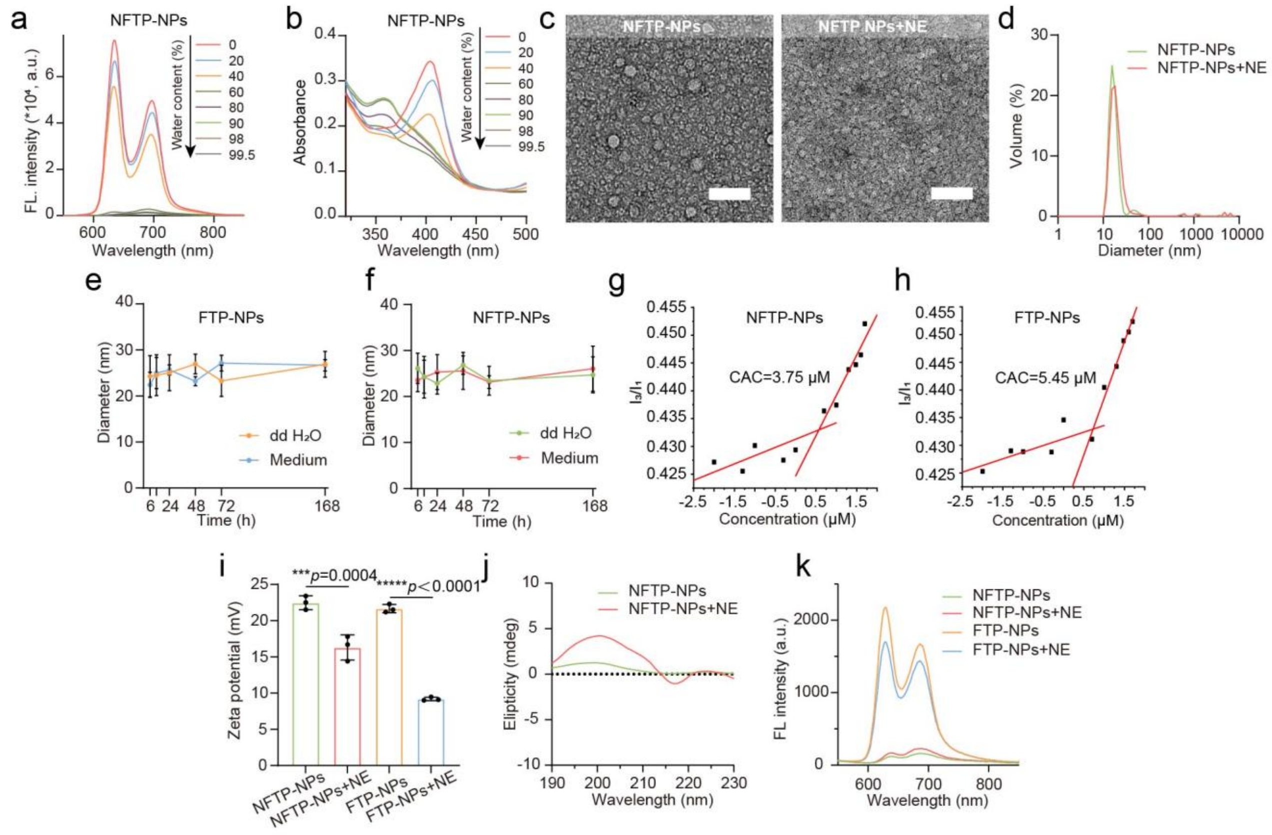


**Figure S3. The self-assembly of NFTP-NPs and FTP-NPs was stable, and the particulate formulation was kept even when incubated with NE.** (**a, b**) Changes in fluorescence and UV–vis absorption of FTP-NPs following the gradual addition of water (from 0 to 99.5%) to a solution of NPs in Dimethyl Sulfoxide (DMSO). Experiments were repeated three times. FL., fluorescence. a.u., arbitrary units. (**c**) Transmission electron microscope (TEM) images of NFTP-NPs and interaction with NE protein (Mw≈30 KDa) at 24 h. Scale bars, 100 nm. Experiments were repeated three times. (**d**) Variation in the size distribution of initial NPs and interaction with NE protein at 24 h. The molar ratio of NE protein/ peptide ligand was≈1/1000. mdeg, millidegrees. (**e, f**) Dynamic light scattering (DLS) measured the Nanoparticle stability of NFTP-NPs and FTP-NPs in $H_2O$ and Medium solution at room temperature. The concentration of NFTP-NPs and FTP-NPs used in the experiment was 20 μM. Experiments were repeated thrice. (**g, h**) Critical aggregation concentration (CAC) was measured using pyrene as a probe for NFTP-NPs and FTP-NPs. (**i**) NFTP-NPs and FTP-NPs with NE at 24h Zeta potential. The concentration of NFTP-NPs and FTP-NPs used in the experiment was 20 μM. Data are presented as mean ± s.d., $n$ = 3 independent experiments. Statistical significance was calculated by one-way ANOVA, ***$p$ < 0.001, ****$p$ < 0.0001. (**j**) Circular dichroism (CD) spectra of initial NFTP-NPs interaction with NE protein for 24 h. The concentration of NFTP-NPs used in the experiment was 20 μM. Experiments were repeated three times. mdeg, millidegrees. (**k**) Variation in fluorescence signal of initial NPs and NPs interaction with NE protein at 24h. The molar ratio of NE protein/ peptide ligand was≈1/1000. a. u., arbitrary units; FL., fluorescence.

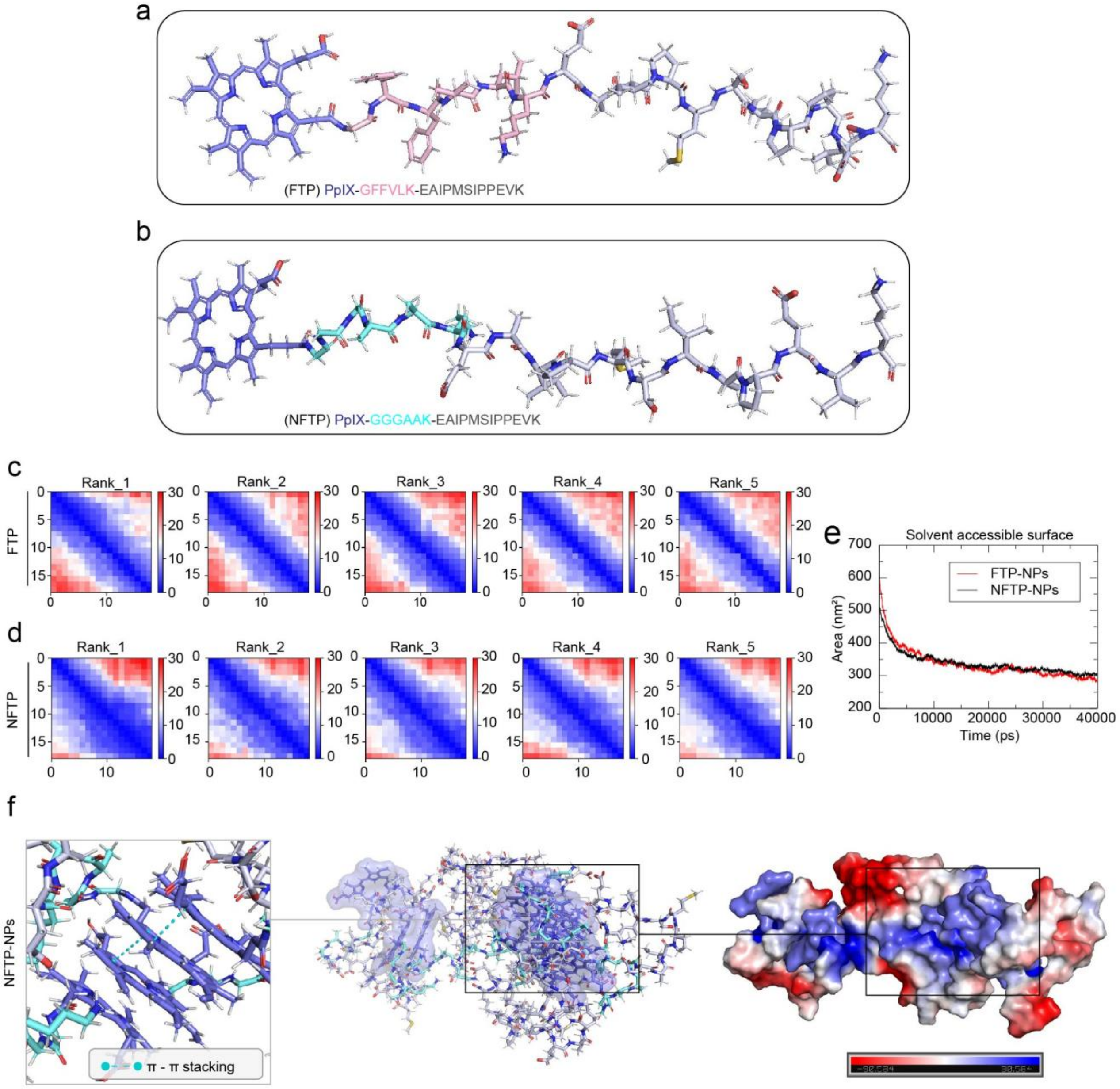


**Figure S4. Assembly of FTP-NPs and NFTP-NPs *in vitro*. (a, b)** The three-dimensional structure predicted by AlphaFold II. (**c, d**) The contact maps of the top five predicted structures obtained through AlphaFold II clustering. The x-axis and y-axis correspond to the residue indices of the peptide. The color gradient from blue to red represents the varying contact strength between residues, with blue indicating stronger contact (shorter distance) and red indicating weaker contact (longer distance). These contact maps display the conserved and variable contact patterns across the top five predicted structures, with rank_3 and rank_5 representing the highest-confidence predictions for FTP and NFTP, respectively. (**e**) The change in the solvent-accessible surface area of the convertible peptide monomer over time. (**f**) NFTP monomer self-assembly, with a schematic of the central PpIX hydrophobic domain (the blue surface structure in the center represents the hydrophobic region).

On the left is the schematic of the original porphyrin π-π stacking, and on the right is the electrostatic potential map of the self-assembled structure, where the hydrophobic region of the original porphyrin carries a positive charge.

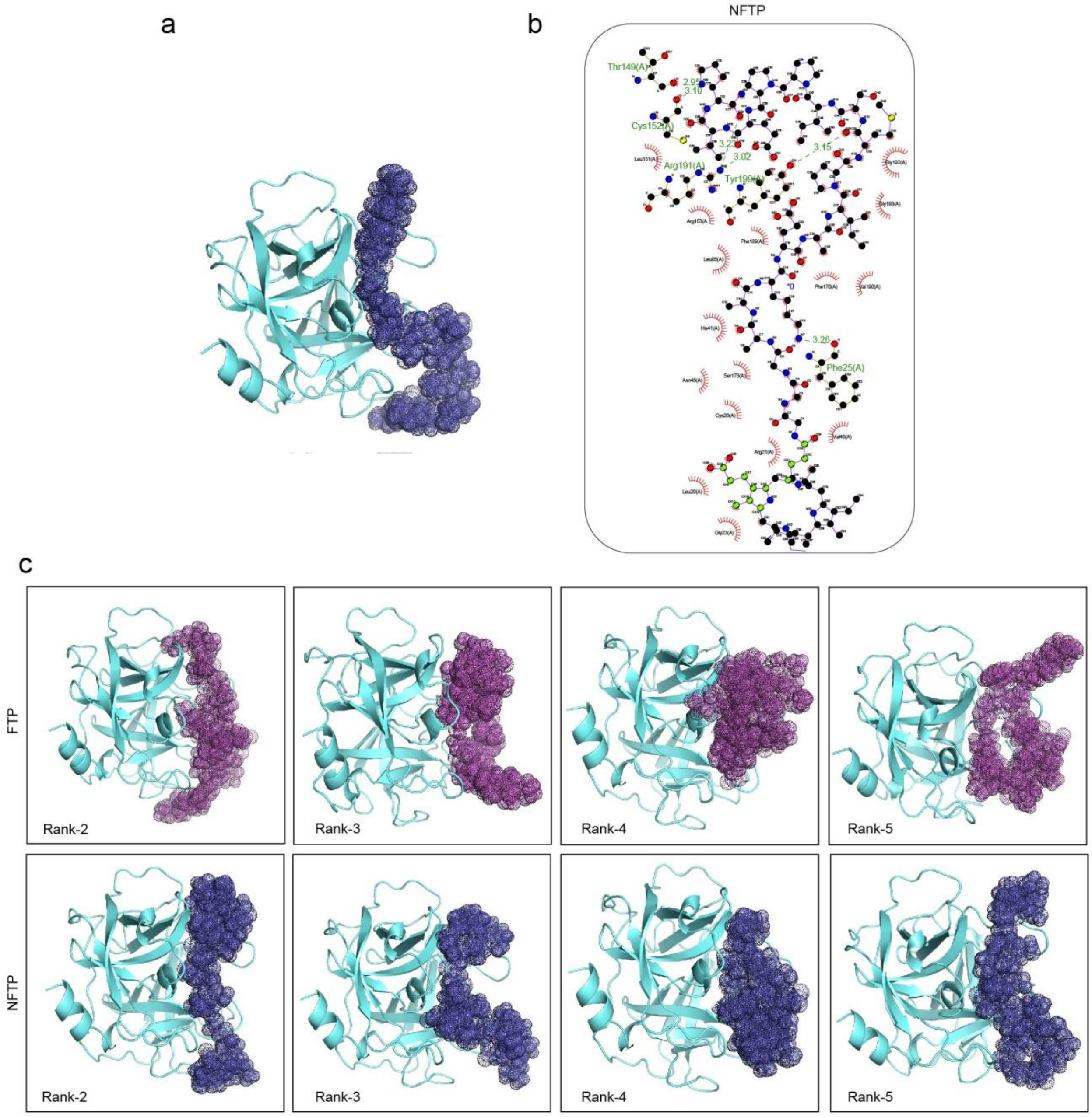


**Figure S5. Fibrillar transformation of FTP and NFTP *in vitro*.** (**a**) The highest-scoring conformation is based on the docking score function for the NFTP molecule, which has the strongest affinity. This conformation shows the NE protein in cyan cartoon representation, and the NFTP ligand is displayed as dots. (**b**) The 2D interaction schematic. (**c**) The dots represent the remaining four docking conformations ranked in the top five based on the docking score function for FTP and NFTP molecules.

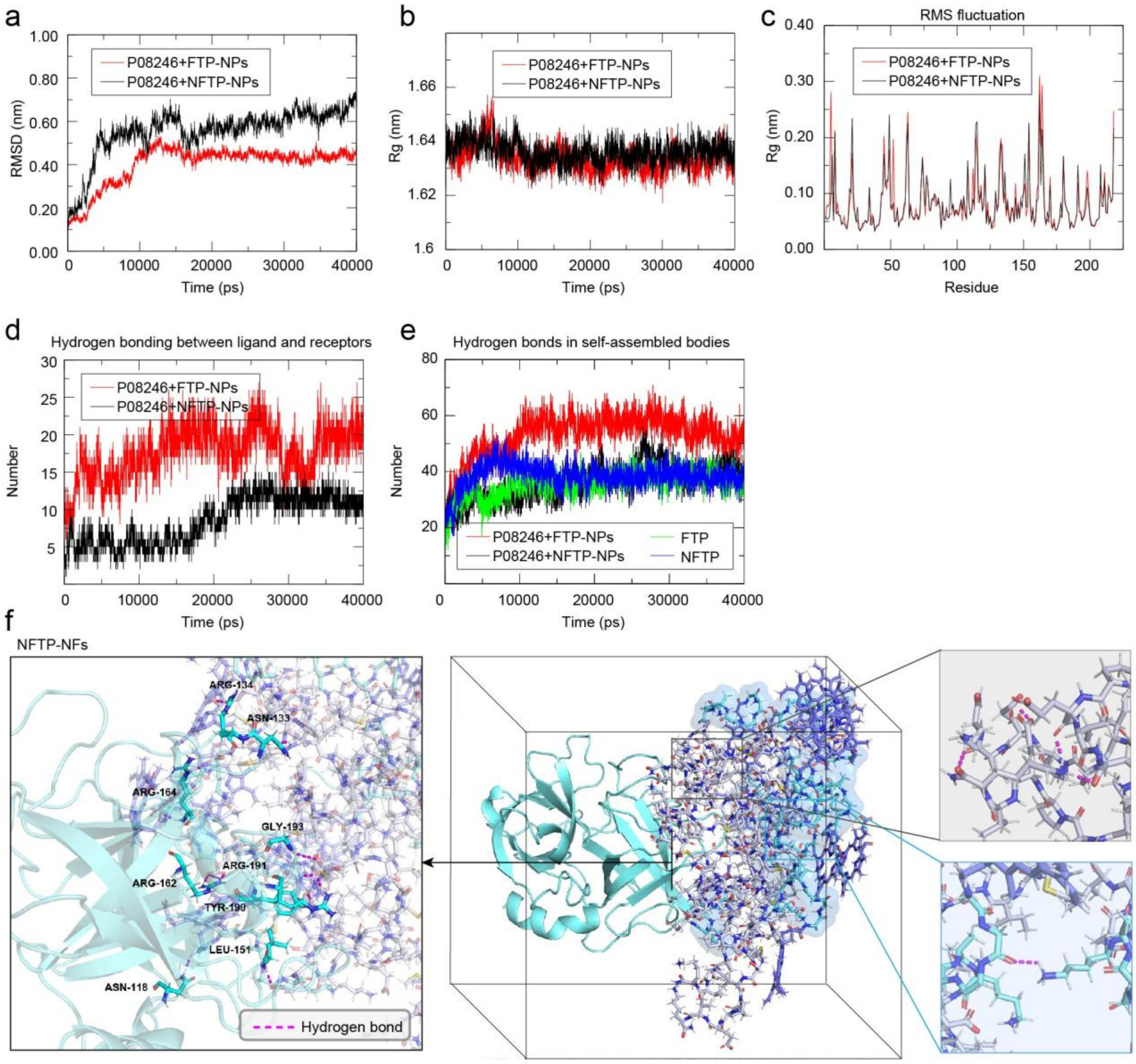


**Figure S6. Fibrillar transformation of FTP-NPs and NFTP-NPs *in vitro*. (a)** RMSD analysis of the backbone of NE (P08246) in the presence of FTP-NPs and NFTP-NPs. (**b**) Rg analysis of the backbone of NE (P08246) in the presence of FTP-NPs and NFTP-NPs. (**c**) RMSF analysis of the amino acid residues of NE (P08246) in the simulation, showing the fluctuations between FTP-NPs and NFTP-NPs. (**d**) Over time, the number of hydrogen bonds between NE (P08246) and FTP/NFTP varies. (**e**) The variation in the number of hydrogen bonds within the two convertible peptide assemblies in the presence of NE (P08246), as well as the variation in the number of hydrogen bonds within the two convertible peptide assemblies in the absence of NE (P08246). (**f**) Left: Residues involved in the interaction between NE (P08246) and NFTP (targeted interaction). Right: Interactions between the targeted peptide and fibril-converted peptide induced by the protein.

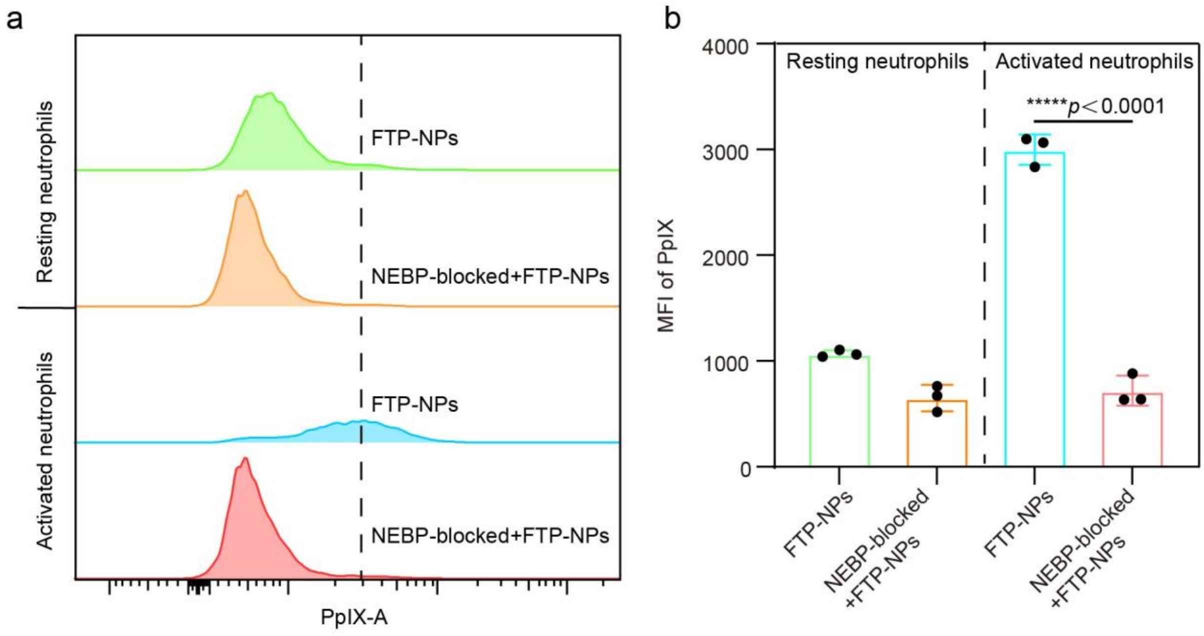


**Figure S7. Targeted ability of FTP-NPs.** (**a**) Representative Flow cytometry (FCM) profiles of PpIX fluorescence in cells with various treatments. The concentration of NFTP-NPs and FTP-NPs was 50 μM. (**b**) The corresponding intracellular mean fluorescence intensity with different treatments. The concentration of NFTP-NPs and FTP-NPs was 50 μM. Data are presented as mean ± s.d., $n = 3$ independent experiments: One-way ANOVA, ****$p < 0.0001$ calculated statistical significance.

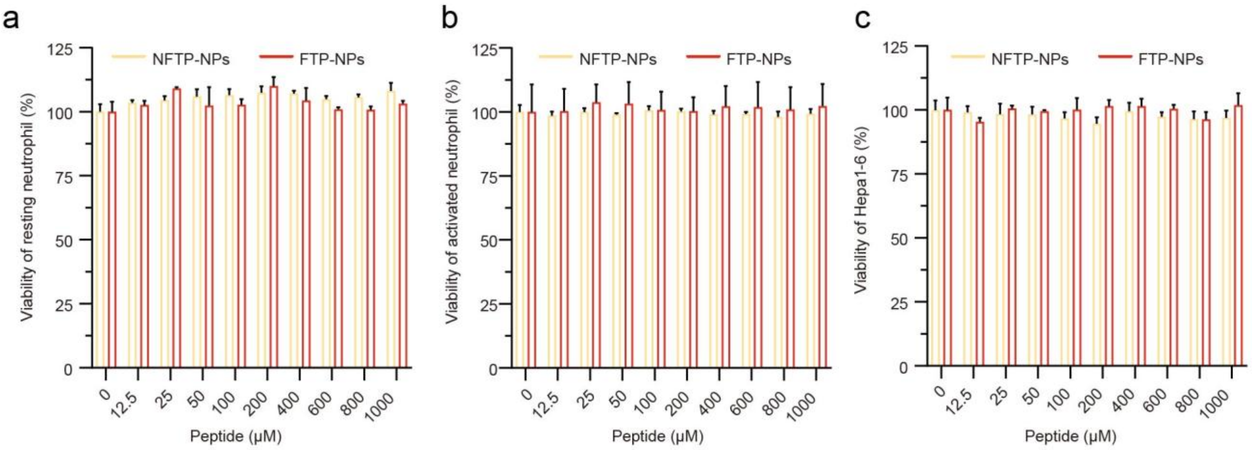


**Figure S8. Cytotoxicity of FTP-NPs and NFTP-NPs toward resting neutrophils (a), activated neutrophils (b), and Hepa1-6 cells (c).** No significant toxicity was observed in any of the cell types tested.

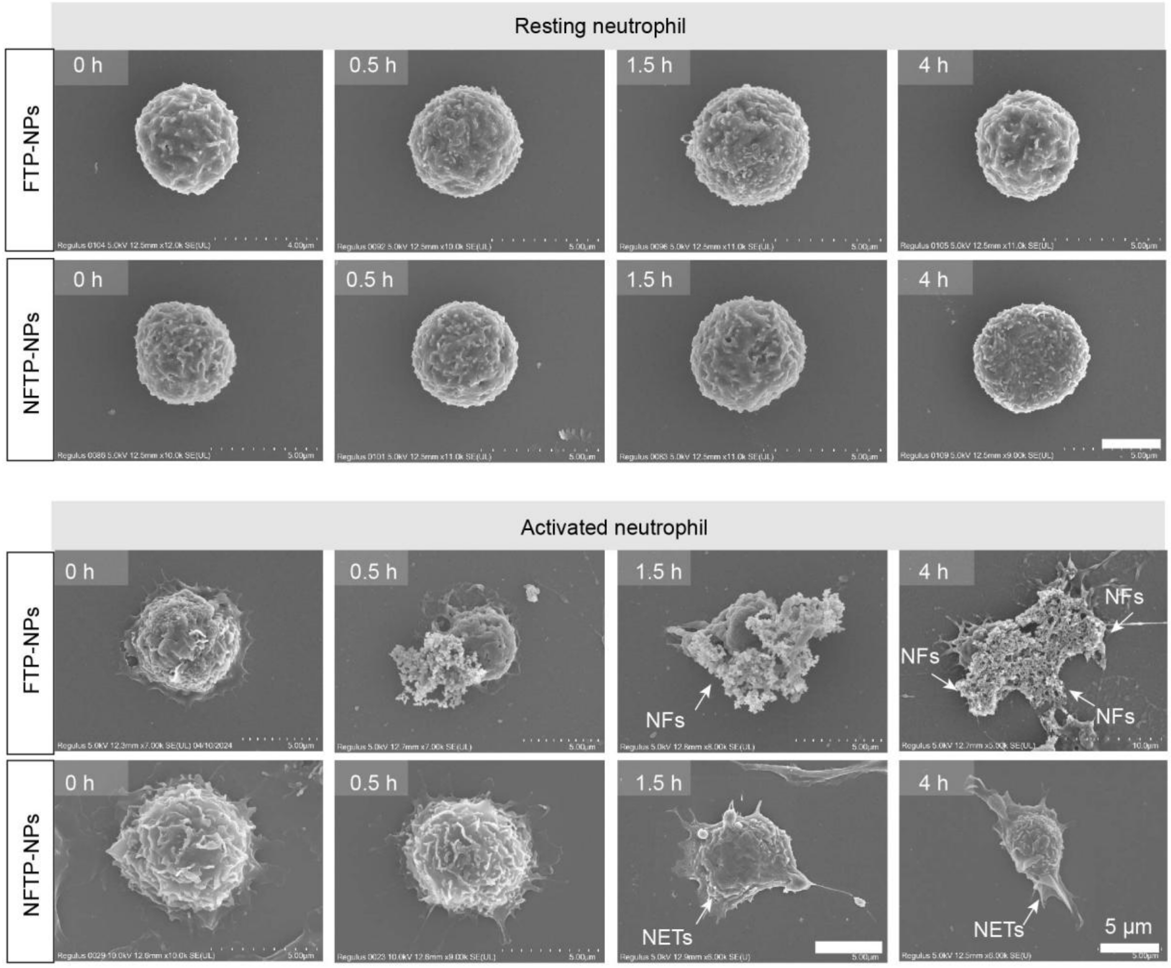


**Figure S9. Scanning electron microscope (SEM) images of resting neutrophils and activated neutrophils treated with NFTP-NPs and FTP-NPs for 0.5 h and 1.5 h.** Scale bars, 5 μm. The concentration of NFTP-NPs and FTP-NPs was 50 μM. Experiments were repeated three times.

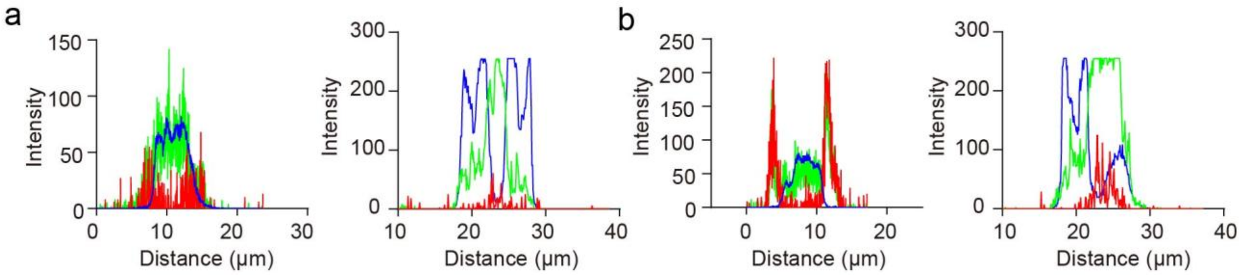


**Figure S10. Fluorescence colocalization analysis across different channels. The image shows confocal microscopy snapshots of cells labeled with specific markers in different fluorescence channels (red, green, and blue). Each curve represents the fluorescence intensity distribution for the corresponding channel, with the X-axis indicating spatial position or scanning distance, and the Y-axis showing fluorescence**

**intensity values.**

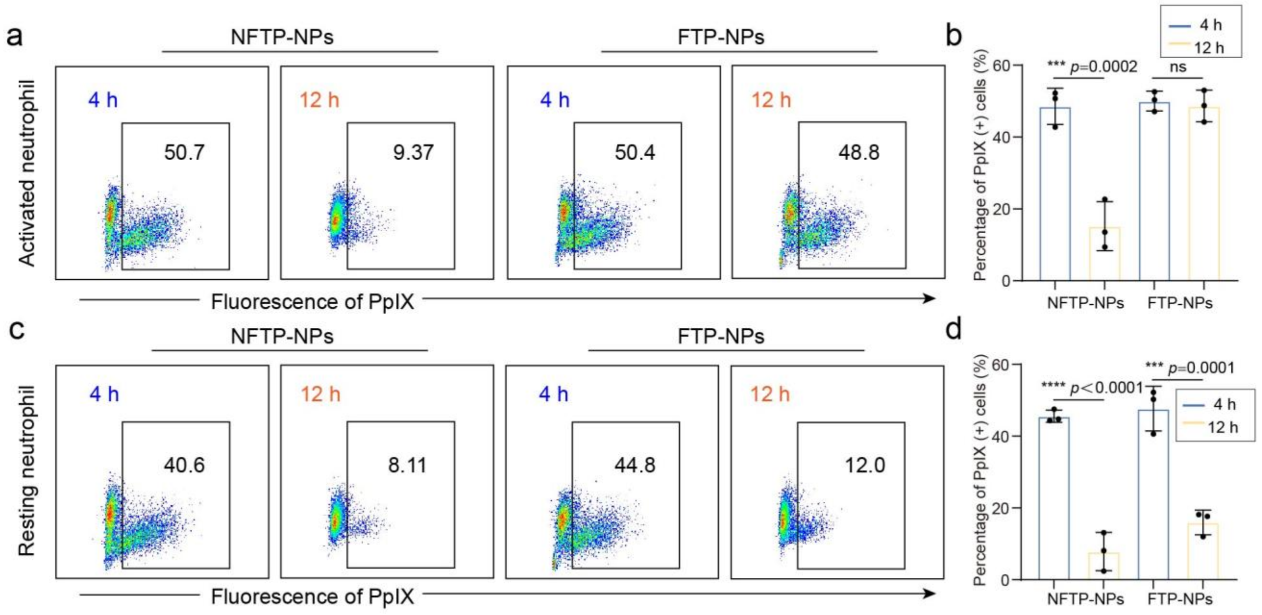


**Figure S11. Retention of FTP-NPs incubation with resting neutrophils and activated neutrophils. (a)** Representative Flow cytometry (FCM) profiles images of PpIX fluorescence in activated neutrophils with various treatments at four h and 12h. The concentration of NFTP-NPs and FTP-NPs was 50 µM. (**b**) Statistics of the percentage of activated neutrophils with PpIX fluorescence signals with different treatments at four h and 12h. The concentration of NFTP-NPs and FTP-NPs was 50 µM. Data are presented as mean ± s.d., $n$ = 3 independent experiments. Statistical significance was calculated by one-way ANOVA, ***$p < 0.001$, n.s. Means no significance. **(c)** Representative Flow cytometry (FCM) profiles images of PpIX fluorescence in resting neutrophils with various treatments at 4 h and 12 h. The concentration of NFTP-NPs and FTP-NPs was 50 µM. (**d**) Statistics of the percentage of resting neutrophils with PpIX fluorescence signals with different treatments at 4h and 12 h. The concentration of NFTP-NPs and FTP-NPs was 50 µM. Data are presented as mean ± s.d., $n$ = 3 independent experiments. Statistical significance was calculated by one-way ANOVA, ***$p < 0.001$, ****$p < 0.0001$.

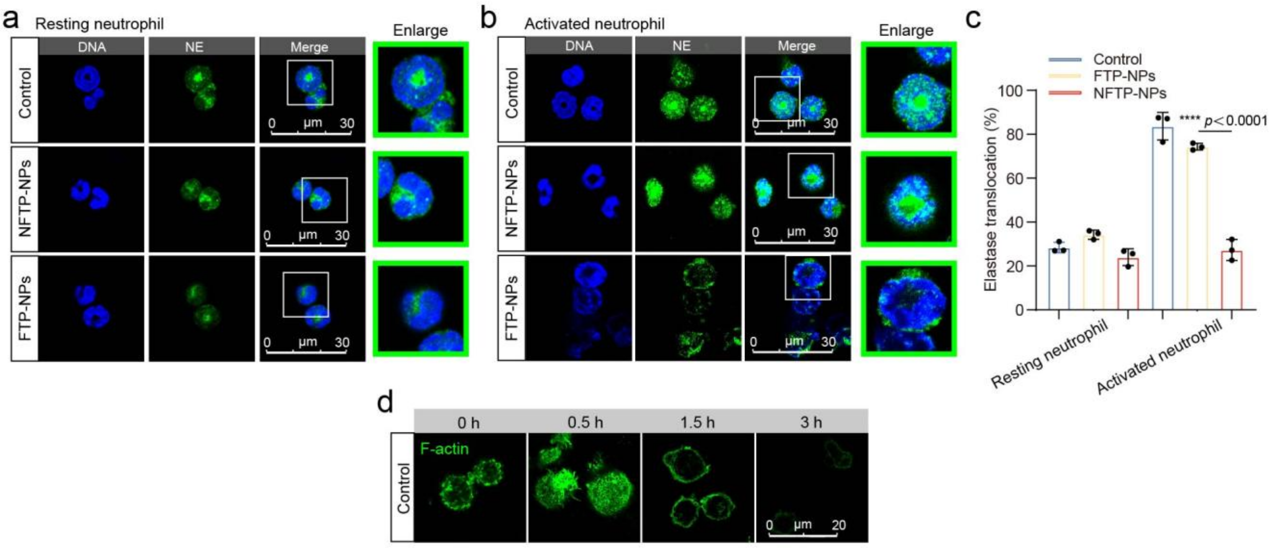


**Figure S12. Lack of NE translocation to the nucleus after inhibition of FTP-NPs.** (**a, b**) Representative images of neutrophils pretreated with FTP-NPs and NFTP-NPs and stimulated with PMA for 0 h and 4 h. Neutrophils were stained for NE (green) and DNA (blue). Scale bar = 30 µm. Data are presented as mean ± s.d., *n* = 3 independent experiments. One-way ANOVA, ****$p$ < 0.0001 calculated statistical significance. (**c**) Quantifying NE translocation to the nucleus at 0 h and 4 h. (**d**) F-actin levels upon PMA stimulation were examined with phalloidin (green) at the indicated time points. Scale bar = 20 µm.

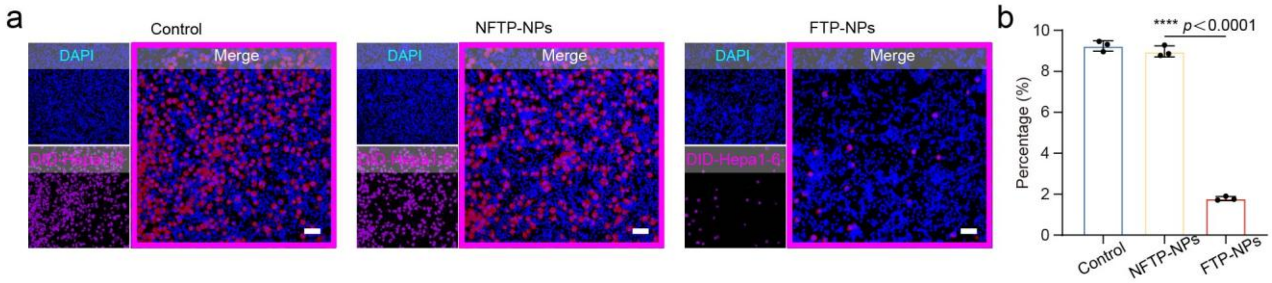


**Figure S13. The blockage of Hepa1-6 cells and neutrophils' interaction by FTP-NPs. (a)** Representative fluorescence images of DID-Hepa1-6 cells and neutrophils with various treatments. Scale bars, 50 µm. The concentration of NFTP-NPs and FTP-NPs was 50 µM. Experiments were repeated three times. (**b**) Flow cytometry (FCM) analysis of DID-Hepa1-6 cells after different treatments. The concentration of NFTP-NPs and FTP-NPs was 50 µM. Data are presented as mean ± s.d., *n* = 3 independent experiments: One-way ANOVA, ****$p$ < 0.0001 calculated statistical significance.

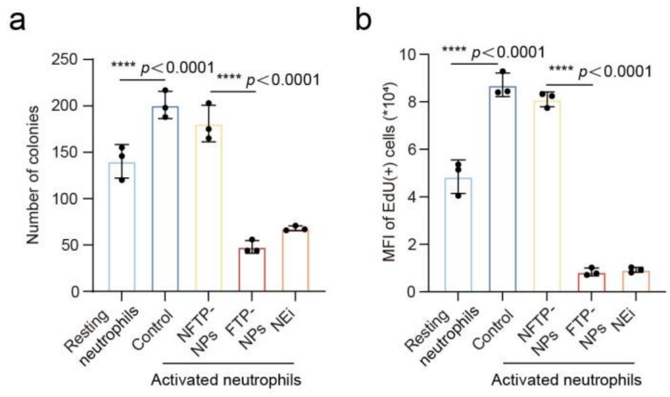


**Figure S14. Effect of activated neutrophils on Hepa1-6 cell proliferation after FTP-NPs.** (**a**) Quantification of the clone formation assay. Data are presented as mean ± s.d., $n = 3$ independent experiments. One-way ANOVA, $^{****}p < 0.0001$ calculated statistical significance. (**b**) The corresponding intracellular mean fluorescence intensity with different treatments. The concentration of NFTP-NPs and FTP-NPs was 50 μM. Data are presented as mean ± s.d., $n = 3$ independent experiments: One-way ANOVA, $^{****}p < 0.0001$ calculated statistical significance.

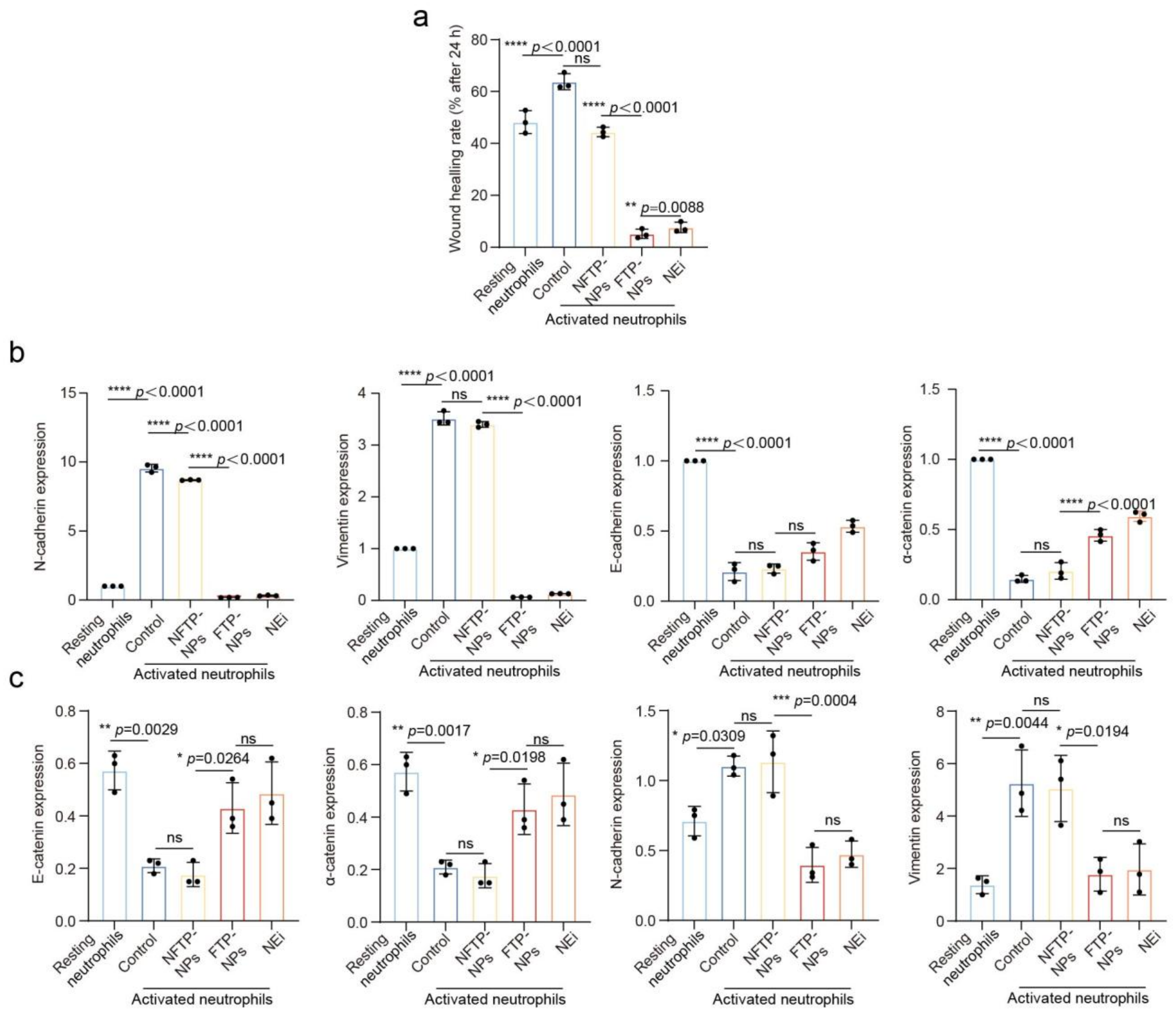


**Figure S15. FTP-NPs effectively suppressed tumor metastasis *in vitro*.** (**a**) Cell scratch quantitative analysis of Hepa1-6 cells after different treatments. Data are presented as mean ± s.d., $n = 3$ independent experiments. Statistical significance was calculated by ANOVA, ****$p < 0.0001$, **$p < 0.010$, n.s. Means no significance. (**b**) Nested polymerase chain reaction (PCR) analysis of Hepa1-6 cells with different treatments. The concentration of NFTP-NPs and FTP-NPs was 50 μM. Data are presented as mean ± s.d., $n = 3$ independent experiments. Statistical significance was calculated by one-way ANOVA, ****$p < 0.0001$, n.s. Means no significance. (**c**) The protein levels of four EMT-related genes after different treatments were determined by western blot. Data are presented as mean ± s.d., $n = 3$ independent experiments. Statistical significance was calculated by ANOVA, ***$p < 0.001$, **$p < 0.010$, n.s. no significance.

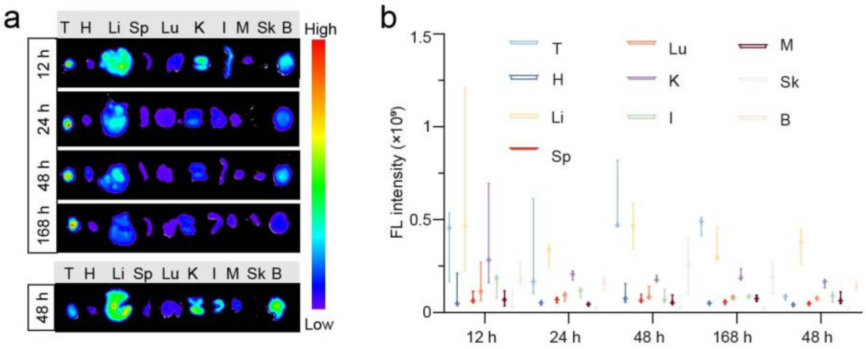


**Figure S16. Time-dependent *ex vivo* fluorescence (FL) images (a) and quantitative analysis (b) of tumor tissues and major organs (heart (H), liver (Li), spleen (Sp), lung (Lu), kidney (K), intestine(I), muscle (M), skin (Sk), and brain (B)) collected at 12, 24, 48, and 168 h post-injection of NFTP-NPs and FTP-NPs.**

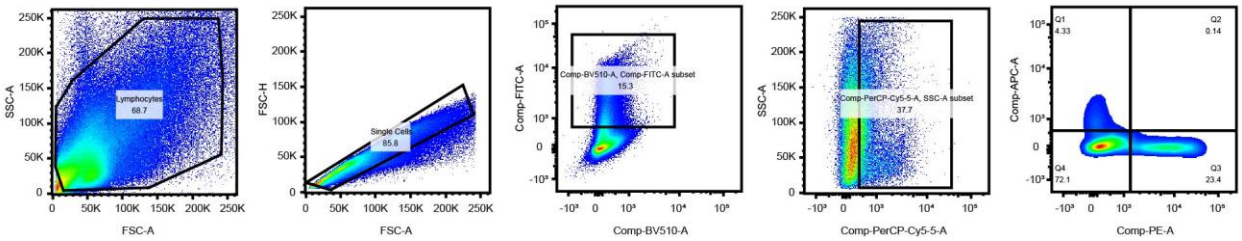


**Figure S17. Gating strategy for T cell analysis. The tumor-bearing mice from each group were euthanized on day 20 post-treatment. Fifty thousand events were collected for each sample in the study.** $CD45^+$ cell counts determined the total T cell number, and $CD8^+$ T cells were then gated within $CD3^+$ T lymphocytes.

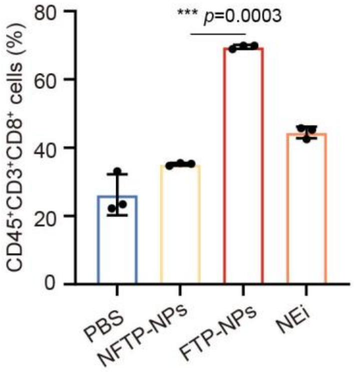


**Figure S18. Corresponding flow cytometric quantification of $CD8^+$T cells ($CD45^+CD3^+CD8^+$, *n* = 3).** Data are presented as mean ± s.d., $n = 3$ independent experiments. Statistical significance was calculated by one-way ANOVA, ***$p < 0.001$.

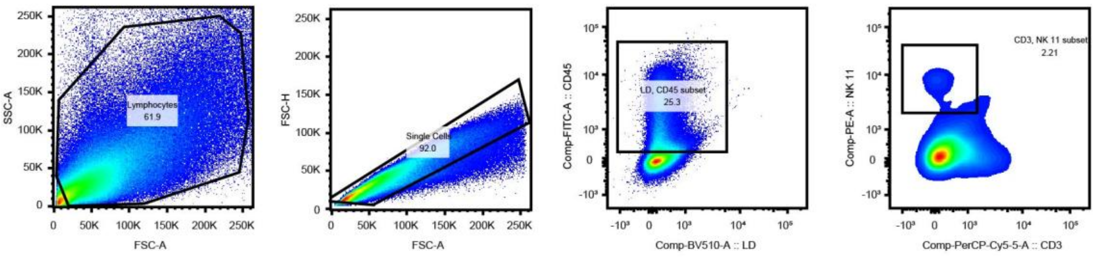


**Figure S19. Gating strategy for NK cells analysis.** The tumor-bearing mice from each group were euthanized on day 20 post-treatment. 50,000 events were collected for each sample in the analysis. CD45+ cell counts determined total NK cell number, and NK1.1$^+$ T cells were then gated within CD3$^-$ T lymphocytes.

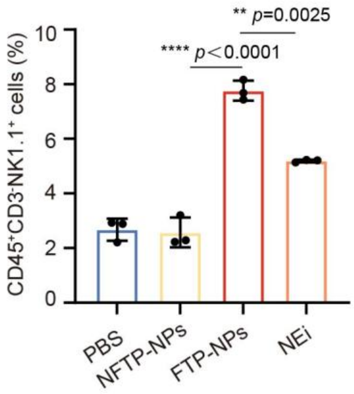


**Figure S20. Corresponding flow cytometric quantification of NK cells (CD45$^+$CD3$^-$CD49b$^+$, $n$ = 3).** Data are presented as mean ± s.d., $n$ = 3 independent experiments. Statistical significance was calculated by one-way ANOVA, ****$p < 0.0001$, **$p < 0.01$.

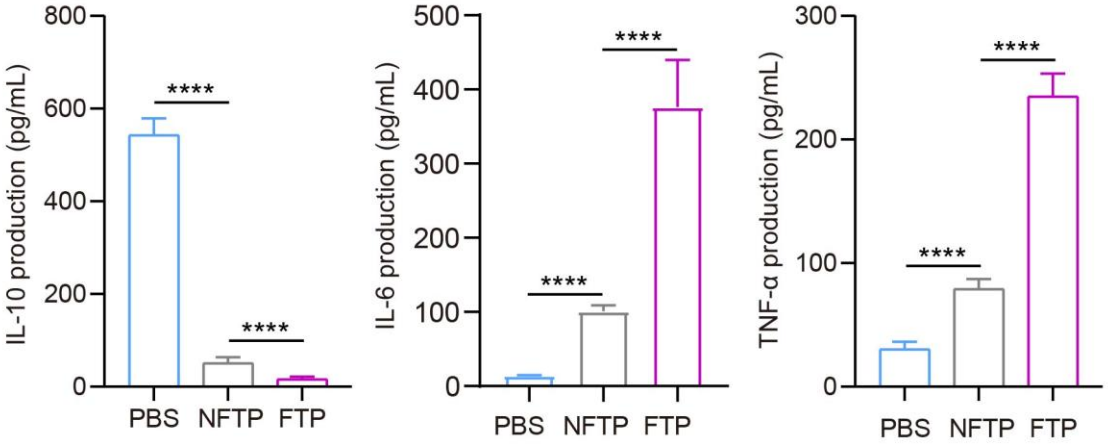


**Figure S21. Gating strategy for MDSCs analysis.** The tumor-bearing mice from each group were euthanized on day 20 post-treatment. 50,000 events were collected for each sample in the analysis. F4/80$^+$ and CD11b$^+$ cell counts determined total macrophage cell numbers, and Gr-1$^+$ cells were further gated within F4/80$^+$ and CD11b$^+$ macrophage cells.

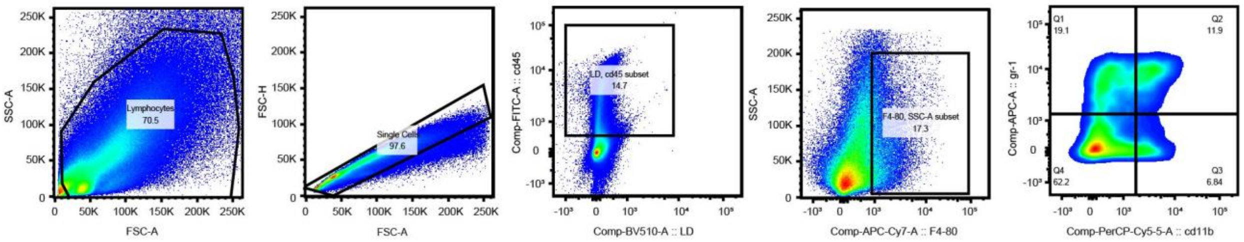


**Figure S22. Gating strategy for MDSCs analysis.** The tumor-bearing mice from each group were euthanized on day 20 post-treatment. 50,000 events were collected for each sample in the analysis. F4/80$^+$ and CD11b$^+$ cell counts determined total macrophage cell numbers, and Gr-1$^+$ cells were further gated within F4/80$^+$ and CD11b$^+$ macrophage cells.

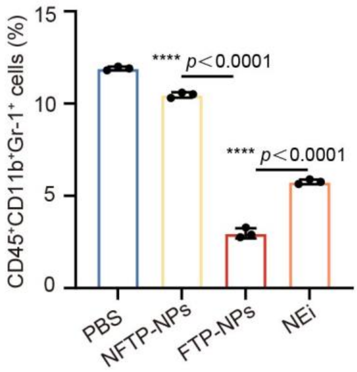


**Figure S23. Corresponding flow cytometric quantification of MDSCs ($CD45^+CD11b^+Ly6G^+$, $n$ = 3).** Data are presented as mean ± s.d., $n$ = 3 independent experiments: One-way ANOVA, ****$p$ < 0.0001 calculated statistical significance.

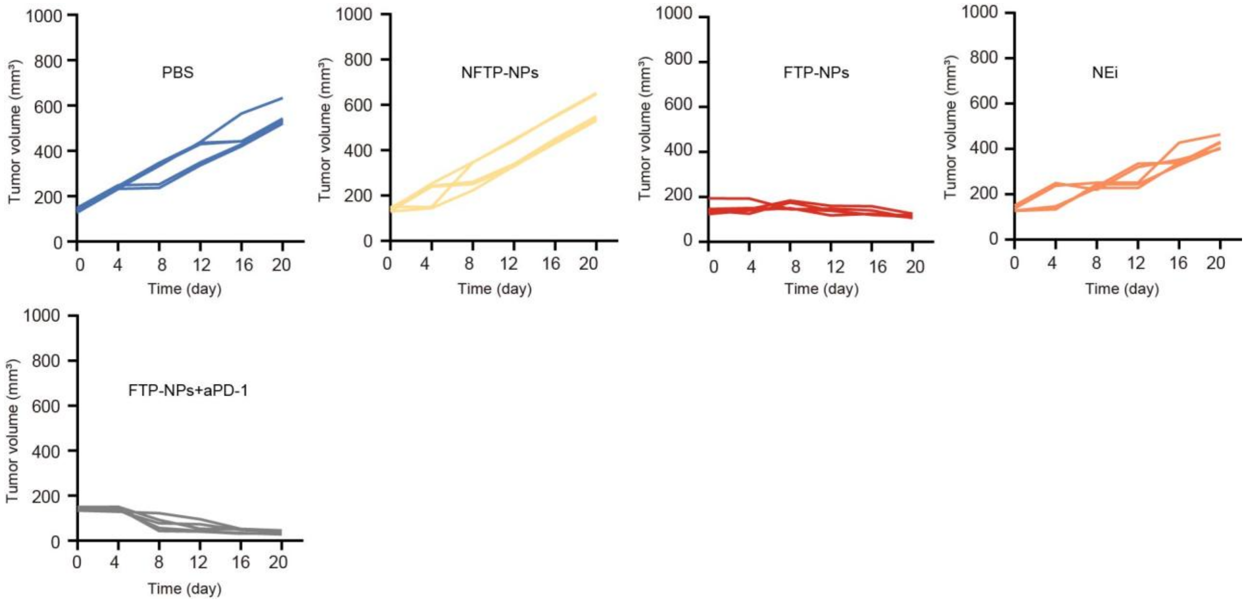


**Figure S24. Individual tumor growth kinetics of Hepa1-6/luc tumor-bearing mice after varied treatment strategies ($n$ = 5).**

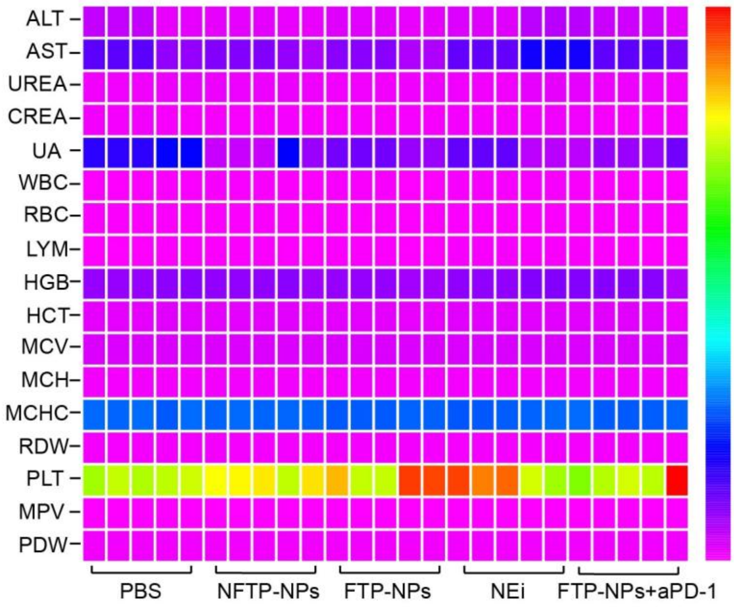


**Figure S25. The biocompatibility of FTP-NPs *in vivo*.** Heat map of routine blood and serum biochemistry analysis of mice after intravenous injection of different NPs ($n$ = 5).

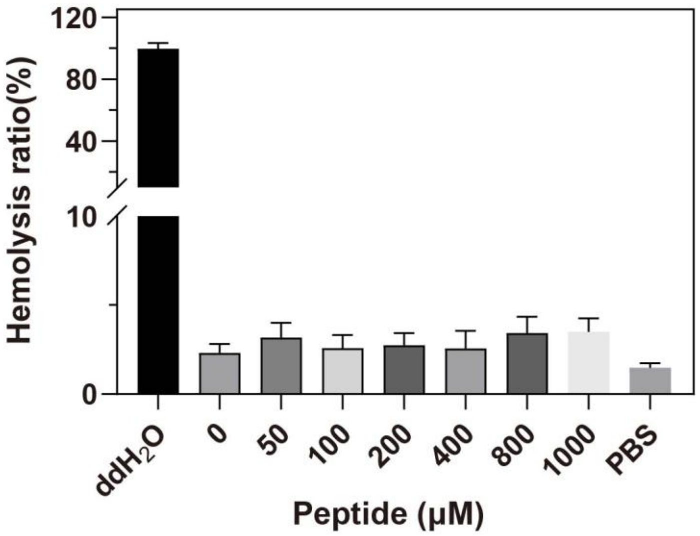


**Figure S26. The biocompatibility of FTP-NPs *in vivo*.** Heat map of routine blood and serum biochemistry analysis of mice after intravenous injection of different NPs ($n$ = 5).

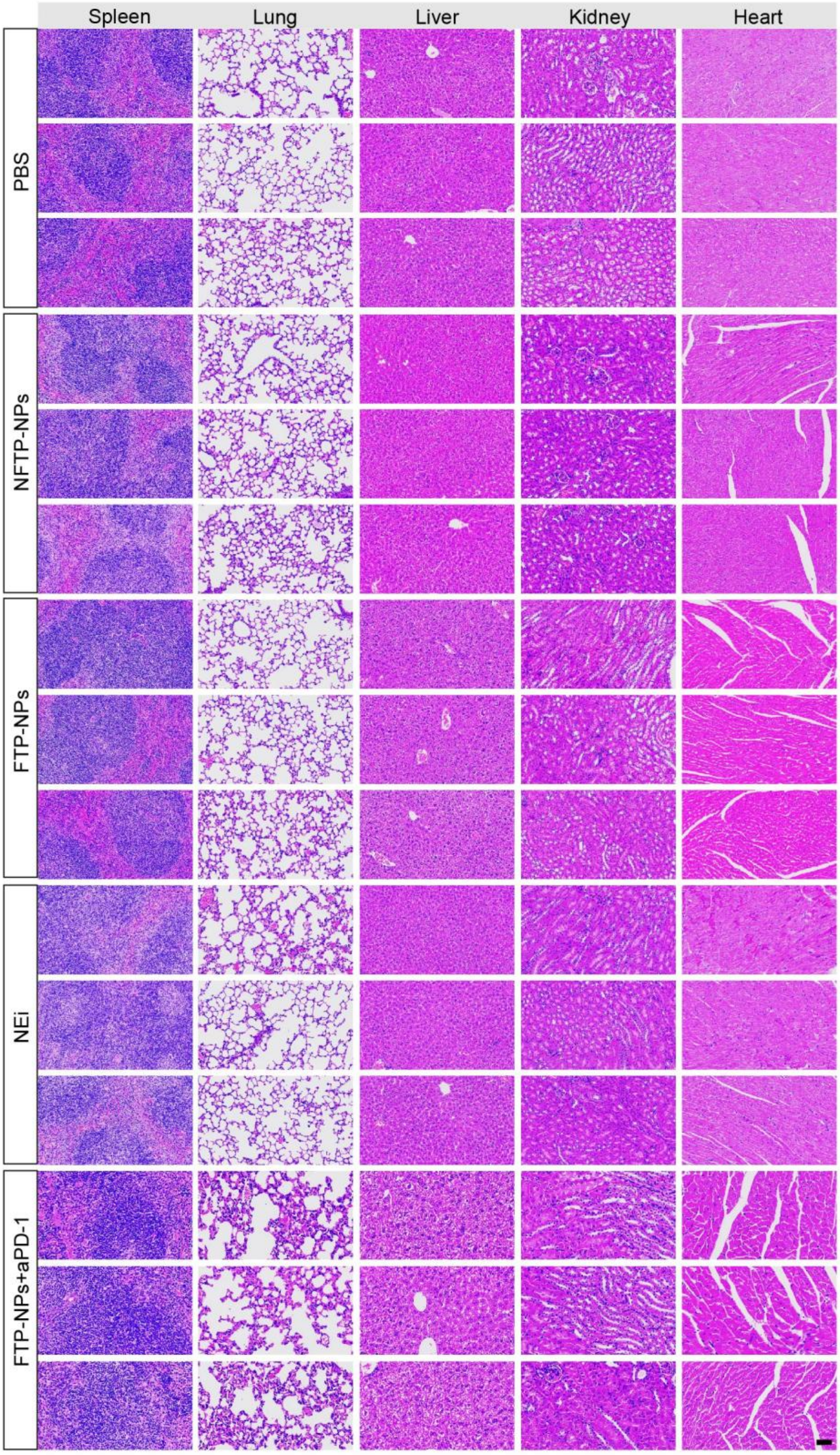


**Figure S27. The biocompatibility of NPs *in vivo*.** H&E staining images of other major organs from the three groups. Scale bar, 40 μm. Experiments were repeated three times.

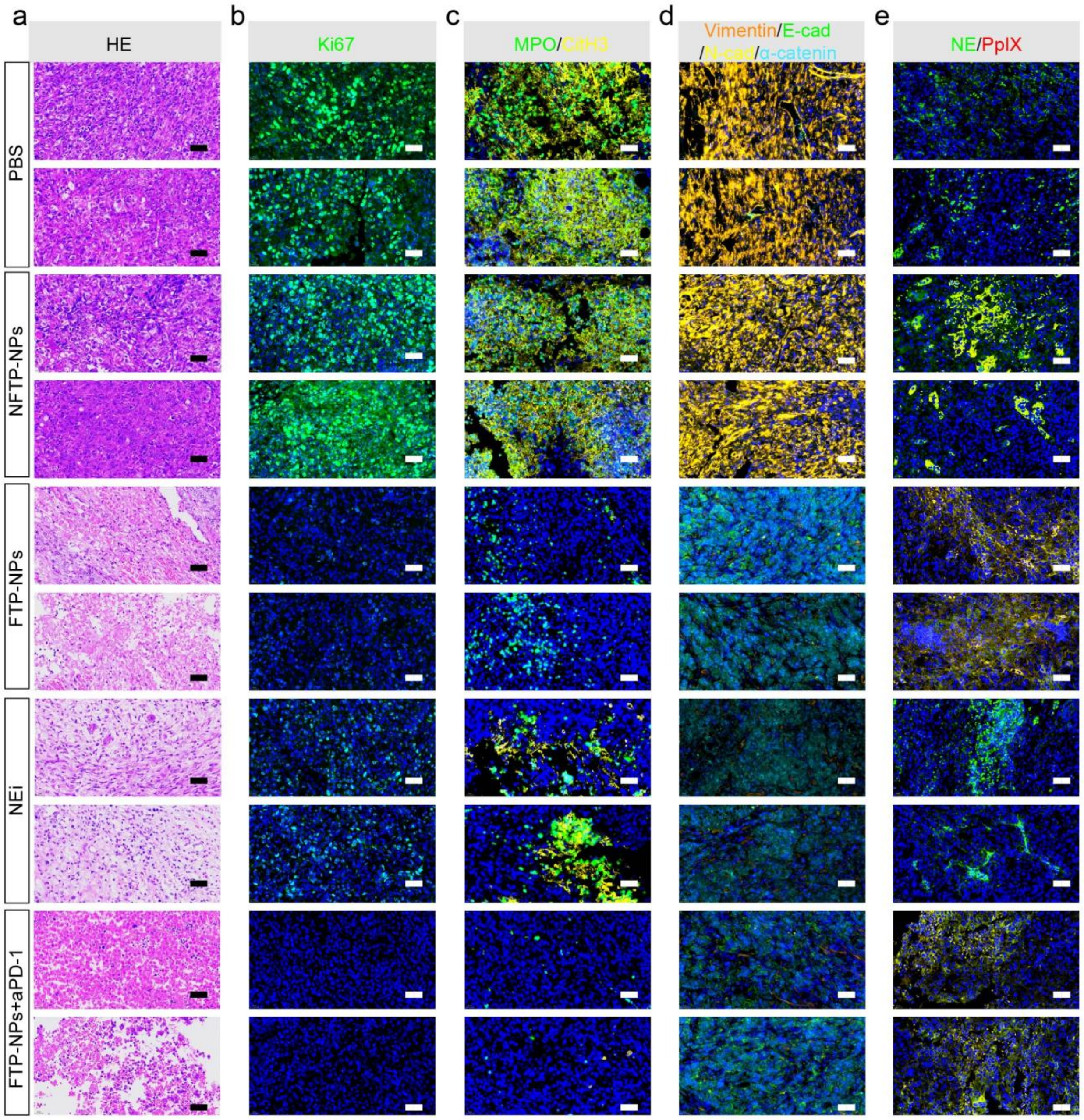


**Figure S28. Tumor treatment efficiency mechanisms of FTP-NPs *in vivo*.** (**a**) H&E staining assay of tumors in the five groups. Scale bar, 40 μm. Experiments were repeated three times. (**b**) IF staining assays of Ki67 in five groups. Scale bar, 40 μm. Experiments were repeated three times. (**c**) IF staining assays of MPO and CitH3 in five groups. Scale bar, 40 μm. Experiments were repeated three times. (**d**) IF staining assays of E-cadherin, α-catenin, N-cadherin, and Vimentin in five groups. Scale bar, 40 μm. Experiments were repeated three times. (**e**) Immunofluorescence of mouse tumor tissues for the co-localization of FTP-NFs and NE (green, NE Protein; red, PpIX of FTP; blue, DAPI; scale bars, 40 μm).

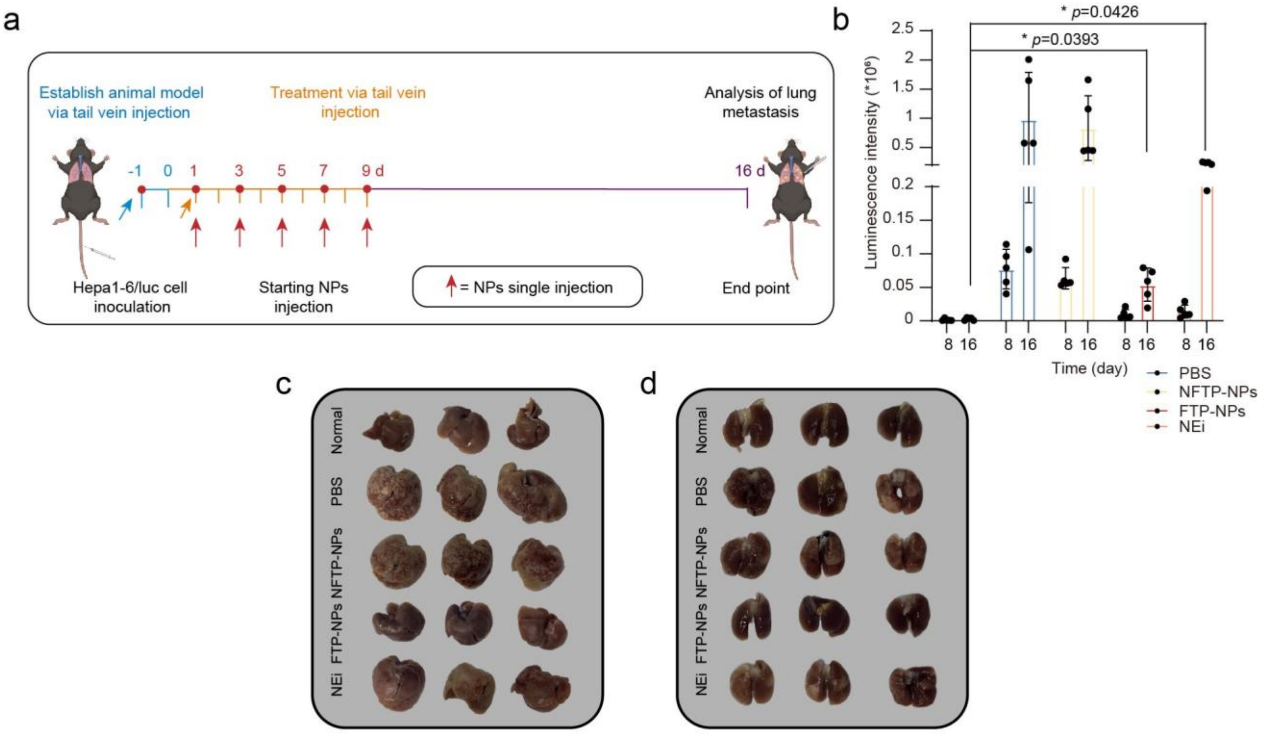


**Figure S29. *In vivo* anti-metastasis efficacy evaluation.** (**a**) Schematic illustration of the experimental approach for FTP-NPs inhibiting lung metastasis ($n$ = 5 per group; the dose of NFTP-NPs and FTP-NPs was 300 μM per injection) for mice. The scheme was created with BioRender.com. (**b**) Fluorescence quantized chart of lung metastasis in Hepa1-6/luc tumor-bearing mice. Data are presented as mean±s.d., $n$ = 5 independent experiments. Statistical significance was calculated by one-way ANOVA, *$p < 0.050$.(**c, d**) Photographs of lung and liver metastasis in Hepa1-6/luc tumor-bearing mice treated with PBS, NFTP-NPs, FTP-NPs, and NEi for 16 days ($n$ = 5).

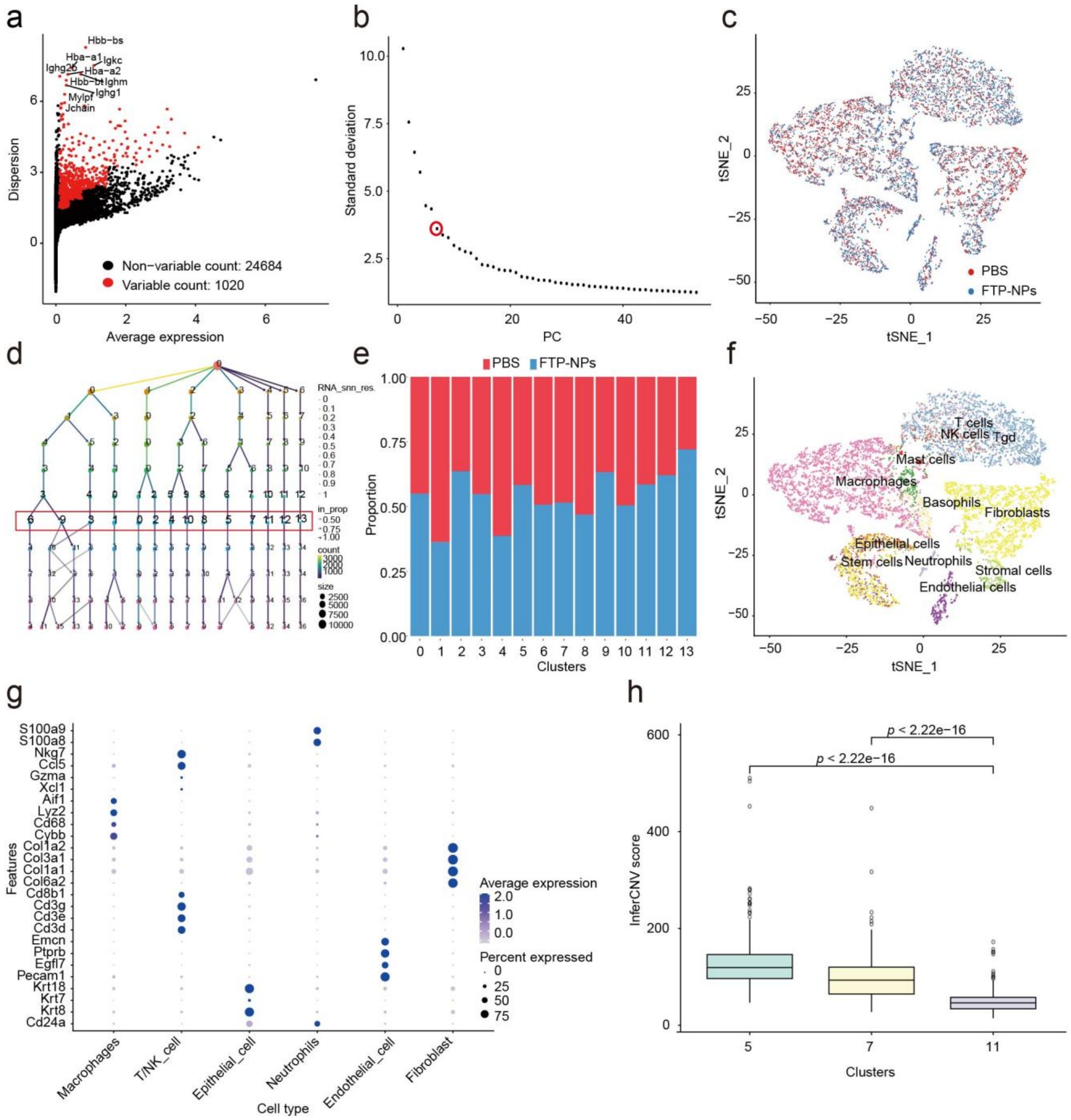

**Figure S30. Single-cell clustering and cell type annotation.** (**a**) Screening of 1,020 highly variable genes in the cells. (**b**) Selecting the 7 best principal components. (**c**) The cells in the PBS and FTP-NPs samples are primarily clustered into 6 cell clusters. (**d**) The resolution for unsupervised clustering was set between 0.1 and 1, with a resolution of 0.6 being the optimal. At this resolution, the cells were clustered into 14 smaller cell clusters. (**e**) The proportions of the 14 cell clusters in the PBS and FTP-NPs samples, with blue representing the FTP-NPs sample and red representing the PBS sample. (**f**) The SingleR method was used to identify the cell types of the 14 cell clusters. (**g**) The Cell Taxonomy method was used to identify the cell types of the 14 cell clusters. (**h**) The inferCNV algorithm was used to infer the gene CNV scores of the cells. The x-axis represents

endothelial cells (cell cluster 11) and epithelial cells (cell clusters 5 and 7), while the y-axis represents the gene CNV scores of the cells, the P-values were calculated with the Student's t test.

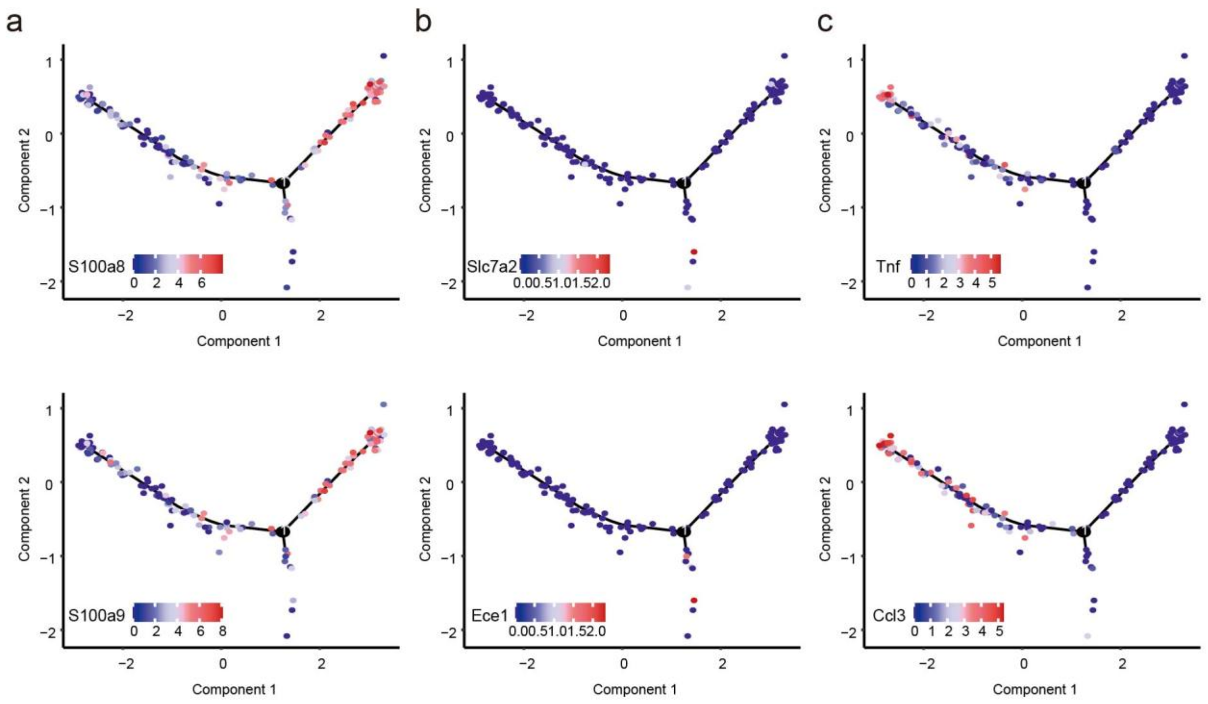


**Figure S31. Pseudotime analysis of neutrophil-specific high-expression genes across three states.** (**a**) Expression of neutrophil-specific high-expression genes S100a8 and S100a9 in state 1 during pseudotime progression. (**b**) Expression of neutrophil-specific high-expression genes Slc7a2 and Ece1 in state 2 during pseudotime progression. (**c**) Expression of neutrophil-specific high-expression genes Tnf and Ccl3 in state 3 during pseudotime progression.

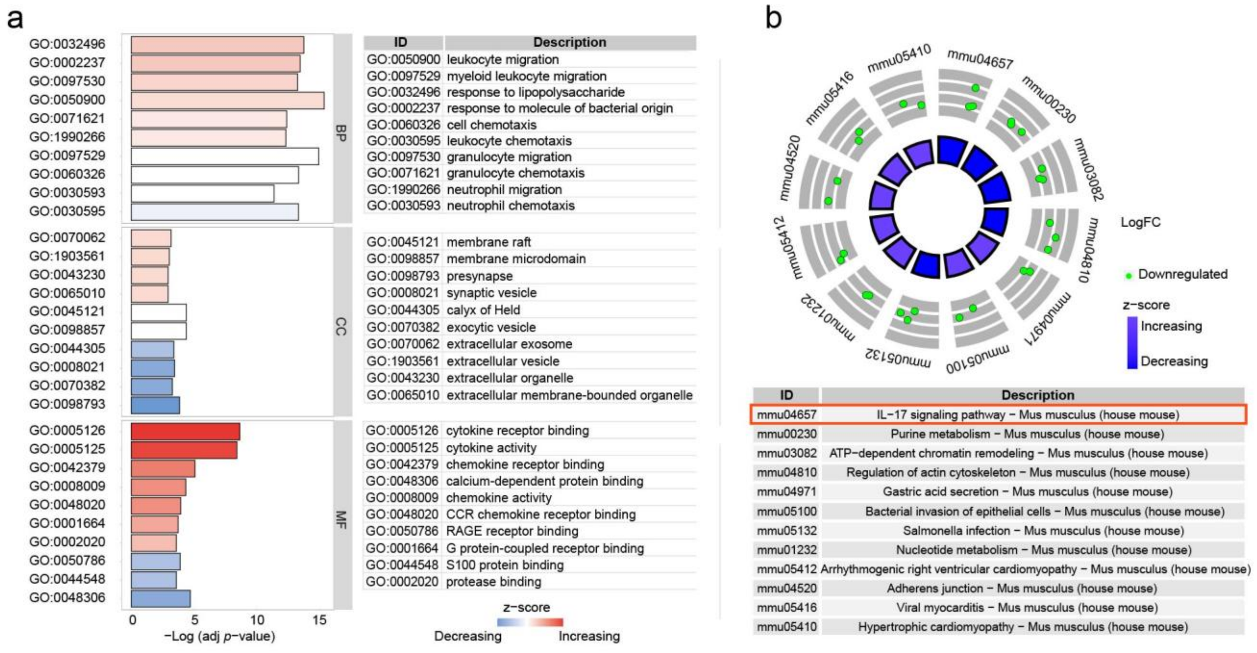


**Figure S32. Enrichment analysis of neutrophil-specific high-expression genes across three states**. (i) GO functional enrichment analysis of differentially expressed genes between neutrophils in state 3 and state 1. The x-axis represents the significance level, and the y-axis represents GO terms. Red indicates GO terms that are promoted. (j) KEGG pathway enrichment analysis of differentially expressed genes between neutrophils in state 3 and state 1. Pink indicates genes that are highly expressed in neutrophils in state 3, and red indicates KEGG pathways that are promoted.

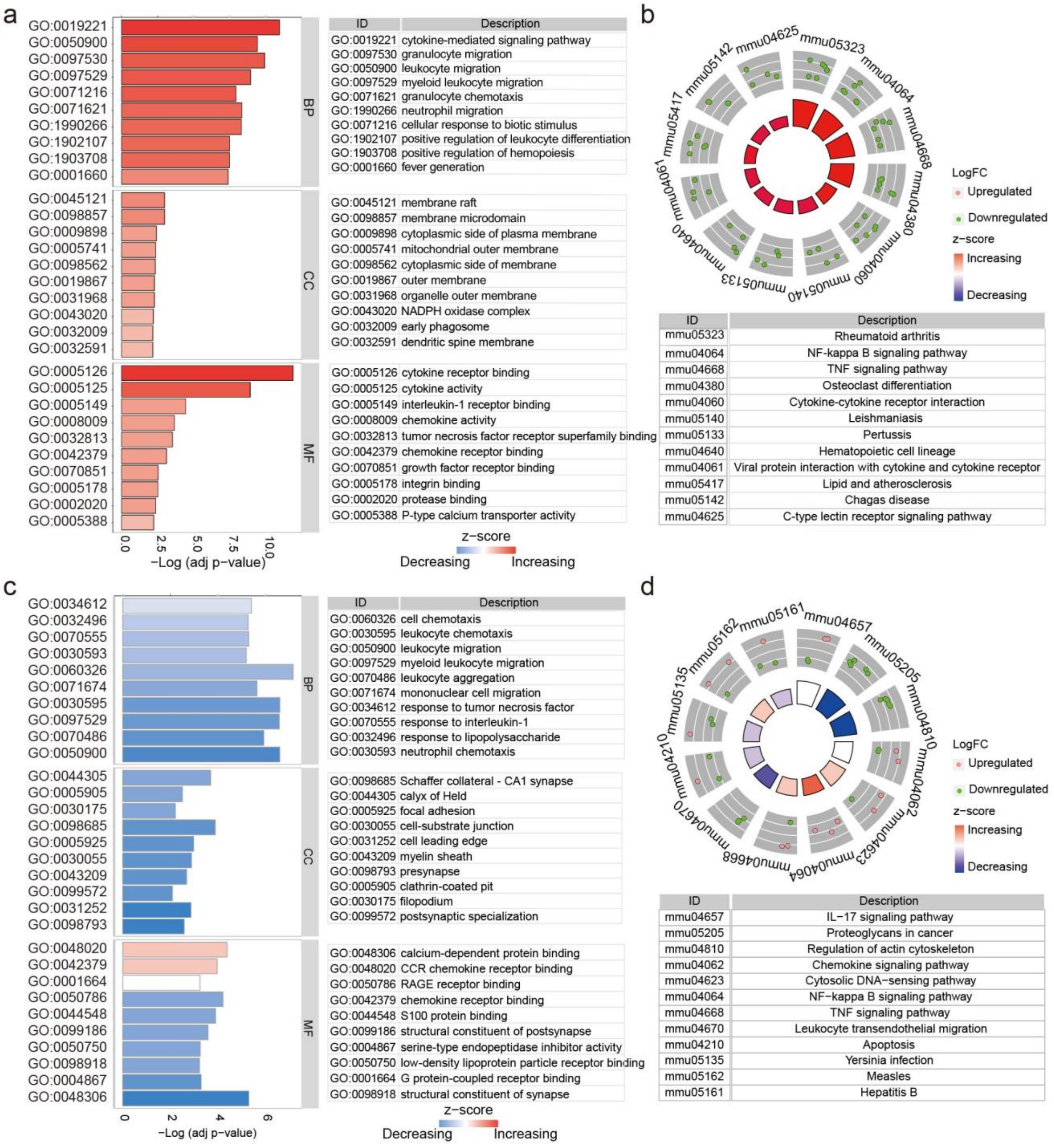


**Figure S33. Comparison of neutrophil function across the three states.** GO functional enrichment and KEGG pathway enrichment analysis of differentially expressed genes between neutrophils in state 3 and state 2 (a, b), and between neutrophils in state 2 and state 1 (c, d).

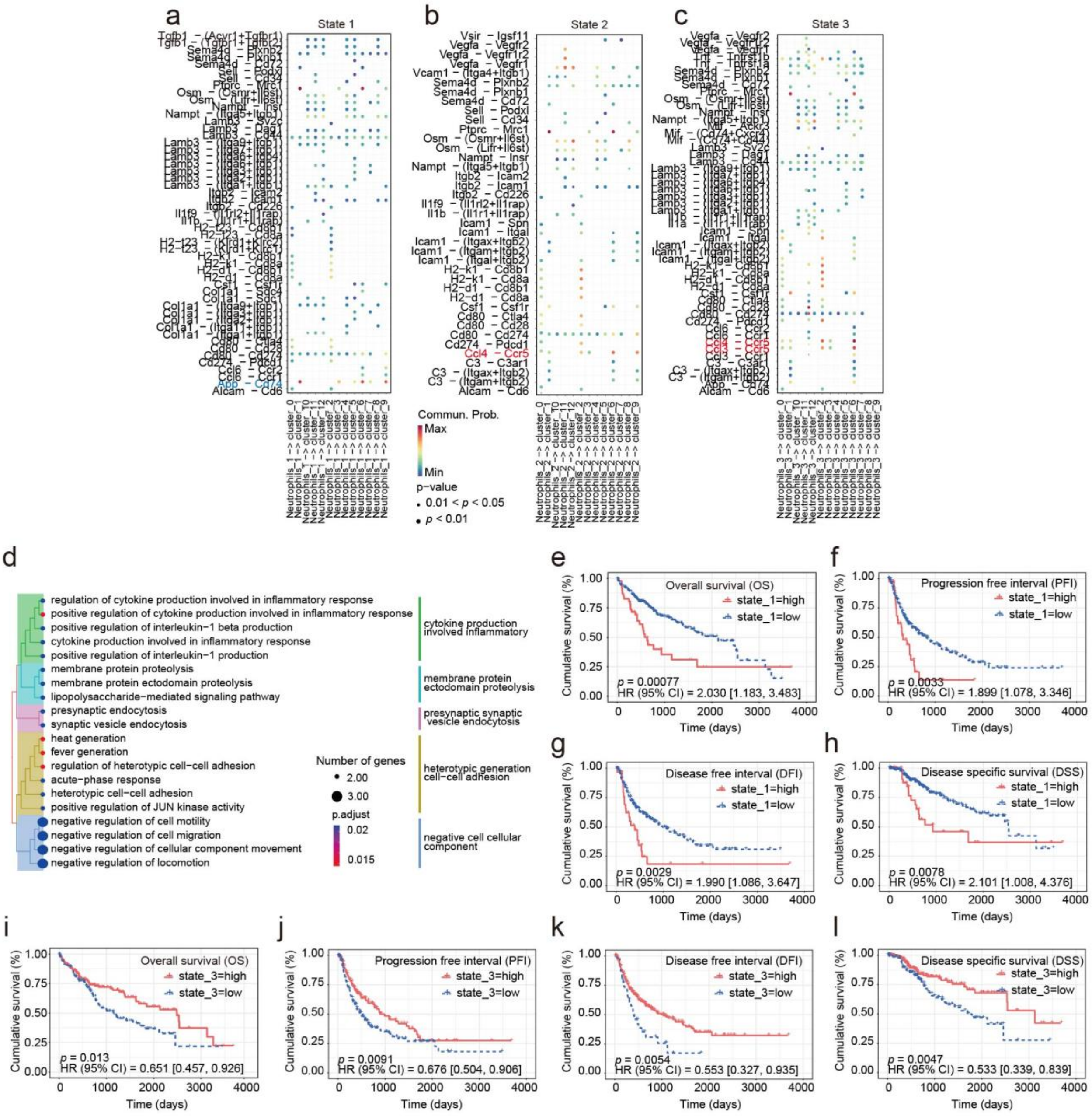


**Figure S34. Neutrophil intercellular communication analysis and prognostic correlation with TCGA-LIHC samples.** (**a-c**) Intercellular communication regulation of neutrophils in State 1, State 2, and State 3 by other cell clusters in the TIME. The x-axis represents the intercellular communication pairs, and the y-axis represents the receptor-ligand pairs. (**d**) GO functional enrichment analysis of genes highly correlated with Thbs1 expression. The color of the dots represents the significance level of the enrichment results. (**e-h**) Kaplan–Meier survival curves of OS (**i**), PFI (**j**), DFI (**m**), and DSS (l) comparing the high and low content of state 1 neutrophils in the TCGA-LIHC cohort. (**i-l**) Kaplan–Meier survival curves of OS (**i**), PFI (**j**), DFI (**k**), and DSS

(l) comparing the high and low content of state 3 neutrophils in the TCGA-LIHC cohort; the P-values were calculated with the log-rank test.

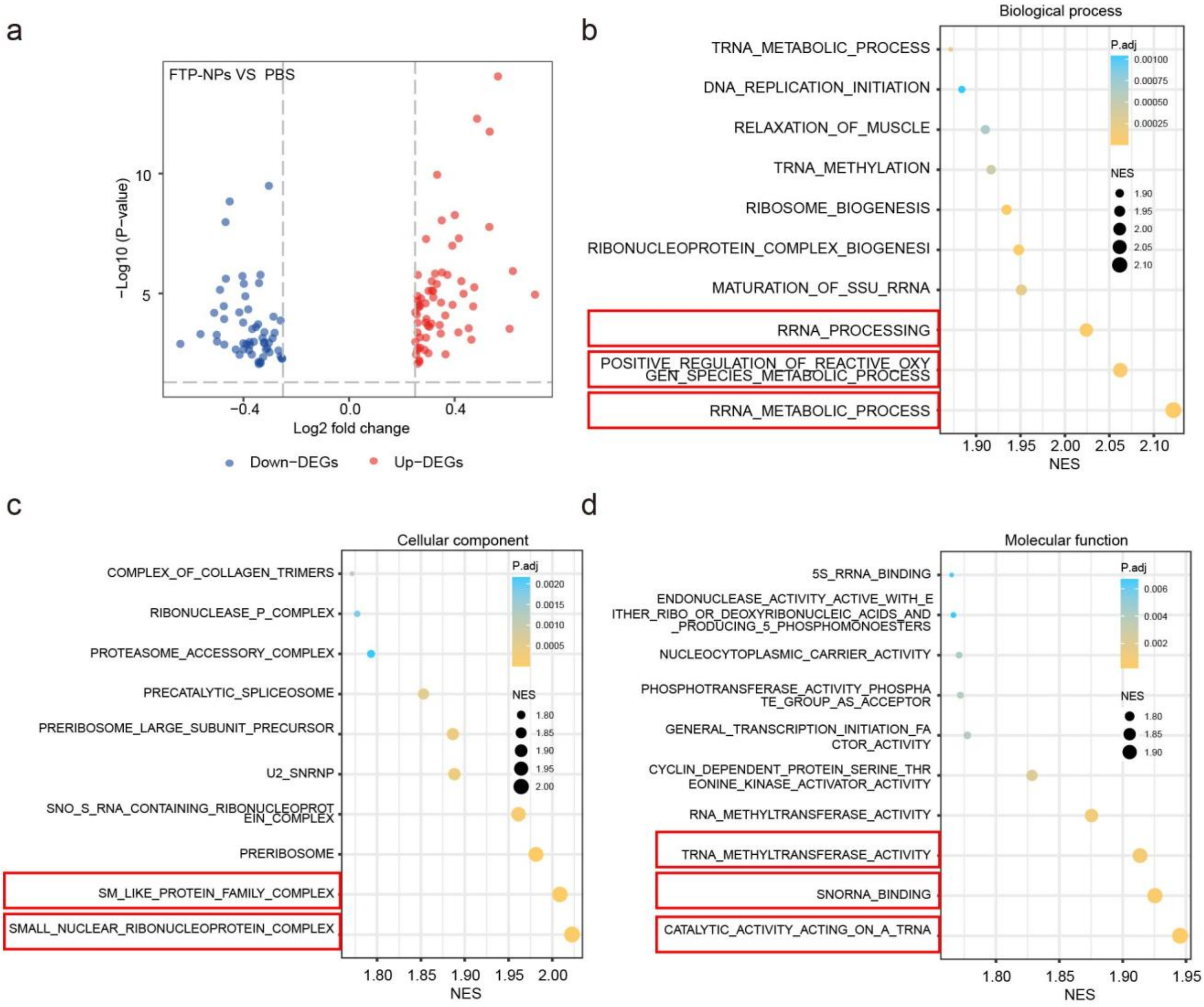


**Figure S35. Biological process changes between tumor cells in the FTP-NPs and PBS groups.** (**a**) Volcano plot of differentially expressed genes between tumor cells in the FTP-NPs and PBS groups, with the x-axis representing log2FC and the y-axis representing -log10 (*p-value*). Red dots represent genes that are highly expressed in tumor cells of the FTP-NPs group. (**b-d**) GO functional annotation analysis of differentially expressed genes. The node color represents the significance of the enrichment results, and the node size reflects the number of enriched genes. The x-axis represents -log10 (*p-value*), and the y-axis represents BP (Biological Process), CC (Cellular Component), and MF (Molecular Function).

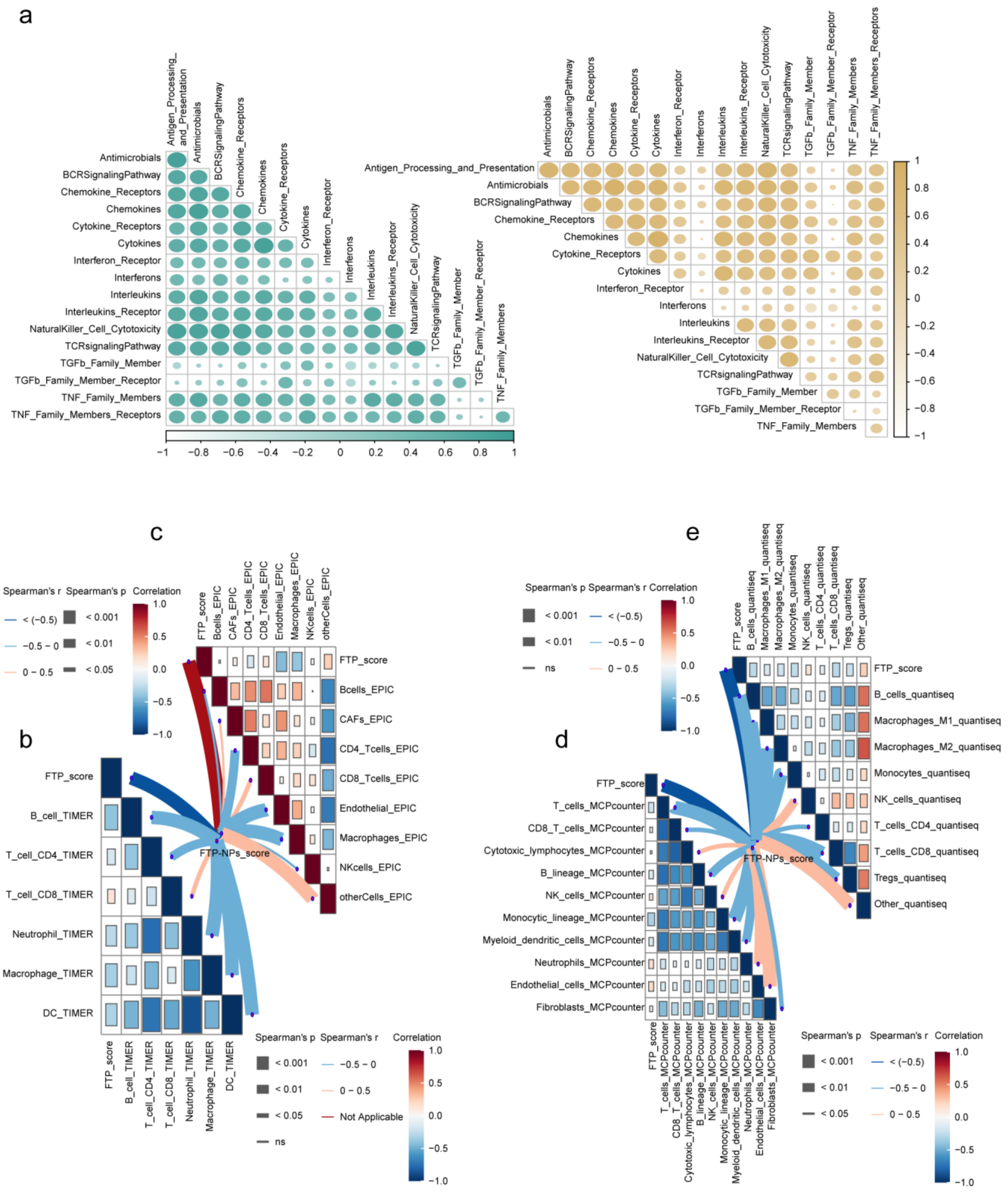

**Figure S36. Immune landscape characterization of HCC patients stratified by FTP-NPs score.** (**a**) Correlations of 17 immune signatures in the two FTP-NPs subgroups. (**b-e**) Pearson correlations between FTP-NPs score and fractions of immune cells using TIMER (b), EPIC (c), MCPcounter (d) and quanTIseq (e) quantification method. *, $p < 0.050$; **, $p < 0.010$; ***, $p < 0.001$; ****, $p < 0.0001$; ns, no significance.

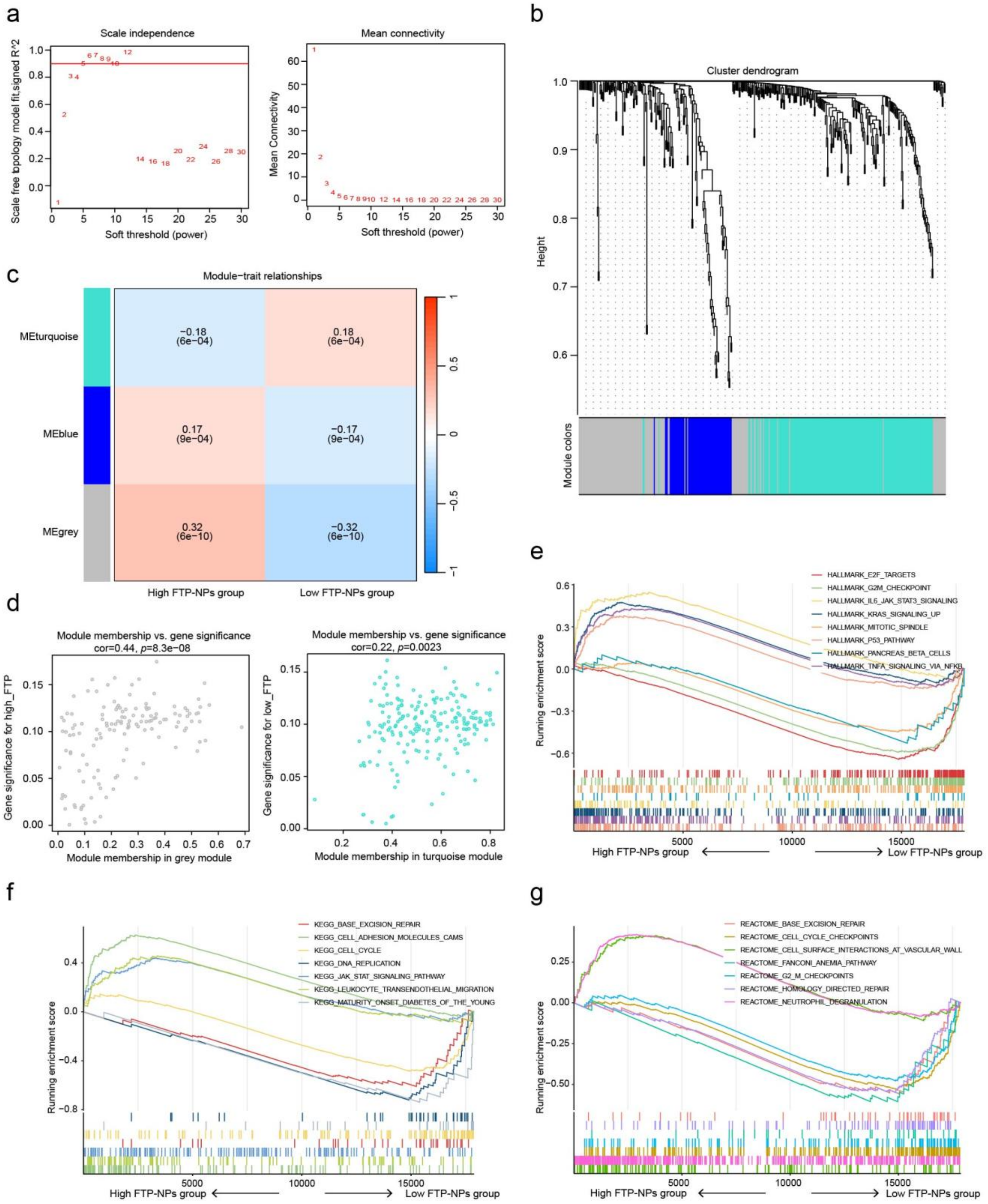


**Figure S37. Identification of FTP-related gene modules via WGCNA and pathway enrichment analysis in the TCGA-LIHC cohort.** (**a**) Scale-free fitting index (left) and average connectivity (right) for different soft-thresholding powers β. The red line represents a correlation coefficient of 0.9. (**b**) Hierarchical clustering

dendrogram of co-expression modules, with different colors representing different modules. (**c**) Heatmap showing the correlations between the high FTP-NPs group and the low FTP-NPs group related gene modules, with each cell containing the corresponding correlation and p-value. (**d**) The scatter plot of the correlation between the grey module and the high FTP-NPs group-related genes (left). The scatter plot of the correlation between the turquoise module and the low FTP related genes (right). (**e-g**) GSEA plot of significant Hallmark pathways (e), KEGG pathways (f), and Reactome pathways (g) in comparison between the FTP-NPs group and the low FTP-NPs group.

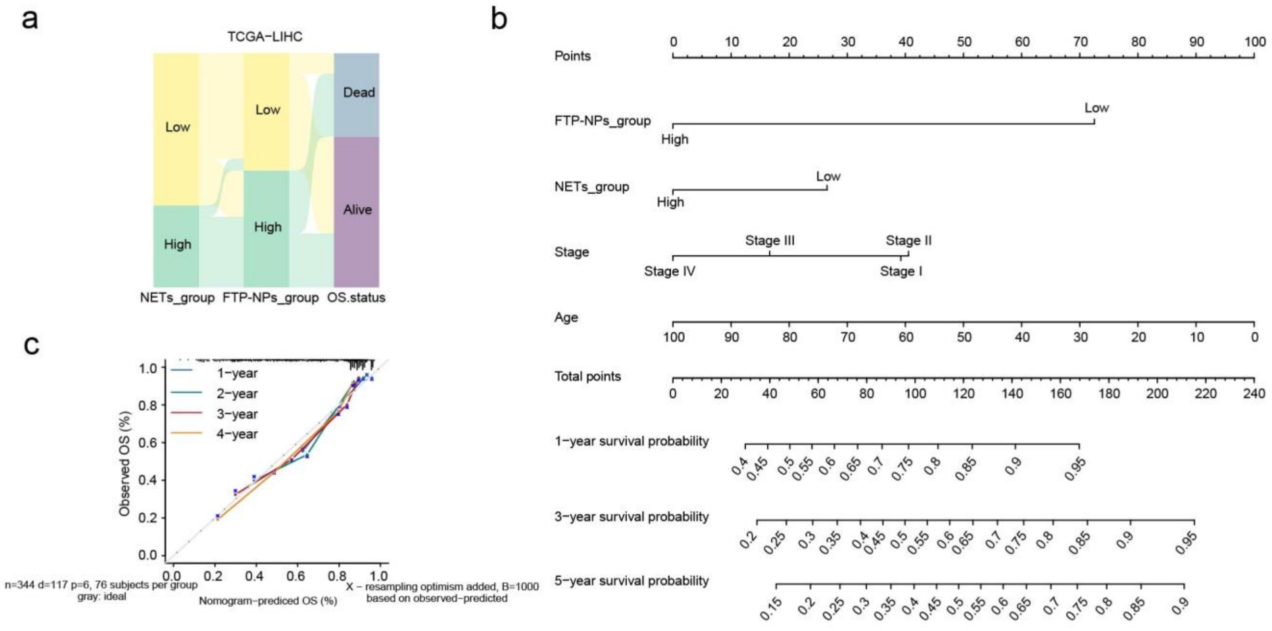


**Figure S38. Development and assessment of a nomogram model for the prognosis of HCC.** (**a**) A Sankey plot elegantly illustrated the intricate connections between the NETs score, FTP-NPs group, and OS status, revealing their synergistic effects on risk assessment. (**b**) Integrated Nomogram combined the NETs score, FTP-NPs group, and clinical features for HCC prognosis. (**c**) The calibration curve demonstrated predictive precision of the nomogram.

**Table S1. The top five best docking conformations of FTP and NFTP with NE were analyzed.**

| Mol | Score | RMSD (Root mean square deviation) after refinement | Conformational energy | Placement energy | Score 1 energy | Refinement energy | Score 2 energy |
|---|---|---|---|---|---|---|---|
| FTP-Rank-1 | -12.5945 | 3.52586 | -131.566 | 98.65938 | 8.791847 | -84.9342 | -12.5945 |
| FTP-Rank-2 | -12.4042 | 5.857288 | -120.064 | 84.03509 | 8.610281 | -87.673 | -12.4042 |
| FTP-Rank-3 | -11.7217 | 3.880744 | -113.638 | 85.8605 | 6.721291 | -79.5068 | -11.7217 |
| FTP-Rank-4 | -11.4631 | 4.853666 | -141.425 | 56.97803 | 6.625461 | -73.5177 | -11.4631 |
| FTP-Rank-5 | -11.445 | 3.341936 | -135.795 | 26.82232 | 2.886133 | -76.0127 | -11.445 |
| NFTP-Rank-1 | -13.879 | 4.734949 | -142.288 | 169.7198 | 11.84576 | -97.3014 | -13.879 |
| NFTP -Rank-2 | -12.6952 | 4.970714 | -93.6776 | 187.1084 | 12.1502 | -85.8094 | -12.6952 |
| NFTP -Rank-3 | -12.4833 | 4.680527 | -132.079 | 106.8876 | 7.253531 | -83.8109 | -12.4833 |
| NFTP-Rank-4 | -11.5196 | 4.905543 | -180.778 | 84.20881 | 9.689094 | -74.6847 | -11.5196 |
| NFTP-Rank-5 | -11.2891 | 3.776316 | -144.804 | 256.3924 | 20.03745 | -75.3516 | -11.2891 |
